# Deposition of the Membrane Attack Complex in Healthy and Diseased Human Kidneys

**DOI:** 10.3389/fimmu.2020.599974

**Published:** 2021-02-11

**Authors:** Jacob J. E. Koopman, Mieke F. van Essen, Helmut G. Rennke, Aiko P. J. de Vries, Cees van Kooten

**Affiliations:** ^1^ Division of Renal Medicine, Department of Medicine, Brigham and Women’s Hospital, Harvard Medical School, Boston, MA, United States; ^2^ Division of Nephrology, Department of Internal Medicine, Leiden University Medical Center, Leiden, Netherlands; ^3^ Division of Renal Pathology, Department of Pathology, Brigham and Women’s Hospital, Harvard Medical School, Boston, MA, United States

**Keywords:** biopsy, C5b-9 (membrane attack complex [MAC]), histopathology, immunofluorescence, immunohistochemistry, renal, clinicopathological correlation, glomerular disease

## Abstract

The membrane attack complex—also known as C5b-9—is the end-product of the classical, lectin, and alternative complement pathways. It is thought to play an important role in the pathogenesis of various kidney diseases by causing cellular injury and tissue inflammation, resulting in sclerosis and fibrosis. These deleterious effects are, consequently, targeted in the development of novel therapies that inhibit the formation of C5b-9, such as eculizumab. To clarify how C5b-9 contributes to kidney disease and to predict which patients benefit from such therapy, knowledge on deposition of C5b-9 in the kidney is essential. Because immunohistochemical staining of C5b-9 has not been routinely conducted and never been compared across studies, we provide a review of studies on deposition of C5b-9 in healthy and diseased human kidneys. We describe techniques to stain deposits and compare the occurrence of deposits in healthy kidneys and in a wide spectrum of kidney diseases, including hypertensive nephropathy, diabetic nephropathy, membranous nephropathy, IgA nephropathy, lupus nephritis, C3 glomerulopathy, and thrombotic microangiopathies such as the atypical hemolytic uremic syndrome, vasculitis, interstitial nephritis, acute tubular necrosis, kidney tumors, and rejection of kidney transplants. We summarize how these deposits are related with other histological lesions and clinical characteristics. We evaluate the prognostic relevance of these deposits in the light of possible treatment with complement inhibitors.

## Introduction

The membrane attack complex is the end-product of the three complement pathways: the classical, lectin, and alternative pathway. Activation of these pathways leads to generation of C5 convertase, which cleaves C5 into C5a and C5b. While C5a functions as an anaphylatoxin, C5b binds to C6, C7, C8, and multiple copies of C9, constituting C5b-9, also known as the membrane attack complex. This complex forms a pore through a pathogen’s or cell’s membrane—structurally and functionally similar to perforin produced by cytotoxic T cells—and disrupts the pathogen’s or cell’s integrity. Formation of C5b-9 can cease incompletely without anchoring to a membrane, in which case it circulates as a soluble complex with vitronectin or clusterin, referred to as sC5b-9 (1, 2). Both C5b-9 and sC5b-9 promote inflammation and thrombosis.

Activation of the complement pathways plays an essential role in the pathogenesis of kidney diseases, but the pathways are involved to varying extents. Glomerular deposition of immune complexes predominantly activates the classical pathway in lupus nephritis, the lectin pathway in primary membranous nephropathy, and both the lectin and alternative pathway in IgA nephropathy (3). The extent to which C5b-9 is formed varies as well. The alternative pathway is activated in both C3 glomerulonephritis and dense deposit disease but leads to more C5b-9 in the former (4–6).

With the clinical development of targeted complement inhibitors (7–9), it is essential to know which parts of the complement pathways go awry in specific kidney diseases. Eculizumab, a monoclonal antibody binding C5, inhibiting its cleavage, and thus preventing formation of C5b-9, is used to treat aHUS and some cases of lupus nephritis, C3 glomerulonephritis, dense deposit disease, IgA nephropathy, and transplant rejection (10–17). Inhibitors of other complement factors are being developed (7–9). Although eculizumab seems to benefit particularly patients in whom much C5b-9 is formed (4, 11, 18, 19), it remains uncertain which patients benefit from which complement inhibitor.

Levels of sC5b-9 in blood and urine are elevated in various kidney diseases and associated with their activity and severity (4, 10–12, 20–33). Yet, measurement of sC5b-9 in blood or urine is cumbersome due to its easy formation *in vitro* and short half-life (34). Deposition of C5b-9 in kidneys is thought to better reflect the involvement of its formation in the pathogenesis of kidney diseases (35, 36). The membrane-bound form may more accurately indicate complement activation and disease activity than its circulating soluble form, as has been shown for other complement factors in SLE (10, 11, 37, 38). Deposition may also be associated with prognosis, similarly to deposition of C4d in IgA nephropathy and kidney transplants (15, 26, 39). Lastly, deposition indicates that C5a has been formed locally, which promotes inflammation and thrombosis through the C5a receptors. This is increasingly recognized as a pathogenetic process and possible treatment target in various kidney diseases and transplant rejection (10–12, 14, 15, 17, 26, 32, 40).

**Figure 1:**
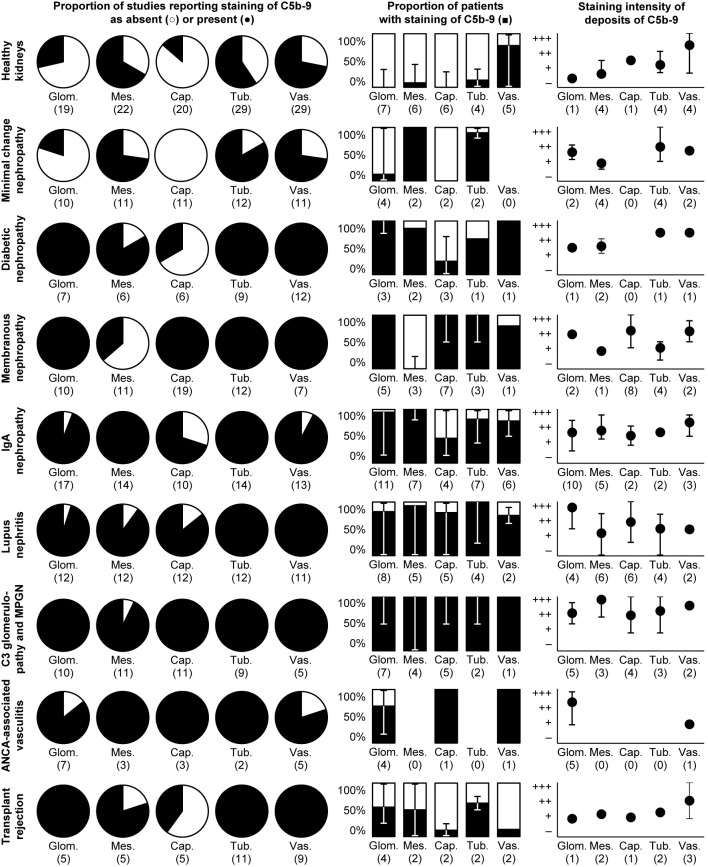
Deposits of C5b-9 in healthy and diseased human kidneys. Pie charts show the proportion of studies that reported staining of C5b-9 as absent (light) or present (dark). Bar charts show the medians of the proportions of patients reported to exhibit staining. Scatter charts show the median staining intensities in these patients. All charts show data separately for staining in the glomerulus as a whole (glom.), in the mesangium (mes.), along the glomerular capillary wall (cap.), along the tubular basement membrane (tub.), or in the extraglomerular vascular wall (vas.). Error bars show the lowest and highest reported values. Numbers of studies are indicated between brackets. Some studies reported only part of the data shown, explaining differences in the numbers of studies between pie, bar, and scatter charts. Nothing is indicated if the data were never reported. Detailed data per study are listed in **Supplementary Table 2**. Membranous nephropathy excludes studies conducted specifically on secondary membranous nephropathy. IgA nephropathy excludes studies conducted specifically on IgA vasculitis with nephritis. Data on these diseases and on glomerular basement membrane diseases, hypertensive nephropathy, interstitial nephritis, acute tubular necrosis, and kidney tumors are only listed in **Supplementary Table 2** because of a paucity of data. ANCA: antineutrophil cytoplasmic antibody; MPGN: membranoproliferative glomerulonephritis.

Since deposition of C5b-9 in human kidneys has never been compared across individual studies, it remains uncertain under which conditions, in which diseases, in which areas, and in which quantities it can be found (35). To aid in this understanding, we provide a review of studies on deposition of C5b-9 in healthy and diseased human kidneys. We describe our search strategy and methods, the methodological characteristics of the 141 included studies, and the findings of these studies in the **Supplementary Material**, which may be used as a reference for future research. We summarize the main findings derived from these studies in **Figure 1** and **Table 1**. We illustrate possible correlations between deposition of C5b-9 and histological lesions or clinical characteristics in the other figures. We detail the findings in the text, separately for healthy kidneys, nonimmunological kidney diseases, kidney diseases due to deposition of immune complexes, kidney diseases due to activation of the alternative pathway, vasculitis, general patterns of kidney injury, kidney tumors, and kidney transplantation. We discuss the findings in general in a closing discussion.

**Table 1:** Histological lesions and clinical characteristics correlated with deposits of C5b-9 in diseased human kidneys.

	Localization of deposits		
	Glomerulus	Tubules	Vascular wall
Hypertensive nephropathy	Glomerulosclerosis		Loss of vascular smooth muscle cells; arteriosclerosis
Diabetic nephropathy	Mesangial expansion; glomerulosclerosis; IFTA; severity of nephropathy; type of diabetes; creatinine; albuminuria	Interstitial inflammation; IFTA; urine biomarkers of tubular injury; creatinine; albuminuria	Loss of vascular smooth muscle cells; vascular AGEs; arteriosclerosis; severity of nephropathy; creatinine; albuminuria
Minimal change nephropathy	Glomerulosclerosis	IFTA	Arteriosclerosis
Membranous nephropathy	Mesangial hypercellularity; capsular adhesions; glomerulosclerosis; proteinuria; disease progression	Interstitial inflammation; interstitial fibrosis; creatinine	Arteriosclerosis
IgA nephropathy	Mesangial expansion and hypercellularity; endocapillary hypercellularity; capsular adhesion; crescents; thrombotic microangiopathy; glomerulosclerosis; interstitial inflammation; IFTA; age; creatinine; proteinuria; nephrotic syndrome; disease progression	Interstitial inflammation; IFTA; creatinine; proteinuria; nephrotic syndrome; disease progression	Thrombotic microangiopathy; arteriosclerosis
Lupus nephritis	Histological activity and chronicity indices; glomerulosclerosis; blood pressure; proteinuria; serum C3 and C4; lack of treatment effect	Interstitial inflammation; interstitial fibrosis	Arteriosclerosis
C3 glomerulopathy	eGFR		
Membranoproliferative glomerulonephritis type I	Glomerulosclerosis; serum sC5b-9; disease progression	Interstitial fibrosis; disease progression	Arteriosclerosis
Hypertension- associated thrombotic microangiopathy	Proteinuria; plasma complement activity		
ANCA-associated vasculitis	Mesangial expansion; creatinine; proteinuria	Interstitial inflammation; interstitial fibrosis; creatinine; lack of treatment effect	
Interstitial nephritis		Interstitial inflammation; IFTA	Interstitial inflammation; IFTA; arteriosclerosis
Acute tubular necrosis		IFTA; degenerative abnormalities of the tubular basement membrane	
Kidney transplant rejection	eGFR; Banff score; transplant survival	IFTA; anti-ABO antibodies; transplant survival	Arteriosclerosis

Histological lesions and clinical characteristics found to correlate with deposits of C5b-9 in different localizations in the kidney are indicated separately for different kidney diseases, as discussed in more detail in the text. Characteristics found not to correlate are only discussed in the text.

AGEs, advanced glycation end-products; ANCA, antineutrophil cytoplasmic antibody; eGFR, estimated glomerular filtration rate; IFTA, interstitial fibrosis and tubular atrophy.

## Staining Techniques

### Antibodies Against C5b-9

Around 1980, antibodies against C5b-9 were developed for immunofluorescent and immunoperoxidase staining. These antibodies recognize neoepitopes that arise when individual complement factors combine and change their conformation to form C5b-9 (1, 2). When C6 and C7 bind newly formed C5b, they expose a lipophilic tail that anchors to a membrane. C8 then binds this complex and reshapes to penetrate the membrane. Finally, eighteen copies of C9 integrate into the complex and penetrate the membrane to form an asymmetrical and flexible pore (41–43). The neoepitopes recognized by the antibodies are almost always exposed on polymerized C9 (44–49) and sometimes on incomplete forms lacking C9 (50–52). **Table 2** provides an overview of the antibodies that were used in the included studies to stain C5b-9 in kidneys.

**Table 2:** Selective antibodies used to stain C5b-9 in human kidneys.

Name	Clonality	Source	Binding									
			C5	C6	C7	C8	C9	Poly-C9	Incomplete C5b-9^a^	Soluble C5b-9	Membrane-bound C5b-9	Ref.
ab55811	Polyclonal	Rabbit	Unkn.	Unkn.	Unkn.	Unkn.	Unkn.	Unkn.	Unkn.	Unkn.	Unkn.	(53, 54)
aE11 or M0777	Monoclonal	Mouse	–	–	–	–	±	+	+	+	+	(46, 51)
Anti-C5b-9(m)	Polyclonal	Rabbit	–	–	–	–	–	Unkn.	Unkn.	+	+	(55, 56)
Anti-MAC	Polyclonal	Rabbit	–	–	–	–	–	Unkn.	Unkn.	+	+	(57)
Anti-MAC-neo	Polyclonal	Rabbit	–	–	–	–	–	Unkn.	+	+	+	(52)
bC5 or A239	Monoclonal	Mouse	–	–	–	–	±	+	±	+	+	(46)
B7	Monoclonal	Mouse	–	–	–	–	±	+	Unkn.	+	+	(58, 59)
Kolb 1975^b^	Polyclonal	Rabbit	–	–	–	–	–	Unkn.	+	+	+	(50)
PolyC9-MA	Monoclonal	Mouse	–	–	Unkn.	–	–	+	–	Unkn.	+	(44)
WU-7,2	Monoclonal	Mouse	–	–	–	–	±	–	Unkn.	+	+	(48, 60)
WU-13,15	Monoclonal	Mouse	Unkn.	–	–	Unkn.	±	–	–	+	+	(48, 60)
X197	Monoclonal	Mouse	Unkn.	Unkn.	Unkn.	–	+	+	–	Unkn.	+	(47, 49)
Xia 1988^b^	Monoclonal	Mouse	–	–	–	–	–	Unkn.	Unkn.	+	+	(61, 62)
3B1	Monoclonal	Mouse	–	–	–	–	–	+	–	+	+	(45)
1B4	Monoclonal	Unkn.	Unkn.	Unkn.	Unkn.	Unkn.	–	+	Unkn.	+	+	(63)

All antibodies against C5b-9 used for staining of C5b-9 in the included original studies, as specified per study in **Supplementary Table 1**, are indicated. References to studies on their binding selectivity are given.

^a^Incomplete forms of C5b-9 without C9, either soluble or membrane-bound, commonly referred to as C5b-6, C5b-7, and C5b-8.

^b^Names used in **Supplementary Tables 1** and **2** for antibodies without a specific name.

–, no binding; ±, weak binding; +, strong binding; poly-C9, polymerized C9; unkn., unknown.

Staining should be interpreted in the context of the selectivity of the antibodies, which is limited insofar they also bind incomplete forms of C5b-9, such as those lacking C9 or polymerized C9 lacking C5b, as shown in **Table 2**. These incomplete forms occur independently of C5b-9, both on membranes and in blood, and may have similar although smaller cytolytic or inflammatory effects (1, 2, 64). C5b-9 should, therefore, be stained with a monoclonal antibody that recognizes a neoepitope on C5b-9 but not its individual components and preferably not its incomplete forms.

### Membrane-Bound Versus Soluble C5b-9

The antibodies cannot make a distinction between C5b-9 that has anchored a membrane or sC5b-9 that has remained circulating (65), as apparent from **Table 2**. Unlike membrane-bound C5b-9, the lipophilic parts of sC5b-9 are shielded from membranes as they are capped by vitronectin and clusterin (1, 66).

Several studies tried to distinguish both types of C5b-9 by costaining vitronectin, originally called S-protein. This circulating protein binds incomplete forms of C5b-9, interrupts its complete formation, and prevents membrane binding (1, 2). Colocalization was therefore thought to identify soluble sC5b-9 that had deposited in the kidney without anchoring to a membrane (67–71). Indeed, deposits of vitronectin were seldomly seen in the absence of C5b-9 (70, 72, 73). However, vitronectin can also bind complete membrane-bound C5b-9 (60, 64, 65, 74), had a similar distribution as the membrane-bound regulatory factor CD59 (58), was found without C5b-9 in healthy kidneys (72, 75), did not always colocalize with C5b-9 in diseased kidneys (61, 67, 72, 73, 76, 77), colocalized with immune deposits in diseased kidneys when C5b-9 was deficient (78), was associated with the extracellular matrix (75), and was localized in the subepithelial space which it cannot reach when bound to soluble sC5b-9 (67, 73, 75, 79). Therefore, costaining of vitronectin cannot be used as an indicator of sC5b-9.

Clusterin, a protein with a similar function as vitronectin (1, 2), was less often studied. It was present in the vascular wall in healthy kidneys and both the glomerulus and vascular wall in diseased kidneys, colocalized with C5b-9 according to some but not to other studies (58, 69, 70, 73, 80). By contrast, CD59, also known as protectin, is a membrane-bound protein that binds and inhibits membrane-bound C5b-9 only (1, 2). It can bind the lipophilic parts of C8 or C9 in incomplete forms of C5b-9, preventing their penetration of the membrane and integration of other copies of C9 into the complex (2, 41–43). Reports on its presence in healthy and diseased kidneys were inconclusive (58, 81–87).

Apart from protective factors like CD59, cells can resist the cytolytic effects of C5b-9 by shedding parts of their membranes to which C5b-9 has bound as extracellular vesicles. Extracellular vesicles are also shed in various other pathological and physiological processes and can subsequently be targeted by C5b-9. Extracellular vesicles are present in blood, urine, and kidney tissue (88, 89). Antibodies cannot distinguish C5b-9 on extracellular vesicles from C5b-9 bound to cells or circulating sC5b-9.

### Comparisons of Different Staining Techniques

Some studies used a combination of antibodies against individual components, such as C6 and C9, instead of a selective antibody to stain deposits of C5b-9 (**Supplementary Table 1**). These complement factors are, in contrast to C5b-9, ever-present in blood (45, 46, 55, 60). Some of them—notably C5, C6, and C9—may be present in the glomerulus when others are not (90–92). As a result, individual complement factors could stain when C5b-9 did not (44, 52, 57, 78) and could stain with varying intensities (44, 52, 57, 67, 76, 93, 94), as illustrated in **Figures 3A, B**. Staining intensities of C6 and C7 were generally lowest (44, 52, 94), while that of C9 often resembled that of C5b-9 (18, 44, 94–97).

Only one study compared different selective antibodies against C5b-9—among which aE11, anti-C5b-9(m), and B7—and found identical glomerular staining (58). Results obtained with different antibodies in included studies might vary slightly, but we could not discern a relation with their selectivities, though comparisons were hampered by a paucity of data (**Supplementary Tables 2** and **3** and **Supplementary Figures 1** and **2**).

**Figure 2:**
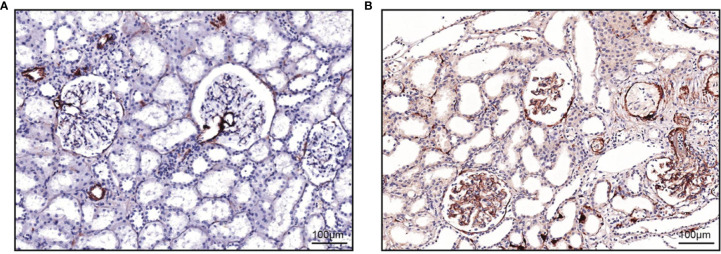
Staining of C5b-9 in a healthy and a diseased kidney. Examples of staining of C5b-9 from our laboratory are shown. **(A)** In a healthy kidney, staining was present in the vascular pole of the glomerulus and the vascular wall of extraglomerular arteries and focally with less intensity along Bowman’s membrane and the tubular basement membrane. This tissue was obtained with a biopsy from a living donor before kidney transplantation. **(B)** In a kidney of a patient with aHUS, staining was present along the glomerular capillary wall, in the vascular wall of extraglomerular arteries and focally along Bowman’s membrane and the tubular basement membrane. This tissue was obtained with a clinically indicated biopsy. Both tissues were fixed, paraffin-embedded, and sectioned. After deparaffinization (xylol and ethanol) and antigen retrieval (PBS-0.1% Proteinase XXIV, P8038, Sigma), sections were washed and endogenous peroxidase was blocked (PBS, 0.1% NaN_3_, 1% H_2_O_2_) for 30 min at room temperature. Sections were washed (PBS) and incubated with mouse anti-human C5b-9 (2 µg/ml, aE11, HM2167, Hycult Biotech, Uden, the Netherlands) or an isotype control (mouse IgG2a, 2 µg/ml, X0943, Dako, Jena, Germany) in PBS with 1% BSA over night at room temperature. Next day, slides were washed and incubated with goat anti-mouse horseradish peroxidase (HRP, 5 µg/ml, P0447, Dako) for 30 min at room temperature. Slides were washed and incubated with rabbit anti-goat HRP (2.5 µg/ml, P0449, Dako) for 30 min at room temperature. Slides were washed and developed using NovaRED following protocol (Vector Labs, Peterborough, UK) and counterstained (Mayer’s hematoxylin, 1.09249.0500, Merck, Darmstadt, Germany) for 25 s. Slides were not counterstained with eosin, which explains why tubules may seem dilated. Slides were dried overnight at room temperature before being covered using entellan (1.07961, Merck).

Different staining techniques were rarely compared directly. Two studies found similar immunofluorescent and immunoperoxidase staining using anti-C5b-9(m) or anti-MAC in various kidney diseases (57, 98). One study mentioned that aE11 did not stain well in paraffin-fixed tissue (28). Direct immunofluorescent staining of C5b-9 was not, in contrast to IgG and C3, affected by acidity, denaturation, or proteolysis (95). Comparisons of staining techniques between included studies were hampered by a paucity of data. Antigen retrieval and blocking, secondary antibodies, antibody concentrations, and detection techniques remained mostly unspecified, yet these techniques determine whether, how intensely, and how selectively staining is perceived. We provide an example of a complete description of staining techniques in the legend of **Figure 2**. We could not discern differences in results of included studies depending on staining techniques (**Supplementary Tables 2**and **3**), except for a possibly higher frequency of tubular deposits based on immunofluorescent as compared with immunoperoxidase staining (**Supplementary Figures 3** and **4**).

Staining of C5b-9 was similar in tissue obtained with autopsy or biopsy in studies on diabetic nephropathy and lupus nephritis (27, 95). In a study on healthy kidneys, it was more often present in tissue obtained with autopsy than biopsy (27), possibly because the latter were healthy living donors. Also in included studies, autopsies might reveal more frequent staining in healthy but not diseased kidneys (**Supplementary Figures 5** and **6**).

### Clearance of C5b-9

**Figure 3:**
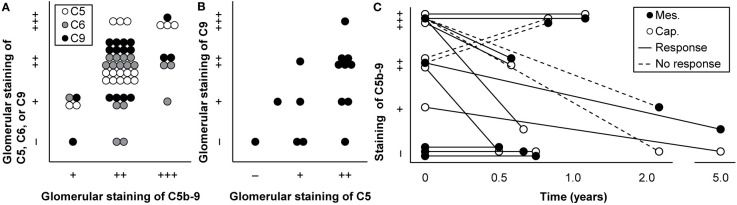
Technical aspects of staining of C5b-9. **(A)** Glomerular staining intensity of C5b-9 is shown in relation with those of its individual components C5, C6, and C9 in kidney biopsies of patients with IgA nephropathy (*n* = 18). Antibody anti-MAC-neo was used for staining of C5b-9. We plotted previously published individual data (52). **(B)** Glomerular staining intensities of C5 and C9 are compared in kidney biopsies of patients with IgA nephropathy (*n* = 15). We plotted previously published individual data (76). **(C)** Staining intensities of C5b-9 in the mesangium (mes.) and along the capillary wall (cap.) are shown for first and repeat biopsies with the time between both biopsies in patients with lupus nephritis (*n* = 8) who responded or did not respond to immunosuppressive treatment. Antibody aE11 was used for staining. We plotted previously published individual data (18).

Membrane-bound C5b-9 is stable and cleared slowy. Indeed, glomerular staining of C5b-9 was equal in patients with active or chronic lupus nephritis, while that of C3 disappeared from the latter (18). It was present in biopsies taken both shorter and longer than twenty weeks after the onset of IgA vasculitis, whereas C3 and MBL were present in only the former (99). It remained present with unchanged intensity in patients with C3 glomerulopathy or thrombotic microangiopathy after one or two weeks (100), after three months (101), after four months (102), and after a year (103) of treatment with eculizumab. Yet, in other reports on various kidney diseases, its staining resolved within three days after administration of eculizumab (104), after three months to 3 years of treatment with eculizumab (105–108), and after half a year of treatment with other immunosuppressive medication (18, 109), as illustrated in **Figure 3C**. Resolution over short periods may reflect active shedding of C5b-9 from cells, initial staining of C5b-9 on extracellular vesicles, initial staining of circulating sC5b-9, or variability of the staining technique; resolution over longer periods may reflect a true effect of complement inhibition.

## Healthy Kidneys

Knowledge on deposition of C5b-9 in healthy kidneys is crucial to understand its relevance in kidney diseases. Tissue from healthy kidneys is, however, generally unavailable for research. Deposition was, consequently, explored infrequently and only in small, ill-defined, and sometimes heterogeneous groups. These groups mostly served as controls in studies on patients with kidney diseases, yet might themselves not always have healthy kidneys. For example, in a rare study providing such details, controls were biopsied because of proteinuria, hematuria, edema, hypertension, or an elevated creatinine up to 522 μmol/l and sometimes had lesions consistent with a mesangioproliferative glomerulonephritis (52). Other sources of tissue included autopsies, biopsies of kidney transplants before, during, or after transplantation, biopsies without histological lesions conducted in most cases because of microscopic hematuria, unaffected parts of kidneys nephrectomized because of a kidney tumor, and unclear sources.

In these presumably healthy kidneys, deposits of C5b-9 were absent (31, 52, 70, 76, 90, 92, 95, 102, 110–125) or sparse and granular in the mesangium (18, 28, 44, 67, 72, 75, 77, 96, 97, 102, 103, 111, 126–133) and vascular pole (18, 44, 126, 132, 134). Deposits were variably reported to be present or absent in the capillary wall (18, 27, 28, 44, 52, 67, 70, 75, 97, 103, 111, 112, 123, 126, 127, 129, 132, 134). Deposits were furthermore reported occasionally along Bowman’s capsule (28, 96, 103, 111, 128, 129) and segmentally and granularly along the tubular basement membrane (18, 28, 46, 67, 70, 72, 75, 83, 94, 96, 97, 103, 118, 127, 129, 135, 136). Deposits were more prominent in the vascular wall (18, 27, 28, 44, 67, 70, 72, 75, 77, 84, 94, 96, 97, 103, 111, 124, 126, 127, 130, 131, 134, 135, 137–139) but absent from peritubular capillaries (83, 130). In the vascular wall, staining covered on average 6% of the media (84). We provide an example of sparse mesangial staining and more prominent vascular staining in a living donor before kidney transplantation—probably the closest representation of a healthy kidney—in **Figure 2A**.

Immunoelectron microscopy revealed C5b-9 granularly along extracellular striated membranous structures—thought to be cell membrane fragments—in the mesangium, glomerular basement membrane, tubular basement membrane, and adjacent to myocytes in the vascular wall but not on cells themselves. This was similar for autopsies (126), nephrectomized kidneys (96), biopsies (72), and kidney tissue of unclear source (44, 97).

Formation and deposition of C5b-9 is physiologically expected to be negligible in healthy kidneys, as confirmed by several studies. Sparse and segmental deposition, as described in other studies and as shown in **Figure 2A**, may be explained by localized cellular injury acquired during aging, due to subclinical or unrecognized kidney disease, or as a result of tissue sampling. This explanation fits observations of deposits of C5b-9 being accompanied by deposits of C1q, C3, C4, or FH in the glomerulus (18, 27, 44, 83, 96, 103, 131) and by deposits of C3, C4, or FH along the tubular basement membrane and in the vascular wall (44, 67, 70, 75, 94, 96, 103, 134, 138). This explanation suggests that deposition of C5b-9 is more likely in tissue obtained from older individuals, in the presence of a kidney tumor, or with autopsy.

In one comparative study, staining of C5b-9 was absent from the kidney of a fetus, sparse in the mesangium and vascular wall in a newborn but stronger in the mesangium and in the vascular wall and additionally appearing along the capillary wall and tubular basement membrane in two adults aged 55 and 65 years (44, 126). In two individuals with unknown ages, glomerular staining was independent of age (137). Although only a limited number of other studies reported ages, glomerular staining seemed more common and more intense in those that included older individuals (**Supplementary Table 2**).

Deposition of C5b-9 might be more frequent in kidney tissues obtained with autopsy than biopsy or nephrectomy, as discussed in the previous section.

Staining of C5b-9 in the vascular wall is recognized as a positive control (19, 134). Staining in the vascular pole of the glomerulus was similarly common (**Figure 2A**). In addition to the explanation above, staining in association with the vasculature may reflect the recently discovered ability of renin to cleave C3 and activate the alternative pathway (101, 140).

Apart from the vasculature, deposition of C5b-9 in presumably healthy kidneys was less common and less intense than in most kidneys diseases, as shown in **Figures 1** and **2** and discussed hereafter.

## Non-Immunological Kidney Diseases

### Minimal Change Nephropathy

In minimal change nephropathy, complement activation is not known to play a pathogenetic role. Complement factors and immunoglobulins are usually absent from the kidney. In line with this, deposition of C5b-9 was similar as in healthy kidneys, being absent from the glomerulus or weakly present as fine granules in the mesangium but not in the capillary wall, and more intense in the vascular wall, predominantly in areas of vascular hyalinosis and sclerosis (57, 61, 67, 70, 72, 75, 79, 81, 85–87, 96, 98, 113, 121, 128, 139, 141–143). Few studies reported slightly more frequent and intense staining in the glomerulus as compared with healthy kidneys (18, 116, 117). One study reported prominent deposits along Bowman’s capsule (79). Deposits were furthermore focally present along the tubular basement membrane, concentrated in areas of tubulointerstitial injury (18, 57, 67, 70, 72, 75, 79, 96, 142, 143). Immunoelectron microscopy revealed that deposits were associated with striated membranous structures or cell remnants in the glomerular basement membrane, mesangium, podocyte foot processes, tubules, and vascular wall (72, 79).

### Glomerular Basement Membrane Diseases

Patients with glomerular basement membrane disease, like Alport’s syndrome, were used as negative controls. They had no or only traces of deposits of C5b-9 or other complement factors in the glomerulus (18, 85–87, 130, 139, 143), except for areas of glomerulosclerosis (96, 143). Reports were inconsistent as to whether they had deposits along the tubular basement membrane and in the vascular wall (18, 96, 130, 143).

### Hypertensive Nephropathy

Hypertension can be regarded as a chronic smoldering inflammatory disease. It is associated—through unclear mechanisms—with activation of complement and formation of C5b-9, which contribute to vascular injury and end-organ dysfunction in animal models (40).

Glomerular deposits of C5b-9 were more common and extensive in patients with hypertensive nephropathy than in young women with hypertension or healthy individuals (44, 131), while deposits of C3 were absent (44, 67, 144). C5b-9 was found extensively in the mesangium, including the juxtaglomerular region, in a coarse granular pattern along Bowman’s capsule but not or only focally along the capillary wall (44, 67) and sometimes along the tubular basement membrane (44, 67, 95, 143). It was predominant in glomerular and vascular areas of expansion, sclerosis, and hyalinization (44, 67, 143). Vascular staining was moderately intense and covered 10% of the arterial media, similar to hypertension without nephropathy but more intense and extensive than in healthy kidneys (44, 67, 84). The extent of staining in the media correlated with loss of smooth muscle cells in hypertension with or without nephropathy (*r* = 0.82 and *r* = 0.79, respectively) (84).

### Preeclampsia

Preeclampsia, characterized by hypertension and proteinuria in pregnancy, is partly attributable to activation of complement in the placenta and along the endothelium elsewhere. It is associated with elevated levels of C5a and sC5b-9 in blood and urine, which explains why treatment with eculizumab has beneficial effects (32). The only study on deposits of C5b-9 found them rarely and segmentally in the glomerulus, mostly in areas of glomerulosclerosis. Other localizations were not evaluated (131).

### Diabetic Nephropathy

Chronic hyperglycemia leads to glycation of proteins, referred to as advanced glycation end-products. These proteins may expose neoepitopes that are recognized by C1q and MBL, which activate the classical and lectin pathways. Glycation of factors that normally inhibit complement activation, like CD59, may enhance complement activation or directly induce formation of C5b-9. As a result, sC5b-9 circulates at higher levels in diabetes, is excreted in urine in diabetic nephropathy, and deposits in various organs affected by diabetes (27, 28, 30, 113, 145).

**Figure 4:**
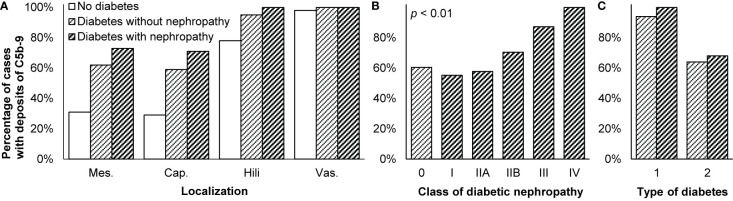
Deposits of C5b-9 in diabetic nephropathy. **(A)** Presence of C5b-9 in the mesangium (mes.), along the glomerular capillary wall (cap.), in glomerular hili, and in the extraglomerular vascular wall (vas.) is compared between patients without diabetes or kidney disease (*n* = 41), patients with diabetes who had no nephropathy (*n* = 58), and patients with diabetic nephropathy (*n* = 101). **(B)** Presence of C5b-9 in the glomerulus is compared between patients with different classes of diabetic nephropathy (*n* = 101) according to the classification of the Renal Pathology Society (146). Patients with diabetes but without diabetic nephropathy (*n* = 58) are indicated as class 0. Differences between classes were tested with Spearman’s correlation. We reproduced both panels without adaptations from their previous publication under the CC BY-NC-ND license (27), ^©^ International Society of Nephrology. **(C)** Presence of C5b-9 in the glomerulus is compared between patients with diabetes type 1 (*n* = 17) and type 2 (*n* = 120). It was different between diabetes types 1 and 2, both among patients without and with diabetic nephropathy, as tested with Fisher’s exact test (both *p* < 0.05). The antibody used for staining in these three panels was unspecified. We plotted previously published data (27).

Glomerular deposits of C5b-9 were more common in patients with diabetic nephropathy than in healthy individuals (27, 28, 44, 72, 96, 113, 126, 139, 143). Deposits were found ubiquitously and granularly in the mesangium, coarsly along Bowman’s capsule, and focally along the capillary wall (28, 44, 67, 70, 96, 126), although more along the capillary wall than in the mesangium in one study (113). Deposits were coarsely present along the tubular basement membrane with MBL and MASPs (28, 44, 67, 70, 72, 96, 126, 142) and in the vascular wall (28, 44, 67, 70, 72, 84, 96, 126), also with higher staining intensity than in healthy kidneys (28, 84). Intense staining in the glomerulus and vascular wall was likewise observed in a case of recurrent diabetic nephropathy after transplantation (113). Deposits were most extensive in glomerular, tubular, and vascular areas of expansion, sclerosis, hyalinization, and amyloidosis (28, 44, 67, 70, 72, 96, 126, 143) but absent from crescents (72, 96). Glomerular and vascular deposits were only slightly more frequent in diabetic nephropathy than in diabetes without kidney disease (27), as reproduced in **Figure 4A**.

Immunoelectron microscopy revealed that C5b-9 colocalized with cell membrane fragments in areas of glomerulosclerosis, in the glomerular basement membrane, tubular basement membrane, and vascular wall but not bound to epithelial, mesangial, or tubular cells (96, 126).

#### Histological Correlates

As reproduced in **Figure 4B**, glomerular deposits of C5b-9 were increasingly common in more severe cases of diabetic nephropathy (27). The extent to which staining covered the arterial media likewise increased from 10% in mild to 28% in severe diabetic nephropathy (84).

Staining intensity of C5b-9 was reported to correlate with the severity of histological lesions. In the glomerulus, it correlated with the degree of mesangial expansion; in both the glomerulus and tubules, it correlated with the degree of tubular injury and atrophy (27, 28, 96, 143). In the tubules and interstitium combined, it correlated with the number of interstitial infiltrating cells (*ρ* = 0.53, *p* < 0.01), interstitial volume (*ρ* = 0.56, *p* < 0.01), and the degree of tubular and interstitial inflammation and injury (*ρ* = 0.52, *p* < 0.01) (28). In the vascular wall, C5b-9 colocalized with glycated CD59 (113) and other advanced glycation end-products and apoptotic smooth muscle cells (84).

#### Clinical Correlates

Staining intensity of C5b-9 throughout the kidney was higher in patients with higher creatinine and more albuminuria. Staining intensity in the tubules and interstitium combined correlated weakly with levels of urine markers reflecting tubular injury. Staining did not correlate with the plasma level of sC5b-9 (28). One study found glomerular deposits more often in patients with diabetes type 1 than type 2, as shown in **Figure 4C**, possibly due to a longer disease duration (27).

## Kidney Diseases Due to Immune Complex Deposition

### Primary Membranous Nephropathy

Primary—formerly idiopathic—membranous nephropathy is caused by autoantibodies that bind antigens expressed by podocytes, in most cases PLA2R. These autoantibodies are predominantly of the IgG4 class, which cannot bind C1q and thus cannot activate the classical pathway. Rather, the lectin and alternative pathways are activated, given that C3, C4, FH, FB, and MBL, but not C1q, affect the risk of membranous nephropathy and are generally present in the subepithelial immune deposits. However, the pathways may be variably activated due to variation in the characteristics of the autoantibodies and their antigens, even during the disease’s course. Autoantibodies of the IgG1 class directed against exostosin or neutral endopeptidase activate the classical pathway (24, 25).

Formation of C5b-9 is regarded essential for the development of kidney injury and proteinuria (24, 25). It disrupts proteins of organelles, the cytoskeleton, and slit diaphragm of podocytes. The urine level of sC5b-9—probably shed by podocytes—correlates with disease activity. In animal models, deficiency or inhibition of C5, C6, or C8 prevents deposition of C5b-9 and proteinuria (24, 25, 79, 123).

In line with this, staining of C5b-9 was more intense and extensive in membranous nephropathy than in healthy kidneys (18, 44, 67, 72, 75, 82, 96, 123, 139), also when recurring in a transplant (147). It was intense in the glomerulus (57, 81, 82, 139, 143, 148–151), always along the capillary wall, but not or hardly in the mesangium (18, 44, 67, 72, 75, 77, 79, 87, 98, 112, 123, 141, 147, 152–155), in a granular (77, 82, 112, 154), linear (123), or mixed pattern (79). It was furthermore focally found along Bowman’s capsule (79, 152), as coarse granules along the tubular basement membrane (18, 44, 57, 67, 70, 72, 75, 79, 96, 112, 142), occasionally on tubular cells (79, 112), in the vascular wall (18, 57, 67, 72, 96, 112), in capsular adhesions, crescents, and glomerular and vascular areas of hyalinosis and sclerosis (44, 57, 67, 70, 72, 77, 96, 143). The extent of tubular staining varied widely between 10 and 88% (112). Not all studies specified included cases as specifically primary membranous nephropathy.

Immunoelectron microscopy revealed that C5b-9 was associated with striated membranous structures in immune deposits, basal membranes of adjacent podocyte foot processes, the glomerular basement membrane, and the mesangium, more often so in stage IV than I or II (72, 79, 152).

#### Histological Correlates

**Figure 5:**
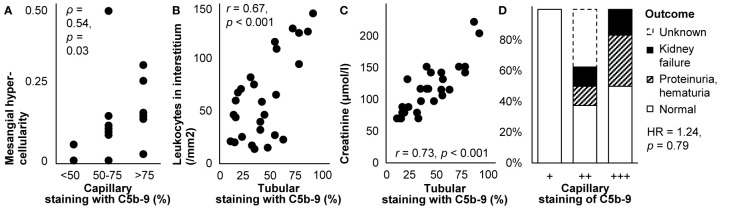
Deposits of C5b-9 in primary membranous nephropathy: examples of correlations with histological lesions and clinical characteristics. **(A)** The extent of staining of C5b-9 in the capillary wall is shown in relation with mesangial hypercellularity scored on scale from 0–3 in children with idiopathic membranous nephropathy (*n* = 16). The antibody used for staining was unspecified. Relations were tested with Spearman’s correlation (*ρ*). We plotted previously published individual data (87). **(B)** The extent of staining of C5b-9 in tubules is shown in relation with the number of leukocytes in the interstitium in patients with idiopathic membranous nephropathy (*n* = 27). **(C)** The extent of staining of C5b-9 in tubules is shown in relation with serum creatinine at the time of biopsy in patients with idiopathic membranous nephropathy (*n* = 27). Antibody aE11 was used for staining in both panels. Relations were tested with Pearson’s correlation (*r*). We reproduced both panels without adaptations from their previous publication with permission (112), ^©^ European Renal Association–European Dialysis and Transplant Association. **(D)** The extent of staining of C5b-9 in the capillary wall is shown in relation with the clinical outcome after 14-171 months of treatment in children with idiopathic membranous nephropathy (*n* = 16). The antibody used for staining was unspecified. The hazard ratio (HR) for kidney failure, proteinuria, or hematuria as compared with normal outcomes is given as estimated with Cox’s regression. We plotted previously published individual data (87).

C5b-9 colocalized with immune deposits containing IgG, C3, and sometimes C1q and C4 in the capillary wall in all stages of primary membranous nephropathy (44, 67, 70, 72, 75, 77, 81, 82, 87, 96, 112, 139, 143, 147–149, 152, 154–156), except for stage I according to one report (72). It colocalized with causative autoantibodies in subepithelial immune deposits (150, 155, 156). By contrast, it was absent from the glomerulus where its inhibitors clusterin and CD59 were present (80, 82). Staining along the capillary wall correlated with mesangial hypercellularity (87), as illustrated in **Figure 5A**. Staining was more frequent in glomeruli with than without capsular adhesions (83 vs. 17%) (77). The extent of glomerular staining correlated with neither the stage of disease nor the extent of tubular staining (112). Tubular staining was concentrated in areas of interstitial inflammation and fibrosis and tubular atrophy (57, 70, 72, 96, 112), as reproduced in **Figure 5B**.

#### Clinical Correlates

Glomerular staining intensity correlated with the amount of proteinuria (57); patients with glomerular staining had more proteinuria than those without (3.6 vs. 2.3 g/d) (77). The extent of tubular staining correlated with creatinine (112), as reproduced in **Figure 5C**. Glomerular and tubular staining intensities of C5b-9 did not correlate with blood pressure, the nephrotic syndrome, hematuria, or serum levels of C4 or C3 (87, 112).

As illustrated in **Figure 5D**, glomerular staining intensity seemed associated with the outcome during treatment among children (87). In a case of lupus-like membranous nephropathy, however, staining remained unchanged despite decreased proteinuria after 40 weeks of treatment with intraveneus immunoglobulins (149).

### Secondary Membranous Nephropathy

Secondary membranous nephropathy is caused by autoantibodies that circulate due to infections, autoimmune diseases, malignancies, or medication. They deposit in the subepithelial and often also the subendothelial space and activate the classical or lectin pathway (24, 25). Only few studies reported on deposition of C5b-9 in secondary membranous nephropathy. It was present in immune deposits in medication-induced membranous nephropathy stages II and III but not I (96, 157). It was similarly found in immune deposits along the capillary wall in membranous nephropathy due to hepatitis B (152)—although not in all cases (77)—where it colocalized with HBe and sometimes HBs (152).

### IgA Nephropathy

In IgA nephropathy, galactose-deficient IgA due to aberrant glycosylation is bound by autoantibodies and deposits as immune complexes in the mesangium. There, it causes mesangial expansion and inflammation with widely varying histological and clinical presentations (15, 17).

Circulating and deposited IgA–containing immune complexes can activate the alternative and lectin pathways but not the classical pathway. C3, FH, and properdin of the alternative pathway and sometimes C4d, MBL, and MASPs of the lectin pathway deposit in the mesangium too. C1q of the classical pathway is only infrequently present. Whether only the alternative or also the lectin pathway is activated probably varies between patients (15, 17, 158). As their end-product, the urine level of sC5b-9 is elevated and associates with disease severity. Inhibiting the formation of C5b-9 with eculizumab has inconsistent beneficial effects in patients (15, 17, 21, 158, 159).

In small comparative studies, all or almost all patients with IgA nephropathy had deposits of C5b-9 in the glomerulus that were more intense, more diffuse, and more coarse than in healthy individuals (44, 52, 67, 72, 75, 81, 96, 97, 124, 128, 139). All individual components of C5b-9 were two to four times more abundant in the glomerulus in patients with stable IgA nephropathy than in healthy individuals as quantified with mass spectrometry (124).

Mostly small descriptive studies found C5b-9 as coarse granules in the glomerulus (57, 58, 72, 76, 81, 137, 139, 143, 158, 160, 161)—always in the mesangium, sometimes also along the capillary wall (19, 44, 52, 67, 75, 96, 97, 110, 115, 124, 128, 162–166), in one case report only along the capillary wall (159) —, along Bowman’s capsule (52, 162, 163), as coarse granules (19, 44, 52, 57, 70, 72, 75, 96, 97, 110, 115, 163) or linearly (110, 115) along the tubular basement membrane and occasionally on tubular cells (110), and in the vascular wall (19, 52, 57, 67, 72, 96, 110, 115, 137, 162). Deposits along the capillary wall were localized in the subepithelial space (97, 164). The extent of staining in tubules varied widely between 19 and 87% (110, 115). Deposits were furthermore present in areas of mesangial expansion and in glomerular and vascular areas of amyloidosis, hyalinosis, and sclerosis (19, 44, 57, 67, 72, 75, 96, 97, 143) but not in crescents (19, 72, 96, 97). Among patients with IgA nephropathy or IgA vasculitis with nephritis together, deposits were less frequent in the mesangium and vascular wall (19, 167). One case of IgA nephropathy with thrombotic microangiopathy exhibited no deposits (130).

Immunoelectron microscopy revealed deposits of C5b-9 in various patterns: as homogeneous fine granules along the glomerular basement membrane in the paramesangial region, as rings or ribbons associated with striated membranous structures or cell remnants in the glomerular basement membrane, subepithelial space, mesangium, tubular basement membrane, and vascular wall, and as patches in electron-dense deposits in the mesangium (72, 97).

#### Histological Correlates

Glomerular deposits of C5b-9 colocalized with IgA and C3–containing immune complexes (44, 52, 57, 67, 70, 72, 75, 76, 81, 96, 97, 110, 128, 137, 143, 158, 160, 161, 165). Their staining was less intense than that of IgA (52, 76, 137). The localization and intensity of their staining correlated with those of C3 mRNA expression (128).

**Figure 6:**
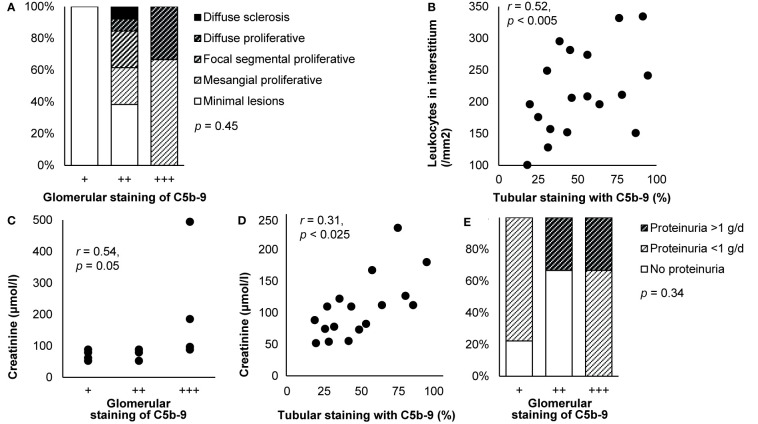
Deposits of C5b-9 in IgA nephropathy: examples of correlations with histological lesions and clinical characteristics. **(A)** Staining intensity of C5b-9 in the glomerulus is shown in relation with histological patterns in patients with IgA nephropathy (*n* = 18). Antibody anti-MAC-neo was used for staining. Differences between staining intensities were tested with Fisher’s exact test. We plotted previously published individual data (52). **(B)** The extent of staining of C5b-9 in tubules is shown in relation with the number of interstitial monocytes and macrophages in patients with IgA nephropathy (*n* = 18). Antibody aE11 was used for staining. We reproduced this panel without adaptations from its previous publication with permission (110), ^©^ European Renal Association–European Dialysis and Transplant Association. **(C)** Staining intensity of C5b-9 in the glomerulus is shown in relation with serum creatinine at the time of biopsy in patients with IgA nephropathy (*n* = 14). Antibody aE11 was used for staining. We plotted previously published individual data (128). **(D)** The extent of staining of C5b-9 in tubules is shown in relation with serum creatinine at the time of biopsy in patients with IgA nephropathy (*n* = 18). Antibody aE11 was used for staining. We reproduced this panel without adaptations from its previous publication with permission (110), ^©^ European Renal Association–European Dialysis and Transplant Association. Relations were tested with Pearson’s correlation (*r*). **(E)** Staining intensity of C5b-9 in the glomerulus is shown in relation with proteinuria at the time of biopsy in patients with IgA nephropathy (*n* = 18). Antibody anti-MAC-neo was used for staining. Differences between staining intensities were tested with Fisher’s exact test. The relatively small number of patients may explain why proteinuria <1 g/d was not observed in the group with a staining intensity of ++. We plotted previously published individual data (52).

Various studies reported a relation between staining of C5b-9 and histological lesions. Glomerular staining intensity correlated with the extents of mesangial expansion and hypercellularity, glomerulosclerosis, interstitial inflammation, interstitial fibrosis, and tubular atrophy (57, 115, 128, 137, 139, 163). It also seemed correlated with the extent of proliferative glomerulonephritis (52), as illustrated in **Figure 6A**. Individual components of C5b-9 were two times more abundant in patients with than without global mesangial hypercellularity, endocapillary hypercellularity, or moderate to extensive interstitial fibrosis or tubular atrophy but equally abundant in patients with or without glomerulosclerosis (124). Glomerular deposits were more frequent when capsular adhesion and crescents were present (76), while those with cellular or fibrocellular crescents had more intense staining (166). Deposits seemed also more frequent in the glomerulus (27 vs. 12%, *p* = 0.06) and vascular wall (68 vs. 46%, *p* = 0.06) when thrombotic microangiopathy was present (167). Global glomerular staining was associated with loss of podocytes (*r* ^2^ = 0.18, *p* < 0.05), perhaps due to their lower expression of CR1 (*r* ^2^ = 0.45, *p* < 0.05), which antagonizes complement activation (164). Tubular staining intensity correlated with the extents of interstitial inflammation and fibrosis and tubular atrophy (57, 72, 96, 110, 115), as reproduced in **Figure 6B**.

Although the aforementioned studies included children, two studies including only children did not find any correlation between glomerular or tubular staining and histological lesions (19, 97).

#### Clinical Correlates

Various studies also reported a relation between staining of C5b-9 and clinical outcomes. Glomerular and tubular staining intensities of C5b-9 correlated with creatinine (110, 115, 128), as shown in **Figures 6C, D**. They were also higher in patients with heavy proteinuria or the nephrotic syndrome (19, 52, 163), as illustrated in **Figure 6E**, although these correlations did not hold in sensitivity analyses (19). Amounts of its individual components in microdissected glomeruli were higher when blood pressure was higher or when eGFR was lower but not related to proteinuria (124). A correlation between glomerular staining intensity and age was reported without further details (137). Otherwise, staining was not related with age, sex, hematuria, serum levels of immunoglobulins, C3, or C4, or disease duration (19, 52, 76, 97, 110, 128, 137).

Glomerular deposits of C5b-9 were more often present (41 vs. 89%, unadjusted odds ratio 12, *p* = 0.004) and stained more intensely in progressive as compared with stable IgA nephropathy (158). Amounts of C5 through C9 were about twice as high in the former as compared with the latter, which was among the largest difference of all studied proteins (124).

Among children with IgA nephropathy or IgA vasculitis with nephritis, those with deposits of C5b-9 in the glomerulus or tubules received immunosuppressive medication more often than those without deposits (89 vs. 62%, *p* = 0.02) and had, probably as a result, a shorter time to recovery (unadjusted hazard rate 0.17, *p* = 0.02) (19). In adults who had C5b-9 in more than half of the tubules, creatinine increased from 150 to 248 µmol/l during a mean follow-up of 30 months, while it remained stable around 88 µmol/l in those who had less tubular deposits (110). An undefined relation between glomerular and tubular staining intensities and creatinine after a longer follow-up was reported too (115).

#### IgA Nephropathy With Complement Factor Deficiency

Mild forms of IgA nephropathy were reported in patients with complement factor deficiencies limiting formation of C5b-9, in whom disease could arise from sublytic effects of incomplete C5b-9. Two children with a congenital C9 deficiency developed IgA nephropathy with mesangial deposits of C3, C5, and C8 but not C9 or C5b-9. Their histological lesions were only mild, eGFR remained normal, and proteinuria resolved spontaneously (78). An adolescent with IgA nephropathy and homozygous C3 deficiency exhibited weak mesangial staining of C5b-9 together with immunoglobulins, C1q, C4, and properdin but not C3. He too had only mild histological abnormalities (93). An adult man with C9 deficiency suffered from progressive IgA nephropathy without deposition of C5b-9 (151).

### IgA Vasculitis With Nephritis

IgA vasculitis—or Henoch-Schönlein purpura—can present with a nephritis that closely resembles IgA nephropathy, so that some regard it as a systemic form of IgA nephropathy. Activation of the alternative and lectin pathways are similarly thought to underlie the nephritis (168, 169). In the few studies conducted specifically on patients who had IgA vasculitis with nephritis, deposits of C5b-9 were present in the mesangium and capillary wall, colocalized with IgA and C3–containing immune complexes (52, 61, 75, 85, 99, 139), along the tubular basement membrane, and in the vascular wall (75, 96). Mesangial deposits of C5b-9 were less common in patients with mesangial deposits of IgA1 only, in whom the alternative pathway was activated, than in those with deposits of both IgA1 and IgA2, in whom the lectin pathway was also activated (73 vs. 100%). Four patients without deposits of C5b-9 had less intense staining of IgA and C3 but paradoxically more proteinuria than 27 with deposits (median 210 vs. 80 mg/dl) (85). Deposits of C5b-9 were not different between children with IgA vasculitis or IgA nephropathy but were less clearly associated with prognosis in the former (19).

### Lupus Nephritis

Autoantibodies that circulate in SLE give rise to lupus nephritis when they form or deposit as immune complexes in the glomerulus. They activate the classical pathway, reflected in most patients by specific full-house deposition of IgG, IgA, IgM, C1q, and C3. Activation of the alternative pathway, seems essential too, given that more C3 than C4 deposits, that deficiencies of factors of the alternative pathway, like FB and FD, ameliorate lupus nephritis, and that deficiencies of its inhibitory factors, like FH, promote lupus nephritis in animal models (10, 11).

Formation of C5b-9 may be both a cause and consequence of deposition of immune complexes and cellular injury (11, 119). Levels of sC5b-9 are elevated in blood and urine of patients and correlate with disease activity. Pointing to a causative role, inhibition of C5 reduces histological lesions, proteinuria, and mortality in animal models, while eculizumab exerts beneficial effects in patients (10, 11).

In line with such a role, glomerular and tubular deposits of C5b-9 were more common in patients with lupus nephritis than healthy individuals (18, 44, 46, 67, 72, 75, 81, 96, 111, 119, 133, 139). Descriptive studies on mostly small numbers of patients reported ubiquitous deposits in the glomerulus (18, 46, 58, 72, 77, 81, 95, 96, 111, 119, 133, 139, 143, 170, 171)—both in the mesangium and along the capillary wall (18, 44, 57, 67, 75, 111, 172–174) and sometimes along Bowman’s capsule (119, 171) —, linearly or granularly along the tubular basement membrane (18, 44, 57, 67, 70, 72, 75, 95, 96, 119, 142, 151, 171), and in the vascular wall (18, 57, 67, 72, 95, 96, 119, 171). Deposits were also present in glomerular and vascular areas of hyalinization, sclerosis, and fibrinoid necrosis (44, 57, 67, 70, 95, 96, 143) but not in crescents (72, 96, 171).

Deposits of C5b-9 were mainly located in the mesangium in lupus nephritis class II, III, or IV and granularly along the subepithelial side of the capillary wall in class V, although mesangial deposits often extended into the capillary walls and vice versa (18, 77, 95, 111, 152, 172). They colocalized with immune deposits containing immunoglobulins and other complement factors in all classes (44, 46, 57, 67, 70, 72, 75, 77, 81, 95, 96, 143, 152, 172, 173). Glomerular, but not tubular, staining of C5b-9 was more intense in more severe classes, increasing from I and II to III and V and being strongest in class IV (18, 95, 111, 119, 139, 171, 174). In lupus nephritis with thrombotic microangiopathy, staining intensity was variable in the glomerulus and strong in the vascular wall (100, 130, 175).

Immunoelectron microscopy revealed that C5b-9 was associated with immune deposits, striated membranous structures, and partly shedded cell membrane extensions or with cell membrane fragments in the mesangium, the capillary wall, and glomerular basement membrane without signs of cellular lysis (95, 96, 152, 172). Some cell membrane fragments in the glomerular basement membrane appeared to be infolding degraded parts of podocytes (170, 172). C5b-9 was furthermore associated with structural defects of the tubular basement membrane (95).

#### Histological Correlates

**Figure 7:**
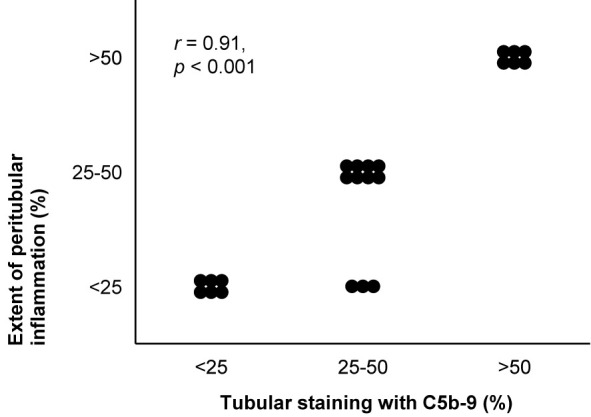
Deposits of C5b-9 in lupus nephritis: examples of correlations with histological lesions and clinical characteristics. The extent of staining of C5b-9 in tubules is shown in relation with the extent of peritubular inflammation in patients with lupus nephritis class II, III, IV, or V (*n* = 22). The antibody indicated as Kolb 1975 in **Table 2** was used for staining. The relation was tested with Pearson’s correlation (*r*). We reproduced this panel without adaptations from its previous publication with permission (95), ^©^ Rockefeller University Press.

Staining intensity of C5b-9 correlated with those of immunoglobulins and C3 (70) and with loss of podocytic expression of CR1 (111). Glomerular staining intensity of C5b-9 did not consistently correlate with histological signs of active or chronic nephritis. In a small study, it correlated with the activity index (111), but in other studies it rather correlated with the chronicity index, although weakly (174), or with neither index (18, 171). It did not correlate with the number of macrophages in the glomerulus (18). Tubular staining colocalized with interstitial inflammation (70, 95) and correlated with interstitial fibrosis (18, 57, 72, 96), as reproduced in **Figure 7**. The extents of glomerular and tubular staining of C5b-9 did not correlate mutually (95, 171).

#### Clinical Correlates

Correlations between deposits of C5b-9 and clinical characteristics were studied little. Patients with lupus nephritis class V and other types of membranous nephropathy had more proteinuria if they had deposits in the capillary wall (3.6 vs. 2.3 g/l, *p* < 0.02) (77). Patients with various classes of lupus nephritis were more often men (39 vs. 6%, *p* = 0.06), had higher blood pressure (133/82 vs. 117/70 mmHg, *p* < 0.03) and seemed more frequently to have low serum levels of C3 (92 vs. 65%, *p* = 0.10) (171) and C4 (57) if they had deposits in the glomerulus. There were no correlations with age, race, symptoms of SLE, medication, creatinine, hematuria, hemoglobin, albumin, or serum level of anti-dsDNA autoantibodies (18, 77, 171).

Glomerular deposits of C5b-9 seemed to predict treatment effect: patients with deposits responded less often, with an unadjusted odds ratio of 0.60 (*p* = 0.58) for any response after a year of treatment (18) and a multivariate-adjusted odds ratio of 0.22 for any response after six months of treatment (171). Their staining intensity seemed to correlate with treatment effect too, although the change in intensity in biopsies repeated after treatment did not (18), as illustrated in **Figure 3C**. In a case of recurrent lupus nephritis class II, mesangial staining was similar as in a first biopsy taken 5 years earlier, while staining of immunoglobulins and other complement factors had increased (170).

#### Lectin Pathway

The lectin pathway has recently been suspected to contribute to the pathogenesis of lupus nephritis. Polymorphisms of MBL increase the risk of lupus, its circulating level is high in patients with lupus nephritis, and it frequently deposits in their kidneys (18, 24, 176). Glomerular deposits of C5b-9 and MBL concurred in 82% and their staining intensities correlated well in eleven patients with lupus nephritis. C5b-9 and MBL were also deposited in Bowman’s capsule, tubules, and the vascular wall (119).

### Membranoproliferative Glomerulonephritis

Immune complex-mediated membranoproliferative glomerulonephritis is regarded a disease of an activated classical pathway, elicited by deposition of immunoglobulins and subsequently leading to codeposition of complement factors. Deposits of C5b-9 were present with immune complexes along the capillary wall (108), although C5 through C9 were only rarely detected with mass spectrometry of microdissected glomeruli (90). In two teenagers treated with eculizumab, the extent of glomerular staining decreased slightly and histological inflammation improved, but GFR and proteinuria improved in only one of both. With similar clinical characteristics and serum complement levels, the successfully treated case differed only by exhibiting histological thrombotic microangiopathy (108).

## Kidney Diseases Due to Alternative Pathway Activation

### C3 Glomerulopathy

C3 glomerulopathy is regarded a disease of an activated alternative pathway, characterized by deposition of C3 but no or scarce deposition of immunoglobulins or other complement factors. Before this pathogenetic distinction, C3 glomerulopathy and immune complex-mediated membranoproliferative glomerulonephritis were together classified into membranoproliferative glomerulonephritis types I, II, and III according to the localization of immune deposits. An essential role of C5 has been demonstrated in animal models of membranoproliferative glomerulonephritis and C3 glomerulopathy, but rather through effects of C5a on its receptor than formation of C5b-9. Deficiency or inhibition of C5 or the C5a receptor, reduces histological lesions, creatinine, proteinuria, and mortality, whereas deficiency of C6—preventing deposition of C5b-9—does not (177, 178). Correspondingly, inhibition of C5 with eculizumab has beneficial effects in only a subset of patients (4, 5, 7–9).

C3 glomerulopathy is subdivided into C3 glomerulonephritis and dense deposit disease according to the microscopic appearance of electron-dense immune deposits in the glomerular basement membrane (4, 5). As a possible difference in pathogenesis, formation of C5b-9 is presumed to be more pronounced in C3 glomerulonephritis than dense deposit disease (4–6). Individual components of C5b-9 were indeed more abundant in microdissected glomeruli in the former when quantified with mass spectrometry (5, 91, 92), although immunofluorescence staining of C5b-9 was similar in both disease subtypes (103). Staining intensity in both was higher than in healthy kidneys (103) and correlated with those of C3 and FHR5 (102).

In C3 glomerulonephritis, C5b-9 was found in the mesangium, along the capillary wall, Bowman’s capsule, most of the tubular basement membrane, and in the vascular wall (102, 103, 107, 179, 180). Serial biopsies revealed that glomerular staining of C5b-9 and other complement factors increased as the disease progressed (102, 106, 107), regressed during three months to 3 years of treatment with eculizumab along with histological and clinical improvement in three patients (106, 107) but remained unchanged during four months to a year of treatment with eculizumab despite varying histological and clinical responses in three other patients (102, 103).

In dense deposit disease, staining of C5b-9 was intense in the glomerulus (57, 75, 101, 103, 105, 181), similarly when recurring after kidney transplantation (102, 182). They surrounded immune deposits in the mesangium, along the capillary wall, and diffusely along the tubular basement membrane and additionally formed granules along the interstitial side of the tubular basement membrane (44, 103, 126, 181). Treatment with eculizumab resulted in disappearance of their staining after 13 to 18 months in two patients, but unaltered staining after three months to a year in three other patients, with histological and clinical improvement in all five (101, 103, 105, 106).

In a study on patients with C3 glomerulonephritis or dense deposit disease together, median eGFR was 15 ml/min/1.73 m^2^ lower (*p* = 0.03) if glomerular staining of C5b-9 was maximally intense than less intense (102).

Deposition of C5b-9 was reported to be similar in membranoproliferative glomerulonephritis types I, II, and III (75). In membranoproliferative glomerulonephritis type I, deposits of C5b-9 were practically always present in the glomerulus—both in the mesangium and capillary wall similarly to immune deposits —, frequently along the tubular basement membrane (44, 57, 67, 72, 75, 96, 114, 139, 143), and in the vascular wall (57, 67, 72, 96) with variable but higher staining intensity than in healthy kidneys. They surrounded immune deposits in the mesangium, along the capillary wall, and along the tubular basement membrane (44, 72, 96). Immunoelectron microscopy revealed that they were also associated with striated membranous structures in extracellular matrix and with partly shedded cell membrane extensions of mesangial, endothelial, and epithelial cells without signs of cellular lysis (96). Glomerular, tubular, and vascular deposits were concentrated in areas of sclerosis (44, 57, 67, 72, 96). Glomerular staining intensity correlated with the serum level of sC5b-9 (114). In two children with unspecified types of membranoproliferative glomerulonephritis, of whom only one had deposits of C5b-9 in the glomerulus and along the tubular basement membrane, frequent relapses despite treatment occurred in the one with deposits, whereas the one without deposits reached complete remission after seven months (98, 141).

### Postinfectious Glomerulonephritis

Postinfectious glomerulonephritis is often clinically indistinguishable from C3 glomerulopathy and may be regarded an acute variant of a similar pathogenesis (4, 5). Deposits of C5b-9 were found along with immune deposits in the mesangium, along the capillary wall, the tubular basement membrane, and in the vascular wall with higher intensities than in healthy kidneys (67, 86, 143, 183, 184). Staining was restricted to the capillary wall in cases biopsied two weeks after the disease’s onset but increasingly extended into the mesangium after three weeks (183). Glomerular staining intensity was not correlated with age, disease duration, blood pressure, creatinine, proteinuria, hematuria, endocapillary hypercellularity, or crescents, but the number of subepithelial hump-like immune deposits—considered characteristic of postinfectious glomerulonephritis—was higher when staining was intenser (median 0.2 vs. 0.5 per glomerulus, *p* = 0.002) (86).

### Thrombotic Microangiopathy

aHUS is a thrombotic microangiopathy caused by genetic mutations or autoantibodies that activate the alternative pathway, eventually leading to formation of C5b-9 on endothelial cells. In animal models of aHUS, deficiency or inhibition of C5 reduces the thrombotic microangiopathy and histological lesions, creatinine, kidney failure, and mortality. Contrary to C3 glomerulopathy, these effects are brought about through formation of C5b-9 rather than C5a. Deficiency of C6 or C9—preventing deposition of C5b-9—has similar effects as deficiency or inhibition of C5, whereas deficiency of the C5a receptor does not (185, 186). In patients, inhibition of C5 with eculizumab has become standard treatment (12, 187). Regarded a typical finding (12), intense staining of C5b-9 was present in almost all biopsies, in the mesangium, along the capillary wall, along the tubular basement membrane, and predominantly in the vascular wall (75, 103, 130, 188) but not in peritubular capillaries (130). An example is shown in **Figure 2B**. In a late-stage case, staining was weak in the mesangium, absent from the capillary wall, and intense in the vascular wall (100). Staining in recurrent aHUS after transplantation was similar to that in native kidneys (130). Despite its beneficial effects, staining of C5b-9 remained unchanged after treatment with eculizumab (103).

In STEC-HUS, the alternative pathway is activated by direct and indirect effects of the Shiga toxin (189). Although deposition of C5b-9 was found granularly along the capillary wall, in the vascular pole, and in the vascular wall of peritubular capillaries in a child (122) and diffusely in the glomerulus in an adult (190), it was not found in the kidney in eleven other adult patients (130, 153). In line with this, treatment with eculizumab has only exerted beneficial effects in a few children (122, 153).

The alternative pathway is also activated in TTP (120). Deposition of C5b-9 was found along the capillary wall in few glomeruli, in few tubules, in the vascular wall but not in peritubular capillaries, without clear clinical or histological correlates (120, 132).

Thrombotic microangiopathy after hematopoietic stem cell transplantation is characterized by variable complement activation (191). C5b-9 stained moderately in the mesangium and capillary wall in one case, weakly in only the mesangium in another case, and strongly in the vascular wall in both cases. Similar staining was found before and after treatment with eculizumab in one of them (100).

**Figure 8:**
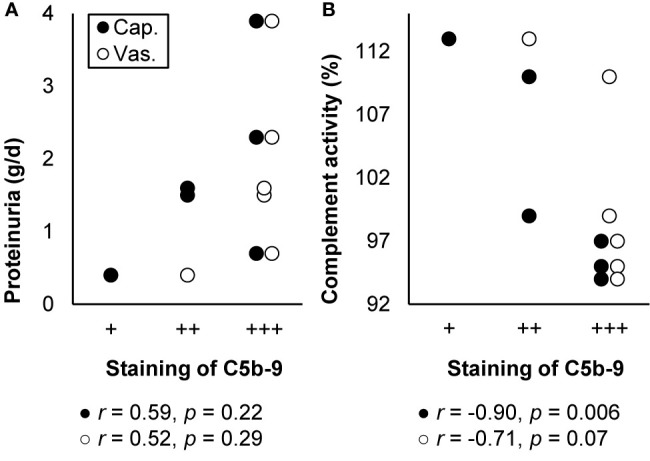
Deposits of C5b-9 in hypertension-associated thrombotic microangiopathy: examples of correlations with histological lesions and clinical characteristics. Staining intensity of C5b-9 along the glomerular capillary wall (cap.) and in the extraglomerular vascular wall (vas.) is shown in relation with **(A)** proteinuria and **(B)** plasma complement activity (CH50) at the time of biopsy in patients with hypertension-associated thrombotic microangiopathy (*n* = 6). The antibody used for staining was unspecified. Relations were tested with Pearson’s correlation (*r*). We plotted previously published individual data (192).

Thrombotic microangiopathy elicited by hypertension has been postulated as often attributable to genetic mutations or autoantibodies that activate the alternative pathway. Supporting this postulation, C5b-9 was often deposited together with C3 and C4d along the capillary wall, segmentally in the vascular pole, and always in the vascular wall in patients with hypertension-associated thrombotic microangiopathy. Staining was intense, though weaker in recurrent cases after transplantation. Staining intensity did not correlate with age, sex, blood pressure, the plasma level of sC5b-9, or disease severity but seemed to correlate with proteinuria and correlated with complement activity, as illustrated in **Figure 8**. Treatment with eculizumab prevented progression to end-stage kidney disease and recurrence after transplantation (144, 192, 193).

In a heterogenous group of patients with thrombotic microangiopathy, the localization and intensity of staining of C5b-9 did not correlate with the presence of immunoglobulins or histological signs of active thrombotic microangiopathy (100).

## Vasculitis

ANCA-associated vasculitis manifests as a crescentic and necrotizing glomerulonephritis with scarce deposits of immunoglobulins or complement factors, referred to as pauci-immune. Nonetheless, factors of the alternative pathway, including C3, FB, and properdin, can be found in the glomerulus. Activation of the alternative pathway and the subsequent formation of C5a are essential in its pathogenesis, while their inhibition attenuates the development of kidney injury in both animal models and human patients (14, 22, 23).

Staining of C5b-9 was more frequent and more intense in patients with ANCA-associated vasculitis than in healthy individuals (22, 116, 139). It was found in the glomerulus (53, 116, 139, 143, 194), both in the mesangium and along the capillary wall (22, 116), in a patchy and granular pattern, colocalized with C3d, FB, and properdin (22, 116, 143, 194). Staining was predominant in glomeruli with crescents (116, 194). It was furthermore seen granularly in the vascular wall (22, 116). No glomerular or vascular staining was found in one case with thrombotic microangiopathy (130).

**Figure 9:**
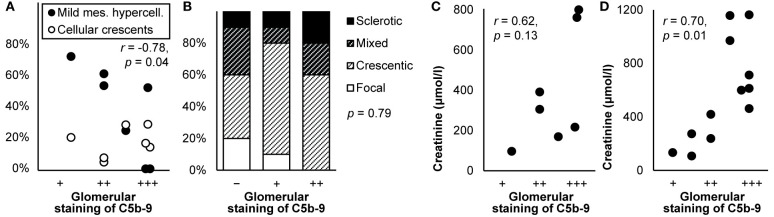
Deposits of C5b-9 in ANCA-associated vasculitis: examples of correlations with histological lesions and clinical characteristics. **(A)** The average staining intensity of C5b-9 in glomeruli is shown in relation with the percentage of glomeruli with mild mesangial hypercellularity and that with cellular crescents in patients with myeloperoxidase antineutrophil cytoplasmic antibody (ANCA)-associated vasculitis (*n* = 7). The antibody used for staining was unspecified. The correlation coefficient for mild mesangial hypercellularity is given; that for cellular crescents was nonsignificant. We plotted previously published individual data (116). **(B)** Staining intensity of C5b-9 in the glomerulus is shown in relation with histological patterns in patients with renal ANCA-associated vasculitis (*n* = 25). Antibody ab55811 was used for staining. Differences between staining intensities were tested with Fisher’s exact test. We plotted previously published individual data (53). **(C)** The average staining intensity of C5b-9 in glomeruli is shown in relation with serum creatinine at the time of biopsy in patients with myeloperoxidase antineutrophil cytoplasmic antibody (ANCA)-associated vasculitis (*n* = 7). The antibody used for staining was unspecified. We plotted previously published individual data (116). **(D)** The average staining intensity of C5b-9 in glomeruli is shown in relation with serum creatinine at the time of biopsy in patients with ANCA-negative pauci-immune crescentic glomerulonephritis (*n* = 12). The antibody used for staining was unspecified. We plotted previously published individual data (117). Relations were tested with Pearson’s correlation (*r*).

Glomerular staining intensity of C5b-9 was lower in glomeruli that were normal, mildly hypercellular (116), or focally affected (53), as illustrated in **Figures 9A, B**. It correlated with proteinuria (*r* = 0.63, *p* < 0.001) in one (22) but not another study (53). The frequency, extent, and intensity of glomerular staining of C5b-9 did not correlate with the type of ANCA, clinical vasculitis activity, eGFR, serum and urine levels of sC5b-9 or C3, the presence of glomerulosclerosis, crescents, thrombotic microangiopathy, interstitial fibrosis, or tubular atrophy, or the occurrence of end-stage renal disease or death (22, 53, 116, 194), except for a trend toward higher creatinine in patients with more intense staining (116), as illustrated in **Figure 9C**.

Similar findings were reported for patients with ANCA-negative pauci-immune crescentic glomerulonephritis. They had granular deposits of C5b-9 in the mesangium, along the capillary wall, and in the vascular wall, more often and more intense than in healthy kidneys. Deposits were predominant in crescents. They colocalized well with C3d and, if present, C4d and FB. Glomerular staining intensity did not correlate with age, sex, hemoglobin, proteinuria, or dependence on dialysis but correlated with creatinine (117), as shown in **Figure 9D**.

Among patients with idiopathic rapidly progressive glomerulonephritis, of whom three-quarters were ANCA-positive, deposits of C5b-9 were present in the glomerulus, the vascular wall, and a third of the tubules and prominent in fibrocellular and fibrous crescents. Staining was independent of presence and type of ANCA. Tubular, but not glomerular, staining of C5b-9 correlated with markers of inflammation and fibrosis, creatinine, and a lack of treatment effect (195, 196).

## General Patterns of Kidney Injury

### Interstitial Nephritis

Formation of C5b-9 participates in the development of interstitial inflammation and fibrosis, but the mechanisms are unclear (197). As one explanation, the alterative pathway may be activated in the tubules and peritubular interstitium due to modification of C3 by ammonia, produced as a result of proteinuria (198). The C5b-9 formed there is partly excreted in the urine, more so in severe forms of acute tubulointerstitial nephritis (31).

In patients with acute tubulointerstitial nephritis, staining of C5b-9 was weak in the glomerulus and vascular wall, similar to healthy kidneys (70, 96, 143, 199) but more intense in the interstitium and along the tubular basement membrane as compared with healthy kidneys or kidneys with acute tubular necrosis (31, 70, 199). It covered 39% of tubules (31). Tubular and vascular staining were most diffuse and intense in areas of interstitial inflammation and fibrosis (70, 96, 139, 143). Across various underlying glomerulopathies, the extent and intensity of tubular staining correlated with the severity of interstitial inflammation (*r* = 0.84, *p* < 0.001) and interstitial volume (*r* = 0.79, *p* < 0.001) (139).

Patients with juvenile nephronophthisis, a congenital ciliopathy with chronic tubulointerstitial nephritis and tubular cysts, also had more frequent and more intense tubular staining than healthy individuals. Staining was associated with signs of apoptosis and striated membranous structures (118).

### Acute Tubular Necrosis

Deposition of C5b-9 in tubules—and elsewhere in the kidney—has been proposed as a physiological mechanism for removal of cell remnants (94), but it is also a pathogenic mechanism by which activation of the alternative pathway causes kidney injury after ischemia and reperfusion, a common cause of acute tubular necrosis (136, 197, 200), or during proteinuria (20, 198, 201). In animal models of ischemia and reperfusion injury and of proteinuria, deficiency of C5 or C6 protects against tubular deposition of C5b-9 and acute tubular necrosis (198, 201, 202).

Patients with acute tubular necrosis had segmental thick linear deposits of C5b-9 along the tubular basement membrane, primarily in proximal tubules and atrophic tubules and similarly to C3 (94, 135, 136, 200). Tubular, but not glomerular or vascular, deposits were more frequent, widespread, and intense than in patients without tubular atrophy and necrosis or without kidney disease (94, 136). Deposits were not seen in or on tubular cells (136). They covered 15% of tubules in acute tubular necrosis due to medication or autoimmune disease (31), but the majority of tubules in most cases of acute tubular necrosis due to medication, sepsis, or ischemia-reperfusion after kidney transplantation (136).

Six autopsy cases of COVID19 with acute loss of eGFR exhibited common acute tubular necrosis, variable interstitial inflammation, and minimal glomerular lesions. All had deposits of C5b-9 on tubular cells, together with viral antigens, while two had sparse deposits in the glomerulus and in the vascular wall (125).

One case of adenovirus-associated hemorrhagic cystitis, characterized by severe tubular degeneration and necrosis, but minimal interstitial inflammation or glomerular lesions, had coarse granular deposits of C5b-9 along the tubular basement membrane and, with less intensity, along Bowman’s capsule. They colocalized with C3 and adenoviral antigens (203).

Across various glomerulopathies, the extent and intensity of tubular staining of C5b-9 correlated with the extents of degenerative lesions of the tubular basement membrane, including thickening (*r* = 0.51, *p* < 0.05), lysis (*r* = 0.77, *p* < 0.05), detachment of tubular cells (*r* = 0.46, *p* < 0.05), and membranous structures to which C5b-9 was bound (*r* = 0.75, *p* < 0.05) (142).

### Reflux Nephropathy

Chronic urolithiasis, chronic vesicoureteral reflux, and chronic pyelonephritis, which characterize reflux nephropathy, expose the kidney to bacterial pathogens that activate the classical and alternative complement pathways. Inhibition of their activation prevents kidney injury in animal models (204–206). In three small studies on reflux nephropathy, deposits of C5b-9 were not or scarcely found in histologically normal glomeruli—similarly to healthy kidneys—but as intense coarse granules in areas of glomerulosclerosis together with C3 and properdin. Podocytes had regressed in these areas. Deposits were furthermore found along the tubular basement membrane without C3 (44, 75, 127) and extensively in the vascular wall (44).

## Kidney Tumors

In clear cell renal cell carcinomas, no deposits of C5b-9, but abundant deposits of C1q and pentraxin-3 were present (207), the latter of which can activate the complement pathways in various ways (208). In various types of renal cell carcinomas, staining of C5b-9 was similarly absent or weakly present in only a sixth to a tenth of tumors, covering not more than half of each tumor (138, 209). Enhanced expression of CD59 and other inhibitory factors might explain the absence of C5b-9 (138, 207, 209). Yet, in another study on various types of renal cell carcinomas, staining of C5b-9 was weak in 55% and moderate in 27% of tumors, despite enhanced expression of inhibitory factors. The tumors could be partitioned into those with deposits of only C3 due to activation of the alternative pathway—with much necrosis as a cause or consequence —, those with deposits of IgG and C1q due to activation of the classical pathway, and those without immune deposits. Although present in all three groups, stainings of C5b-9 and inflammatory markers were most intense in tumors with activation of either pathway (210).

## Kidney Transplantation

During kidney transplantation, the donor’s death and the transplant’s surgical excision, transportation, and reperfusion all contribute to activate the complement pathways. The extent of complement activation influences the function of the kidney transplant. The serum level of sC5b-9 is elevated in deceased donors and predicts the risk of acute rejection and chronic graft failure after transplantation. Deposits of C5b-9 in transplants are not taken into account—contrary to the routine assessment of deposits of C4d, especially in peritubular capillaries—as a diagnostic criterion for antibody-mediated rejection as part of the Banff classification (16, 26, 29). Complicating the interpretation of their relevance, deposits of C5b-9 in kidney transplants may result from physiological deposition in the donor as in healthy kidneys, from kidney disease in the donor, from the transplantation itself, from rejection in the recipient, as well as from *de novo* or recurrent kidney disease in the recipient.

### Ischemia and Reperfusion Injury

Ischemia and reperfusion—inevitable consequences of transplantation—induce acidosis and reactive oxygen species, which both lead to activation of the lectin and alternative pathways and subsequent inflammation, especially in the tubulointerstitium. Inhibition of C5b-9 formation ameliorates the inflammation (13, 16, 26). Nonetheless, in human kidney transplants, deposits of C5b-9 were absent from the tubules and vascular wall both before and shortly after reperfusion, despite a transient elevation of sC5b-9 in arteriovenous samples in between (211). This may explain why eculizumab does not prevent delayed transplant function (13, 16, 26). On the other hand, once kidney transplants suffered from delayed function, C5b-9 appeared in the glomerulus and tubules (54).

### Kidney Transplant Rejection

Antibodies against donor antigens on the transplant’s endothelium activate the classical pathway (13, 16, 26). As a result, in acutely rejected transplants, deposits of C5b-9 were present in the glomerulus and vascular wall with higher staining intensities than in healthy kidneys and with variable staining intensity along the tubular basement membrane (54, 67, 70, 83, 100, 104, 130, 143, 211, 212). The proportion of glomeruli that contained deposits varied widely between 8 and 77% (54). In the glomerulus, deposition was restricted to the mesangium (67, 96, 212), extended along the capillary wall (70, 100), or was restricted to the capillary wall (143). Tubular and vascular deposits were concentrated in areas of sclerosis (67, 70, 143). C5b-9 was absent from peritubular capillaries, despite the presence of C4d, which was explained by concurrent presence of CD59 (83, 130, 212). In one group of patients biopsied a week after transplantation according to protocol, of whom the majority experienced acute rejection, no deposits were found other than those found at the time of transplantation (129). Glomerular and tubular depositions did not correlate with each other or with age, sex, creatinine, proteinuria, HLA mismatch, or the severity of rejection (54, 83). Depositions throughout the kidney diminished strikingly in three days after acute antibody-mediated rejection was successfully treated with eculizumab in one (104) but not another case (100). The efficacy of eculizumab to prevent or treat rejection remains uncertain (13, 16).

Chronically rejected transplants had similar deposition of C5b-9 as acutely rejected transplants (54). In a group of patients with acute or chronic antibody-mediated rejection together, weak, granular, and subendothelial staining along the capillary wall was found in 24% and staining in the peritubular capillaries in 2%, whereas staining of C4d was present in both localizations in almost all patients. Those with global and diffuse glomerular staining of C5b-9 had a lower eGFR (26 vs. 34 ml/min/1.73 m^2^, *p* = 0.04), more often double contours (100 vs. 40%, *p* = 0.01), and a higher Banff score (1.7 vs. 0.8, *p* = 0.01). They also had a shorter transplant survival (median 6 vs. 41–44 months, *p* = 0.02), though not after adjustment for other risk factors (134).

One study compared deposition of C5b-9 in biopsies conducted because of a clinical suspicion of rejection and biopsies conducted according to protocol in patients with ABO-incompatible transplants. Almost all rejections were acute T-cell mediated; the numbers of confirmed rejections were not reported. Deposition of C5b-9 was more common in the glomerulus, tubules, and peritubular capillaries in the clinically indicated biopsies, whereas depositions of C1q, C3c, and C4d were similar. Peritubular C5b-9 in these biopsies correlated with titers of anti-ABO antibodies before transplantation (*r* = 0.72, *p* = 0.002) and with the occurrence of rejection (*r* = 0.52, *p* = 0.02) (213).

### *De Novo* Kidney Disease After Transplantation

Deposition of C5b-9 in kidney diseases arising after kidney transplantation was similar as in native kidneys. Among patients who developed *de novo* membranous nephropathy, deposits were restricted to the mesangium as fine granules in those with stage I and were localized along the capillary wall together with immune deposits in stage II (214). Cases who developed thrombotic microangiopathy without rejection—a common phenomenon, often without a clear cause (26)—had few deposits in the mesangium, but many deposits in the vascular wall, similar to cases without thrombotic microangiopathy (100).

## Discussion

This review is the first to provide an overview of studies on deposition of C5b-9 in healthy and diseased human kidneys. Other reviews have summarized the various mechanisms through which C5b-9 exerts its lytic and sublytic effects on kidney cells (43, 64, 88, 101, 187, 197, 215–217).

In healthy kidneys, staining of C5b-9 was absent, weak in the mesangium, or more prominent in the glomerular vascular pole and the extraglomerular vascular wall, for which we discuss possible explanations in the section on healthy kidneys. Across a wide spectrum of kidney diseases—excluding minimal change nephropathy and glomerular basement membrane diseases—staining of C5b-9 was more frequent, extensive, and intense, as outlined in **Figure 1** and detailed in **Supplementary Table 2**.

In kidney diseases due to deposition of immune complexes and kidney diseases due to activation of the alternative pathway, glomerular deposits of C5b-9 colocalized with immune deposits containing immunoglobulins or other complement factors (44, 57, 67, 73, 75, 81, 96, 126, 143, 218). Correspondingly, glomerular staining of C5b-9 was more frequent, diffuse, and intense than in healthy kidneys and kidney diseases without immune deposits (44, 67, 75, 96, 126, 143), was found along the capillary wall in membranous nephropathy and lupus nephritis class V, in the mesangium in IgA nephropathy and lupus nephritis classes III and IV, and throughout the glomerulus in C3 glomerulopathy, thrombotic microangiopathies, and vasculitis. Studies generally regarded these deposits of C5b-9 as most likely locally formed along with the immune deposits as part of the cause of disease.

In all kidney diseases, deposits of C5b-9 were prominent in areas of glomerulosclerosis, tubulointerstitial injury, and vascular hyalinosis and sclerosis. This finding was clearest in hypertensive and diabetic nephropathy, interstitial nephritis, and acute tubular necrosis. These deposits did not consistently colocalize with immunoglobulins or other complement factors (19, 27, 44, 54, 67, 70, 72, 73, 75, 94–97, 103, 118, 127, 134, 139, 141, 175, 200), although C5b-9 and C3 colocalized more often in areas of glomerulosclerosis when immune deposits were present in other areas of the glomerulus (44, 67, 143) and both C5b-9 and C3 were more prominent in tubules and arteries in areas of tubulointerstitial injury (44, 57, 67, 75, 94, 96, 135, 139, 143, 200). These deposits may either be formed locally when complement pathways are activated by cellular injury or originate in urine or blood when sC5b-9 passes the tubular or vascular wall. sC5b-9 can be formed in or excreted into the tubular lumen, particularly in presence of proteinuria (20, 33, 198, 201). The observation that C5b-9 resided on both sides of the tubular basement membrane, but C3 only on the interstitial side (44), fits with an origin in the tubular lumen. Studies generally regarded these deposits of C5b-9 as a nonspecific consequence of kidney injury rather than a cause of kidney disease.

Across kidney diseases, deposits of C5b-9 seemed associated with cell membrane fragments rather than bound to cells themselves, as revealed by immunoelectron microscopy. Cells may have shed these fragments after C5b-9 has bound the cells or C5b-9 may have bound these fragments after having been shed by cells, as discussed in the section on staining techniques. Both processes, though, contribute to cellular activation, proliferation, inflammation, sclerosis, and fibrosis.

Studies using immunohistochemical staining cannot unravel whether deposits of C5b-9 are a cause of kidney disease or a consequence of kidney injury and cannot distinguish between C5b-9 that has bound cells, has been shed by cells, has bound extracellular vesicles, or has remained soluble. **Table 3** summarizes these and other inherent limitations.

**Table 3:** Summary of the limitations and remaining questions of immunohistochemical studies on deposition of C5b-9 in human kidneys.

**Inherent limitations of current studies in general**
They cannot unravel whether deposits of C5b-9 are a cause of kidney disease or a consequence of kidney injury. They cannot distinguish between locally formed C5b-9 bound to cells, C5b-9 bound to or shedded as extracellular vesicles, and sC5b-9 originating in urine or blood. They cannot assess when deposits have arisen, so that, given their slow clearance, deposits may have chronically accumulated. They evaluate staining subjectively and semiquantitatively.
**Specific limitations of current included studies**
Included patients were generally ill-characterized. Staining techniques were often described very concisely. Different staining techniques and antibodies were seldomly compared. The method of evaluating staining was mostly undefined. The method of evaluating staining was variable. As examples, traces of staining were usually considered negligible but sometimes counted as positive (18) and scoring systems were used incidentally and incomparably (19, 31, 52, 83, 84, 87, 94, 116, 117, 135, 136, 139, 142, 209). Variability of staining among individual patients with the same kidney disease was rarely documented, while it might be large (57). Staining across different kidney diseases was directly compared in only few studies (44, 57, 67, 70, 72, 75, 79, 84, 96, 98, 126, 141–143). Colocalization with immunoglobulins and other complement factors, especially in tubules and vessels, was reported only briefly. Correlations between deposits and histological lesions or clinical characteristics were not studied systematically. Changes in staining were uncommonly tracked through time or treatment.
**Remaining questions for future studies**
Does staining of C5b-9 differ when directly comparing staining techniques and antibodies? Is staining more common in tissue obtained with autopsy than biopsy or nephrectomy? And can this be explained by a different selection of patients? How do deposits of C5b-9 differ between kidney diseases due to deposition of immune complexes, due to activation of the alternative pathway, and due to other mechanisms? How do deposits vary among patients with the same kidney disease? Are deposits dependent on the age and sex of patients? With which immunoglobulins and other complement factors do deposits colocalize in various localizations and in various kidney diseases? What structures are associated with deposits on immunoelectron microscopy? How fast are deposits cleared in various kidney diseases and across individual patients with the same kidney disease? How do deposits change through time and treatment? And how does the change relate to variable activation of complement pathways, for example in membranous nephropathy? Do deposits consistently predict prognosis and treatment effect? And how does this depend on their localizations and on the underlying kidney disease?

Whether C5b-9 is a cause of kidney disease or a consequence of kidney injury does not affect its potential as a prognostic marker. Deposition of C5b-9 indicates that complement activity has resulted in formation of both C5a and C5b-9, both of which may participate in the causation of disease and the response to tissue injury. Indeed, the presence and intensity of staining of C5b-9 correlated with histological lesions, clinical characteristics, prognosis, and treatment effects in various kidney diseases, as summarized in **Table 1**. Illustrations of such correlations are given in the figures, while a complete discussion of possible correlations is given in the text.

Further analytical comparisons and firm conclusions were hampered by a lack of detailed data and descriptions of methods and results in the included studies, as summarized in **Table 3**. As a consequence, we could not precisely specify differences in deposition of C5b-9 as dependent on staining techniques and between kidney diseases due to deposition of immune complexes, kidney diseases due to activation of the alternative pathway, and kidney diseases due to other mechanisms.

Future studies are necessary to overcome the limitations of current studies, to confirm our findings, and to answer remaining questions as proposed in **Table 3**. To facilitate analytical comparisons, future studies should systematically study deposition of C5b-9 in well-described populations and tissues with detailed data and descriptions of their methods and results. Immunohistochemical studies may be strengthened by a combination with other techniques, such as immunoelectron microscopy or mass spectrometry of microdissected glomeruli, which are more objective, sensitive, and quantitative (90–92, 124, 180).

In this review, we aim to motivate and guide future studies on deposition of C5b-9 in human kidneys by summarizing the available data and by identifying the data that still lack. We describe when deposition of C5b-9 in kidneys may be regarded a cause of kidney disease and when a consequence of kidney injury. We substantiate that staining of C5b-9 in kidneys, although not yet routinely conducted, promises to be valuable for evaluating activation of complement pathways, estimating prognosis, and identifying possible treatment targets.

## Author Contributions

JK and HR conceived and designed this review. JK and ME collected literature. JK drafted the manuscript, designed the tables, and drew the figures. ME contributed to the drafting of the manuscript and to the design of tables. All authors contributed to the article and approved the submitted version.

## Funding

JK was supported with a Niels Stensen Fellowship and with a travel grant of the NVLE Fund. ME was supported with a grant from the Dutch Kidney Foundation (COMBAT grant 130CA27).

## Conflict of Interest

The authors declare that the research was conducted in the absence of any commercial or financial relationships that could be construed as a potential conflict of interest.

## Abbreviations

aHUS, atypical hemolytic uremic syndrome; ANCA, antineutrophil cytoplasmic antibody; COVID19, coronavirus disease 2019; CR1, complement receptor 1; DAF, decay-accelerating factor; eGFR, estimated glomerular filtration rate; FB, complement factor B; FD, complement factor D; FHR, complement factor H related protein; FH, complement factor H; GFR, glomerular filtration rate; HBe, hepatitis B e antigen; HBs, hepatitis B surface antigen; HLA, human leukocyte antigens; MASP, mannose-associated serine protease; MBL, mannose-binding lectin; PLA2R, phospholipase A2 receptor; *r*, Pearson’s correlation coefficient; *ρ*, Spearman’s correlation coefficient; SLE, systemic lupus erythematosus; STEC-HUS, hemolytic uremic syndrome elicited by infection with Shiga toxin-producing enterohemorrhagic *Escherichia coli*; TTP, thrombotic thrombocytopenic purpura.
