# Supplementary material for: Deposition of the Membrane Attack Complex in Healthy and Diseased Human Kidneys
Source: Front Immunol. 2021 Feb 11;11:599974. doi: 10.3389/fimmu.2020.599974 (PMC7906018; doi:10.3389/fimmu.2020.599974)
Supplement: Supplementary file 1 [file DataSheet_1.pdf]

SUPPLEMENTARY MATERIAL

- **Methods**.
- **Supplementary Table 1**:
  Methodological characteristics of studies on deposition of C5b-9 in healthy and diseased human kidneys.
- **Supplementary Table 2**:
  Deposits of C5b-9 in healthy and diseased human kidneys.
- **Supplementary Table 3**:
  Deposits of C5b-9 in healthy and diseased human kidneys as detected with different antibodies.
- **Supplementary Figure 1**:
  Deposits of C5b-9 in healthy human kidneys as detected with different antibodies.
- **Supplementary Figure 2**:
  Deposits of C5b-9 in diseased human kidneys as detected with different antibodies.
- **Supplementary Figure 3**:
  Deposits of C5b-9 in healthy human kidneys as detected with different staining techniques.
- **Supplementary Figure 4**:
  Deposits of C5b-9 in diseased human kidneys as detected with different staining techniques.
- **Supplementary Figure 5**:
  Deposits of C5b-9 in healthy human kidneys as detected in different tissue sources.
- **Supplementary Figure 6**:
  Deposits of C5b-9 in diseased human kidneys as detected in different tissue sources.

METHODS

We searched for relevant literature on Medline, PubMed, Web of Science, and Google Scholar, using the search terms “C5b-9”, “C5b9”, “C5b-C9”, “membrane attack complex”, or “terminal complement complex” in combination with “kidney” or “renal”. We screened reference lists of included studies for additional relevant studies. We included 141 studies on deposition of C5b-9 in *in vivo* human kidney tissue. We excluded studies on *in vitro* kidney tissue, studies on animal kidney tissue, and studies that lacked an English abstract. The methodological characteristics of the included studies are summarized in **Supplementary Table 1** below. The data reported by the included studies are listed in **Supplementary Table 2** below.

We excluded small numbers of cases from our discussion that had unclear or unclassifiable kidney diseases without a definition, such as “angiitis” or “focal hyalinosis and sclerosis”, which were predominantly found in older studies. Kidney diseases that were studied only incidentally are listed at the end of **Supplementary Table 1** without a discussion in the text.

We followed the classifications as used in the original studies to regard kidney tissue as healthy and to group kidney diseases. We followed the terminologies as used in the original studies to describe the localization, pattern, and intensity of staining. Glomerular staining includes staining in the mesangium and/or along the glomerular capillary wall without further specification. We followed the classification of staining as absent or present as described in the original studies. We identified the antibodies used for staining according to their clone names given in the original studies and regarded them as unknown if a clone name was not given.

We cautiously interpreted studies that used a combination of antibodies against individual components of C5b-9, such as C6 and C9, instead of a selective antibody to stain deposits of C5b-9, because of the limitations discussed in the text.

Our findings derived from the included studies are summarized in **Figure 1** and **Table 1**. For **Figure 1**, we calculated the proportions of studies that reported staining of deposits of C5b-9 to be either absent or present relative to the total number of these studies, we calculated the medians of the proportions of patients reported in the studies to exhibit staining, and we calculated the medians of the staining intensities reported in the studies, all separately for different localizations of the staining and for different kidney diseases. We omitted studies that did not report the respective data from each calculation. We omitted studies that included only one patient from the calculation of the medians of the proportions of patients reported to exhibit staining. We omitted patients exhibiting no staining from the calculation of the medians of the staining intensities. For comparability, we rescaled staining intensities that were reported on a scale from − to ++++ to the most commonly used scale from − to +++. In **Table 1**, we listed the histological lesions and clinical characteristics that were reported to correlate with staining of deposits of C5b-9 and are discussed in detail in the text, all separately for different localizations of the staining and for different kidney diseases.

In addition, we drew figures to illustrate how deposition of C5b-9 was related with histological lesions or clinical characteristics in various kidney diseases. We reproduced figures from their original publications or newly drew figures based on individual data reported in the original publications, as indicated in the captions of the figures. For this, we selected all available published figures and data that revealed a possible correlation between staining of C5b-9 and histological lesions or clinical characteristics.

In the text, we describe relevant correlation coefficients and other statistical measures as reported in the original studies. As two exceptions in the section on lupus nephritis, we present an odds ratio of 0.60 that we calculated with logistic regression from individual data reported in the original study and an odds ratio of 0.22 that we inverted for clarity from the ratio reported in the original study. Throughout, we indicate Pearson’s correlation coefficient as r and Spearman’s correlation coefficient as ρ. We considered $p<0.05$ as statistically significant, but took into account that p values might be overestimated in studies that included only small numbers of patients.

SUPPLEMENTARY TABLE 1 | Methodological characteristics of studies on deposition of C5b-9 in healthy and diseased human kidneys

Ref.	Year	Case description	*N*	Ages[a]	Males	Tissue	Method of staining of C5b-9[b]: Antibody	Method of staining of C5b-9[b]: Technique	Additional staining	Correlates
**Healthy kidneys**										
95	1981	Unkn.	3	Unkn.	Unkn.	Unkn.	Kolb 1975	Dir.fr.fluor.	Igs, C1q, C3, C9	None
44	1983	Unkn.	Unkn.	Fetus	Unkn.	Unkn.	PolyC9-MA	Indir.fr.fix.fluor.	Igs, C1q, C3, C4, C5, C6, C7, C8, C9	None
		Unkn.	Unkn.	Adults	Unkn.	Unkn.	Idem	Idem	Idem	None
46	1985	Unkn.	Unkn.	Unkn.	Unkn.	Autopsy	aE11	Indir.fluor.	Igs, C3	None
96	1986	Isolated microscopic hematuria	6	Adults	Unkn.	Biopsy	Anti-C5b-9(m)	Indir.fr.fluor., imm.electr.	Igs, C1q, C3b/C3c, C3d, C3g, C5, C8, C9, FH	None
		Kidney carcinoma	2	Adults	Unkn.	Nephrect.	Idem	Idem	Idem	None
67	1987	No histological lesions on light, immunofluor., or electron microscopy	6	Unkn.	Unkn.	Biopsy	PolyC9-MA	Indir.fr.fluor.	Igs, C3, C5, Fn, Vn	None
126	1987	Traumatic death	3	0-65	Unkn.	Autopsy or unkn.	PolyC9-MA	Indir.fr.fix.fluor., imm.electr.	Igs, C3, C5	Age
127	1987	Kidney calculus, kidney tumor, or kidney trauma without histological lesions	4	Unkn.	Unkn.	Unkn.	PolyC9-MA	Indir.fr.fluor.	Igs, C1q, C3, C4, C5, C9, P, Fn, vimentin, collagen, laminin, cytokeratin, uromodulin	None
76	1987	Unkn.	3	Unkn.	Unkn.	Autopsy	C5 and C9	Dir.fr.fluor.	Igs, C1q, C3, C4, Vn	None
137	1987	Unkn.	2	Unkn.	Unkn.	Nephrect.	PolyC9-MA	Indir.fr.fix.fluor.	IgA, C3	Age
52	1987	Clinical signs of glomerulopathy without lesions on immunofl. microscopy	4	5-37	75%	Biopsy	Anti-MAC-neo	Indir.fr.fix.fluor.	Igs, C1q, C3b/C3c, C3d, C4, C5, C6, C9, P, FB, FH, Fn, Vn	Age, sex, blood pressure, creat., proteinuria, hematuria, histological lesions
97	1988	Kidney calculus, kidney tumor, or kidney trauma without histological lesions	4	43-66	Unkn.	Unkn.	PolyC9-MA	Indir.fluor., imm.electr.	Igs, C3, C4, C5, C9	None

70	1989	Isolated microscopic hematuria	19	Unkn.	Unkn.	Biopsy	C6 and C9	Indir.fr.fluor.	Igs, C1q, C3, Cn, Vn	None
		Kidney transplants without rejection	9	Unkn.	Unkn.	Idem	Idem	Idem	Idem	None
72	1989	Isolated microscopic hematuria	3	Adults	Unkn.	Biopsy	Anti-C5b-9(m)	Indir.fr.fluor., imm.electr.	Igs, C3, Vn	None
77	1989	No histological lesions	8	Unkn.	Unkn.	Autopsy	Unkn.	Indir.fr.fluor.	Igs, C1q, C3, C4, Vn	None
75	1993	Kidney tumor or kidney trauma	Unkn.	Unkn.	Unkn.	Nephrect.	PolyC9-MA	Indir.fr.fix.fluor.	Igs, C3c, C3d, C5, C9, Vn, Vn receptor	None
135	1993	Kidney transplants or traffic accidents with-out lesions on light or immunofl. microscopy	8	Unkn.	Unkn.	Unkn.	Unkn.	Indir.fr.fix.fluor.	Lysozyme, α1-antitrypsin, α1-antichymotrypsin	None
110	1995	Kidney donors before transplantation	4	Unkn.	Unkn.	Nephrect.	aE11	Indir.fr.fix.perox.	Igs, C3, CD3, CD68, HLA-DR	None
94	1995	Unkn.	8	Unkn.	Unkn.	Unkn.	Unkn.	Indir.fr.fix.fluor.	Igs, C1q, C3c, C3d, C4, C5, C6, C7, C8, C9	None
111	1996	Kidney donors before transplantation or Wilms' tumor	7	Unkn.	Unkn.	Nephrect.	WU-7,2	Indir.fr.fix.perox.	CR1	Histological lesions, histological activity index
138	1996	Renal cell carcinoma	10	Unkn.	Unkn.	Unkn.	PolyC9-MA	Indir.fr.fix.perox.	C3, CD59, DAF, MCP	None
139	1997	Early-stage renal cell carcinoma without infiltrating lymphocytes, pyelonephritis, or lesions on immunofl. microscopy	6	47-75[c]	Unkn.	Nephrect.	Unkn.	Indir.fr.fix.perox.	Igs, C3, MCP	Interstitial mononuclear infiltration, relative interstitial volume
128	2001	Kidney malignancy	5	Unkn.	Unkn.	Nephrect.	aE11	Indir.fix.fr.perox.	IgA, C3, C3d	None
112	2002	Kidney donors before transplantation	5	Unkn.	Unkn.	Biopsy	aE11	Indir.fix.fr.perox.	CD3, CD68, HLA-DR, α3β1-integrin, LFA1, ICAM1	None
129	2003	Kidney donors shortly after transplantation	15	21-72[d]	Unkn.	Biopsy	aE11	Indir.fr.fix.fluor.	C1q, C3c, C4d, C6, MBL, MASP1, FB	None

84	2004	Normal kidney function without hypertension, diabetes, urinary abnormalities, or lesions on light or immunofl. microscopy	12	50-88	50%	Autopsy	Unkn.	Indir.fix.fr.perox.	Igs, C1q, C3, C4d, Vn, CD59, CML, αSMA, ssDNA	None
83	2004	Kidney transplants before reperfusion	10	Unkn.	Unkn.	Biopsy	Unkn.	Indir.fr.fluor.	Igs, C1q, C3, C4, C4d, CD59	None
113	2004	Unkn.	Unkn.	Unkn.	Unkn.	Biopsy	Unkn.	Indir.fix.perox.	Glycated CD59	None
114	2006	Asymptomatic normocomplementemic hematuria	7	Children	Unkn.	Unkn.	1B4	Dir.fr.fix.perox.	None	None
115	2008	Kidney tumor	Unkn.	Unkn.	Unkn.	Nephrect.	aE11	Indir.fix.perox.	Igs, C1q, C3, C4, CD68, TGFβ1, α3β1-integrin, αSMA	None
90	2009	Kidney transplants without histological lesions	5	Unkn.	Unkn.	Biopsy	Not applicable	Liquid chromato-graphy and mass spectrometry	Not applicable	None
116, 117	2009, 2010	Macroscopic hematuria or renal carcinoma without histological lesions on light, immunofl., or electron microscopy	2	Unkn.	Unkn.	Nephrect. or unkn.	Unkn.	Indir.fr.fix.fluor.	Igs, C1q, C3c, C3d, C4d, MBL, FB	None
118	2011	Unkn.	1	Unkn.	Unkn.	Unkn.	Unkn.	Unkn.	Igs, C3, TINag, Fas antigen, collagen, nephrocystin 1	Not applicable
103	2012	Unkn.	6	Unkn.	Unkn.	Biopsy	aE11	Indir.fluor.	Igs, C1q, C3	None
119	2013	No kidney disease	5	Unkn.	Unkn.	Autopsy	WU-13,15	Indir.fr.fluor.	Igs, C1q, C3, MBL, ficolin-2	None
120	2013	Kidney cancer	1	Adult	100%	Nephrect.	Unkn.	Indir.fix.perox.	C3	Not applicable
121	2014	Kidney carcinoma	1	Unkn.	Unkn.	Nephrect.	Unkn.	Indir.fix.perox.	Igs, C1q, C3c, C3d, C4d, P, FB, fibr.	None
131	2015	Pregnant women without hypertension	25	Unkn.	0%	Autopsy	Unkn.	Indir.fix.perox.	Igs, C1q, C3d, C4d, MBL, P, caspase 3	None
130	2015	Kidney transplants unsuitable for transplantation	9	Unkn.	Unkn.	Unkn.	A239	Indir.fix.perox.	C1q, C4d, MBL	None

122	2016	Renal cancer	1	Adult	100%	Nephrect.	Unkn.	Fluor.	None	None
123	2017	Slight proteinuria or hematuria without histological lesions	14	7-62[c]	57%	Biopsy	Unkn.	Indir.fix.fluor.	MBL, LC3, p62, synaptopodin	None
124	2017	Suspected glomerulo-pathy with normal kidney function, no proteinuria, no or minimal histological lesions, and no kidney failure during ten years of follow-up	15	8-56[c]	47%	Biopsy	aE11	Indir.fix.perox., liquid chromato-graphy and mass spectrometry	C1q, C3c	None
92	2017	Unkn.	5	Unkn.	Unkn.	Protocol biopsy	Not applicable	Liquid chromato-graphy and mass spectrometry	Not applicable	None
133	2018	Incidental trauma	3	Unkn.	Unkn.	Unkn.	Unkn.	Fluor.	None	None
136	2018	Normal kidney function despite sepsis or septic shock, administration of colistin or contrast, kidney transplantation, or other risk factor of acute kidney injury	74	29-95[c]	69%	Autopsy	Unkn.	Indir.fix.perox.	FH	None
27	2018	No diabetes and no histological lesions	41	30-97[c]	61%	Autopsy	Unkn.	Indir.fix.perox.	C1q, C4d, MBL	None
		Kidney donors	10	Unkn.	Unkn.	Biopsy	Idem	Idem	Idem	None
132	2018	Mild hematuria, minimal change disease, focal segmental sclerosis, autopsy without kidney disease	12	23-83	Unkn.	Autopsy or biopsy	aE11	Indir.fix.perox.	Igs, C3, C4d, ADAMTS13	None
28	2018	Renal carcinoma	11	37-73[c]	45%	Nephrect.	aE11	Indir.fr.perox.	C1q, FB, MBL, MASPs	None
31	2019	Urinary tract malignancy	Unkn.	Unkn.	Unkn.	Nephrect.	aE11	Indir.fluor.	KIM1	None
18	2019	No histological lesions	6	Unkn.	Unkn.	Unkn.	aE11	Indir.fix.perox.	Igs, C3b/C3c, C3d, C9, CD68	None
134	2019	Unkn.	1	Unkn.	Unkn.	Biopsy	B7	Indir.fix.perox.	Igs, C1q, C3, C4d	Not applicable

102	2019	Potential kidney donor with normal kidney function	1	Unkn.	Unkn.	Biopsy	aE11	Indir.fix.perox.	Igs C1q, C3b/ C3c, C3d, C4d, P, FH, FHR1, FHR5, CD68	Not applicable
		Protocol biopsy 6 months after transplantation without lesions on light or immunofl. microscopy	1	Unkn.	Unkn.	Biopsy	Idem	Idem	Idem	Not applicable
125	2020	Unkn.	Unkn.	Unkn.	Unkn.	Unkn.	aE11	Indir.fix.perox.	Unkn.	None
**Minimal change nephropathy**										
96	1986	Minimal change nephrotic syndrome	5	Adults	Unkn.	Unkn.	Anti-C5b-9(m)	Indir.fr.fluor.	Igs, C1q, C3b/C3c, C3d, C3g, C5, C8, C9, FH	IFTA
141	1986	Minimal change nephropathy	3	2-14[d]	Unkn.	Biopsy	Anti-C5b-9(m)	Indir.fr.fluor., indir.fix. fr.perox.	Igs, C1q, C3c, C4, fibr.	None
67	1987	Minimal change disease	7	Unkn.	Unkn.	Biopsy	PolyC9-MA	Indir.fr.fluor.	Igs, C3, C5, Fn, Vn	None
98	1987	Minimal change nephropathy	3	2-14[d]	Unkn.	Biopsy	Anti-C5b-9(m)	Indir.fr.fluor., indir.fix. fr.perox.	Igs, C1q, C3c, C4, fibr.	None
72	1989	Minimal change nephrotic syndrome	3	Adults	Unkn.	Unkn.	Anti-C5b-9(m)	Indir.fr.fluor., imm.electr.	Igs, C3, Vn	None
70	1989	Minimal change nephropathy	3	Unkn.	Unkn.	Biopsy	C6 and C9	Indir.fr.fluor.	Igs, C1q, C3, Cn, Vn	None
57	1989	Minimal change nephropathy	6	Unkn.	Unkn.	Biopsy	Anti-MAC	Indir.fix.perox., dir.fr.fluor.	Igs, C1q, C3, C5, C9, fibr.	None
61	1990	Lipoid nephrosis	3	Unkn.	Unkn.	Biopsy	Xia 1988	Indir.fr.fluor.	Igs, C3c, C5, C6, C7, C8, C9, Vn	None
81	1991	Minimal change nephrotic syndrome	9	Unkn.	Unkn.	Biopsy	PolyC9-MA	Indir.fr.fix.fluor.	Igs, C1q, C3, C4, CD59, CD45, fibr.	None
75	1993	Minimal change nephrotic syndrome	10	Unkn.	Unkn.	Biopsy	PolyC9-MA	Indir.fr.fix.fluor.	Igs, C3c, C3d, C5, C9, Vn, Vn receptor	None
79	1994	Minimal change nephrotic syndrome	2	18-23	50%	Biopsy	A239	Indir.fr.fix.perox. imm.electr.	Vn, Vn receptor	None

139	1997	Minimal change nephrotic syndrome	1	15	100%	Biopsy	Unkn.	Indir.fr.fix.perox.	Igs, C3, MCP	Not applicable
142	1999	Minimal change nephropathy	5	Unkn.	Unkn.	Unkn.	Unkn.	Indir.fix.fluor.	Igs, C1q, C3	Tubular basement membrane morphometry
128	2001	Minimal change nephrotic syndrome	5	15-34	60%	Open biopsy	aE11	Indir.fix.fr.perox.	IgA, C3, C3d	Age, sex, creat., proteinuria, hematuria, serum IgA, C3 mRNA expression
143	2002	Minimal change disease	4	Unkn.	Unkn.	Biopsy	Unkn.	Indir.fr.fix.fluor.	Igs, C1q, C3, FHR5	None
113	2004	Minimal change nephropathy	1	Unkn.	Unkn.	Biopsy	Unkn.	Indir.fix.perox.	Glycated CD59	Not applicable
85	2005	Minimal change nephrotic syndrome	10	Unkn.	Unkn.	Unkn.	Unkn.	Indir.fr.perox.	Igs, C1q, C3c, C4, C4bp, MBL, MASP1, FB, CD59, fibr.	None
86	2007	Minimal change nephritic syndrome	10	Unkn.	Unkn.	Unkn.	Unkn.	Indir.fr.fluor.	Igs, C1q, C3c, C4, MBL, MASP1, FB, CD59, C4bp, fibr.	None
87	2010	Minimal change nephrotic syndrome	10	Unkn.	Unkn.	Unkn.	Unkn.	Indir.fr.fluor.	Igs, C1q, C3c, C4d, MBL, FB, CD59, C4bp	None
116, 117	2009, 2010	Minimal change nephropathy	8	Unkn.	Unkn.	Biopsy	Unkn.	Indir.fr.fix.fluor.	Igs, C1q, C3c, C3d, C4d, MBL, FB	None
121	2014	Minimal change disease	5	Unkn.	Unkn.	Biopsy	Unkn.	Indir.fix.perox.	Igs, C1q, C3c, C3d, C4d, FB, P, fibr.	None
18	2019	Minimal change nephropathy	4	Unkn.	Unkn.	Unkn.	aE11	Indir.fix.perox.	Igs, C3b/C3c, C3d, C9, CD68	None

**Glomerular basement membrane diseases**										
96	1986	Alport's syndrome	1	Adult	Unkn.	Unkn.	Anti-C5b-9(m)	Indir.fr.fluor.	Igs, C1q, C3b/C3c, C3d, C3g, C5, C8, C9, FH	Not applicable
139	1997	Thin basement membrane disease	1	42	0%	Biopsy	Unkn.	Indir.fr.fix.perox.	Igs, C3, MCP	Not applicable
143	2002	Thin basement mem-brane nephropathy	12	Unkn.	Unkn.	Biopsy	Unkn.	Indir.fr.fix.fluor.	Igs, C1q, C3, FHR5	None
85	2005	Thin basement membrane disease	10	Unkn.	Unkn.	Biopsy	Unkn.	Indir.fr.perox.	Igs, C1q, C3c, C4, MBL, MASP1, FB, CD59, C4bp, fibr.	None
86	2007	Thin basement membrane disease	10	Unkn.	Unkn.	Unkn.	Unkn.	Indir.fr.fluor.	Igs, C1q, C3c, C4, MBL, MASP1, FB, CD59, C4bp, fibr.	None
87	2010	Thin basement membrane disease	10	Unkn.	Unkn.	Biopsy	Unkn.	Indir.fr.fluor.	Igs, C1q, C3c, C4d, MBL, FB, CD59, C4bp	None
130	2015	Alport's syndrome	5	Unkn.	Unkn.	Biopsy	A239	Indir.fix.perox.	C1q, C4d, MBL	None
18	2019	Thin basement disease	4	Unkn.	Unkn.	Unkn.	aE11	Indir.fix.perox.	Igs, C3b/C3c, C3d, C9, CD68	None
**Hypertensive nephropathy**										
95	1981	Benign nephrosclerosis	2	Unkn.	Unkn.	Unkn.	Kolb 1975	Dir.fr.fluor.	Igs, C1q, C3, C9	None
44	1983	Hypertensive nephrosclerosis	3	Unkn.	Unkn.	Biopsy or nephrect.	PolyC9-MA	Indir.fr.fix.fluor.	Igs, C1q, C3, C4, C5, C6, C7, C8, C9	None
67	1987	Arterionephrosclerosis	6	Unkn.	Unkn.	Biopsy	PolyC9-MA	Indir.fr.fluor.	Igs, C3, C5, Fn, Vn	None
143	2002	Hypertensive nephrosclerosis	2	Unkn.	Unkn.	Biopsy	Unkn.	Indir.fr.fix.fluor.	Igs, C1q, C3, FHR5	Glomerulosclerosis
84	2004	Benign nephrosclerosis	7	54-84	71%	Autopsy	Unkn.	Indir.fix.fr.perox.	Igs, C1q, C3, C4d, CD59, Vn, CML, αSMA, ssDNA	Loss of vascular smooth muscle cells
		Hypertension without kidney involvement	9	65-88	33%	Idem	Idem	Idem	Idem	Idem
131	2015	Pregnant women with preeclampsia	11	26-40[c]	0%	Autopsy	Unkn.	Indir.fix.perox.	Igs, C1q, C3d, C4d, MBL, P, caspase 3	None
		Young non-pregnant women with chronic hypertension	14	Unkn.	0%	Idem	Idem	Idem	Idem	Idem

**Diabetic nephropathy**									
44	1983	Diabetes mellitus	7	Unkn.	Unkn.	Biopsy or nephrect.	PolyC9-MA	Indir.fr.fix.fluor.	Igs, C1q, C3, C4, C5, C6, C7, C8, C9	None
96	1986	Diabetic glomerulosclerosis	2	Adults	Unkn.	Unkn.	Anti-C5b-9(m)	Indir.fr.fluor., imm.electr.	Igs, C1q, C3b/C3c, C3d, C3g, C5, C8, C9, FH	IFTA
67	1987	Diabetic nephropathy	9	Unkn.	Unkn.	Biopsy	PolyC9-MA	Indir.fr.fluor.	Igs, C3, C5, Fn, Vn	None
126	1987	Insulin-dependent diabetes mellitus with diabetic nephropathy	12	Unkn.	Unkn.	Biopsy or nephrect.	PolyC9-MA	Indir.fr.fix.fluor., imm.electr.	Igs, C3, C5	None
70	1989	Diabetic nephropathy	3	Unkn.	Unkn.	Biopsy	C6 and C9	Indir.fr.fluor.	Igs, C1q, C3, Cn, Vn	None
72	1989	Diabetic glomerulosclerosis	3	Adults	Unkn.	Unkn.	Anti-C5b-9(m)	Indir.fr.fluor.	Igs, C3, Vn	None
139	1997	Diabetic nephropathy	2	41-59	50%	Biopsy	Unkn.	Indir.fr.fix.perox.	Igs, C3, MCP	Age, sex, hyperten-sion, proteinuria, nephrotic syndrome
142	1999	Diabetic nephropathy	3	Unkn.	Unkn.	Unkn.	Unkn.	Indir.fix.fluor.	Igs, C1q, C3	Tubular basement membrane morphometry
143	2002	Diabetic nephropathy	2	Unkn.	Unkn.	Biopsy	Unkn.	Indir.fr.fix.fluor.	Igs, C1q, C3, FHR5	Glomerulosclerosis
113	2004	Diabetic nephropathy	13	45-77	42%	Biopsy	Unkn.	Indir.fix.perox.	Glycated CD59	None
		Diabetic nephropathy after transplantation	1	Unkn.	Unkn.	Biopsy	Unkn.	Unkn.	Unkn.	None
84	2004	Diabetes mellitus type 2	27	40-86	81%	Autopsy	Unkn.	Indir.fix.fr.perox.	Igs, C1q, C3, C4d, CD59, Vn, CML, αSMA, ssDNA	Diabetic glomerulosclerosis
28	2018	Diabetes mellitus type 2 with diabetic nephropathy	62	27-79[c]	66%	Biopsy	aE11	Indir.fr.perox.	C1q, MBL, FB, MASPs	Proteinuria, serum C5b-9, urine RBP and NGAL, interstitial cellular infiltration, IFTA
27	2018	Diabetes mellitus type 1 or 2 with diabetic nephropathy I, II, III, or IV	101	43-95[c]	53%	Autopsy	Unkn.	Indir.fix.perox.	C1q, C4d, MBL	Diabetic nephropathy class, IFTA
		Diabetic nephropathy	12	Unkn.	Unkn.	Biopsy	Idem	Idem	Idem	None
		Diabetes without nephropathy	58	44-94[c]	59%	Autopsy	Idem	Idem	Idem	None

**Membranous nephropathy**										
44	1983	Membranous nephropathy	3	Unkn.	Unkn.	Biopsy	PolyC9-MA	Indir.fr.fix.fluor.	Igs, C1q, C3, C4, C5, C6, C7, C8, C9	None
96	1986	Idiopathic membranous nephropathy I, II, III	6	Adults	Unkn.	Unkn.	Anti-C5b-9(m)	Indir.fr.fluor.	Igs, C1q, C3b/C3c, C3d, C3g, C5, C8, C9, FH	IFTA
		Medication-induced membranous nephropathy I, II, III, IV	9	Idem	Idem	Idem	Idem	Indir.fr.fluor., imm.electr.	Idem	IFTA
141	1986	Membranous glomerulonephritis	2	2-14[d]	Unkn.	Biopsy	Anti-C5b-9(m)	Indir.fr.fluor., indir.fix.fr.perox.	Igs, C1q, C3c, C4, fibr.	None
98	1987	Membranous glomerulonephritis	2	2-14[d]	Unkn.	Biopsy	Anti-C5b-9(m)	Indir.fr.fluor., indir.fix.fr.perox.	Igs, C1q, C3c, C4, fibr.	None
67	1987	Membranous glomerulopathy	11	Unkn.	Unkn.	Biopsy	PolyC9-MA	Indir.fr.fluor.	Igs, C3, C5, Fn, Vn	None
72	1989	Idiopathic membranous glomerulonephritis	7	Adults	Unkn.	Unkn.	Anti-C5b-9(m)	Indir.fr.fluor., imm.electr.	Igs, C3, Vn	None
70	1989	Membranous glomerulonephritis	9	Unkn.	Unkn.	Biopsy	C6 and C9	Indir.fr.fluor.	Igs, C1q, C3, Cn, Vn	None
57	1989	Membranous nephropathy	22	Unkn.	Unkn.	Biopsy	Anti-MAC	Indir.fix.perox., dir.fr.fluor.	Igs, C1q, C3, C5, C9, fibr.	Proteinuria
77	1989	Idiopathic membranous nephropathy	12	Unkn.	Unkn.	Biopsy	Unkn.	Indir.fluor.	Igs, C1q, C3, C4, Vn, HBc, HBe, HBs	Creat., proteinuria, serum C3 and C4, capsular adhesion
		Membranous nephropathy due to hepatitis B	8	Unkn.	Unkn.	Idem	Idem	Idem	Idem	Idem
152	1989	Idiopathic membranous nephropathy I/II, IV	2	5-15	100%	Biopsy	PolyC9-MA	Indir.fix.perox., imm.electr.	IgG, C3	Age, sex, proteinuria, serum HBe and HBs
		Membranous nephropathy II due to hepatitis B	6	3-13	33%	Idem	Idem	Idem	IgG, C3, HBe, HBs	None
81	1991	Idiopathic membranous nephropathy	18	Unkn.	Unkn.	Biopsy	PolyC9-MA	Indir.fr.fix.fluor.	Igs, C1q, C3, C4, CD59, CD45, fibr.	None
75	1993	Idiopathic membranous nephropathy	2	Unkn.	Unkn.	Biopsy	PolyC9-MA	Indir.fr.fix.fluor.	Igs, C3c, C3d, C5, C9, Vn, Vn receptor	None

79	1994	Membranous nephropathy II, IV	6	47-65	33%	Biopsy	A239	Indir.fr.fix.perox. imm.electr.	Vn, Vn receptor	None
82	1995	Idiopathic membranous glomerulonephritis	5	Unkn.	Unkn.	Biopsy	C9	Indir.fr.fix.fluor.	C3b/C3c, CD59	None
139	1997	Membranous nephropathy I, II	7	23-70	29%	Biopsy	Unkn.	Indir.fr.fix.perox.	Igs, C3, MCP	Age, sex, protein-uria, hematuria, nephrotic or nephritic syndr.
142	1999	Membranous nephropathy	3	Unkn.	Unkn.	Unkn.	Unkn.	Indir.fix.fluor.	Igs, C1q, C3	Tubular basement membrane morphometry
112	2002	Idiopathic membranous nephropathy I, II, III, IV	35	23-71	66%	Biopsy	aE11	Indir.fr.fix.perox.	CD3, CD68, HLA-DR, α3β1, LFA1β, ICAM1	Creat., proteinuria, nephrotic syndr., glomerular hypercellularity, interstitial inflammation, IFTA
143	2002	Membranous nephropathy	10	Unkn.	Unkn.	Biopsy	Unkn.	Indir.fr.fix.fluor.	Igs, C1q, C3, FHR5	None
148	2004	Idiopathic membranous nephropathy I, II, III, IV	20	44-57[c]	70%	Biopsy	aE11	Indir.fr.perox.	C1q, C3b/C3c, FH	None
149	2004	Lupus-like membra-nous nephropathy II with C4 deficiency	1	Unkn.	100%	Biopsy	Unkn.	Unkn.	Igs, C1q, C3, C4	Not applicable
80	2006	Idiopathic membranous glomerulonephritis	60	31-86[c]	63%	Biopsy	Unkn.	Indir.fr.fix.fluor.	Cn, phosphorylated PKC	None
156	2010	Idiopathic membranous nephropathy I, II, III	24	28-75	67%	Biopsy	aE11	Indir.fix.fluor.	Igs, C1q, C3, aldose reductase, super-oxide dismutase 2	None
87	2010	Idiopathic segmental or diffuse membranous nephropathy I, II, III	16	2-23	Unkn.	Biopsy	Unkn.	Indir.fr.fluor.	Igs, C1q, C3c, C4d, MBL, FB, CD59, C4bp	Blood pressure, creat., proteinuria, hematuria, nephro-tic syndr., mesan-gial hypercellularity, treatment effect
150	2011	Primary membranous nephropathy I, II, III	8	39-77	38%	Biopsy	aE11	Indir.fr.fluor., imm.electr.	Igs, C1q, C3, α-enolase, fibr.	None

151	2011	Membranous nephropathy	Unkn.	Unkn.	Unkn.	Biopsy	Unkn.	Indir.fluor.	Igs, C3c	None
147	2012	PLA2R-related membranous nephropathy after transplantation	1	56	100%	Biopsy	Unkn.	Indir.fluor.	Igs, C1q, C3, MBL, PLA2R	Not applicable
157	2014	Membranous nephropathy due to treatment	1	6	100%	Biopsy	Unkn.	Unkn.	Igs, C1q, C3, PLA2R	Not applicable
153	2014	Membranous glomerulopathy	Unkn.	Unkn.	Unkn.	Unkn.	aE11	Indir.fix.imm.hist.	Igs, C1q, C3, C4d	None
154	2015	Alloimmune antenatal NEP-related membra-nous nephropathy	1	0	0%	Biopsy	Unkn.	Fluor.	Igs, C1q, C3, NEP	Not applicable
155	2016	PLA2R-related mem-branous nephropathy with MBL deficiency	1	25	100%	Biopsy	Unkn.	Unkn.	Igs, C1q, C3, C4, FB, P, PLA2R	Not applicable
123	2017	Idiopathic membranous nephropathy	17	1-82[c]	65%	Biopsy	Unkn.	Indir.fix.fluor.	MBL, LC3, p62, synaptopodin	None
18	2019	Idiopathic membranous nephropathy	5	Unkn.	Unkn.	Unkn.	aE11	Indir.fix.perox.	Igs, C3b/C3c, C3d, C9, CD68	None
**IgA nephropathy and IgA vasculitis with nephritis**										
44	1983	IgA nephropathy	3	Unkn.	Unkn.	Biopsy	PolyC9-MA	Indir.fr.fix.fluor.	Igs, C1q, C3, C4, C5, C6, C7, C8, C9	None
96	1986	Idiopathic IgA nephropathy	7	Adults	Unkn.	Unkn.	Anti-C5b-9(m)	Indir.fr.fluor.	Igs, C1q, C3b/C3c, C3d, C3g, C5, C8, C9, FH	IFTA
		Schönlein-Henoch nephropathy	2	Idem	Idem	Idem	Idem	Idem	Idem	Idem
137	1987	Primary IgA nephropathy	12	Unkn.	Unkn.	Open biopsy	PolyC9-MA	Indir.fr.fix.fluor.	IgA, C3	Age, sex, hypertension, creat., histological lesions
67	1987	IgA nephropathy	11	Unkn.	Unkn.	Biopsy	PolyC9-MA	Indir.fr.fluor.	Igs, C3, C5, Fn, Vn	None

52	1987	IgA mesangial glomerulonephritis	23	6-56	78%	Biopsy	Anti-MAC-neo	Indir.fr.fix.fluor.	Igs, C1q, C3b/C3c, C3d, C4, C5, C6, C9, FB, P, FH, Fn, Vn, fibr.	Age, sex, blood pressure, creat., proteinuria, hema-turia, histological lesions
		Henoch-Schönlein purpura nephritis	2	9-10	50%	Idem	Idem	Idem	Idem	Idem
76	1987	Primary IgA nephropathy	15	Unkn.	Unkn.	Open biopsy	C5 and C9	Dir.fr.fluor.	Igs, C1q, C3, C4, Vn	Creat., serum Igs, C3, and C4, glomerulosclerosis, crescents, capsular adhesion
97	1988	Primary IgA nephropathy	30	4-17	70%	Biopsy	PolyC9-MA	Indir.fluor., imm.electr.	Igs, C3, C4, C5, C9	Disease duration, proteinuria, hematuria, crescents, IFTA
70	1989	IgA nephropathy	16	Unkn.	Unkn.	Biopsy	C6 and C9	Indir.fr.fluor.	Igs, C1q, C3, Cn, Vn	None
72	1989	Idiopathic mesangial IgA nephropathy	10	Adults	Unkn.	Unkn.	Anti-C5b-9(m)	Indir.fr.fluor., imm.electr.	Igs, C3, Vn	None
57	1989	IgA nephropathy	75	Unkn.	Unkn.	Biopsy	Anti-MAC	Indir.fix.perox., dir.fr.fluor.	Igs, C1q, C3, C5, C9, fibr.	Mesangial hypercellularity, glomerulosclerosis
61	1990	Henoch-Schönlein purpura nephritis	4	Unkn.	Unkn.	Biopsy	Xia 1988	Indir.fr.fluor.	Igs, C3c, C5, C6, C7, C8, C9, Vn	None
81	1991	IgA nephropathy	31	Unkn.	Unkn.	Biopsy	PolyC9-MA	Indir.fr.fix.fluor.	Igs, C1q, C3, C4, CD59, CD45, fibr.	None
162	1991	IgA nephropathy	14	Unkn.	Unkn.	Unkn.	Unkn.	Indir.fluor.	IgA, polymorpho-nuclear cells, super-oxide dismutase	Advance stage
93	1991	IgA nephropathy with C3 deficiency	1	23	100%	Open biopsy	PolyC9-MA	Unkn.	Igs, C1q, C4, C5, C9	Not applicable
78	1992	IgA nephropathy with C9 deficiency	2	8-9	100%	Biopsy	PolyC9-MA	Indir.fr.fluor.	Igs, C3, C4, C5, C8, C9, Vn	Disease duration, blood pressure, proteinuria, hematuria, disease progression

75	1993	IgA nephropathy	22	Unkn.	Unkn.	Biopsy	PolyC9-MA	Indir.fr.fix.fluor.	Igs, C3c, C3d, C5, C9, Vn, Vn receptor	None
		Henoch-Schönlein purpura nephritis	7	Unkn.	Unkn.	Idem	Idem	Idem	Idem	None
110	1995	Primary IgA nephropathy	20	19-45	60%	Biopsy	aE11	Indir.fr.fix.perox.	Igs, C3, CD3, CD68, HLA-DR	Age, creat., protein-uria, hematuria, interstitial inflam-mation, IFTA, dis-ease progression
163	1995	IgA nephropathy	54	Children	61%	Biopsy	Unkn.	Unkn.	Vn	Proteinuria, nephrotic syndr., mesangial expansion and hypercellularity
58	1995	IgA nephropathy	2	Unkn.	Unkn.	Biopsy	aE11, anti-C5b-9(m), B7	Indir.fr.fix.perox.	C1q, C1r, C1s, C1 inh., C3c, C3d, C4c, C5, C7, C9, Cn, Vn, CD59, protease nexin	None
139	1997	IgA nephropathy	11	15-45	91%	Biopsy	Unkn.	Indir.fr.fix.perox.	Igs, C3, MCP	Age, sex, protein-uria, hematuria, nephritic syndrome
		Henoch-Schönlein purpura	2	6-9	50%	Idem	Idem	Idem	Idem	Age, sex, hematuria
161	1997	IgA nephropathy	120	7-53	42%	Biopsy	Unkn.	Indir.fix.perox.	Igs, C1q, C3c, CD3, CD11, CD15, CD31, CD45, CD68, HLA-DR, fibr.	None
160	1998	Primary IgA nephropathy	45	15-48	47%	Biopsy	aE11	Indir.fix.fr.perox.	Igs, C1q, C3b/C3c, C4c, MBL, MASP1, P, fibr.	None
142	1999	IgA nephritis	8	Unkn.	Unkn.	Unkn.	Unkn.	Indir.fix. fluor.	Igs, C1q, C3	Tubular basement membrane morphometry
99	2000	Henoch-Schönlein purpura nephritis	10	9-23	40%	Biopsy	aE11	Indir.fix.fr.perox.	C3b/C3c, MBL, MASP1, C4bp	Age, sex, disease duration, creat., proteinuria, hema-turia, serum IgA, histological lesions

128	2001	IgA nephropathy	14	20-51	43%	Open biopsy	aE11	Indir.fix.fr.perox.	IgA, C3, C3d	Age, sex, creat., proteinuria, hematuria, serum IgA, C3 mRNA expression
143	2002	IgA nephropathy	20	Unkn.	Unkn.	Biopsy	Unkn.	Indir.fr.fix.fluor.	Igs, C1q, C3, FHR5	None
85	2005	Henoch-Schönlein purpura nephritis	31	4-18	Unkn.	Biopsy	Unkn.	Indir.fr.perox.	Igs, C1q, C3c, C4, MBL, MASP1, FB, CD59, C4bp, fibr.	Proteinuria, hematuria, mesangial expansion, mesangial and endocapillary hypercellularity, crescents, caps. adhesion, glomerulosclerosis, IFTA
115	2008	IgA nephropathy	30	17-67[d]	75%[d]	Biopsy	aE11	Indir.fix.perox.	Igs, C1q, C3, C4, CD68, αSMA, α3β1-integrin, TGFβ1	Creat., interstitial inflammation, disease progression
151	2011	IgA nephropathy with C9 deficiency	1	44	100%	Biopsy	Unkn.	Indir.fluor.	Igs, C3c	Not applicable
164	2010	IgA nephropathy with >1 g/d proteinuria but normal kidney function	35	27-39[c]	60%	Biopsy	Unkn.	Indir.fix.fr.perox.	Igs, C1q, C3, CR1, p27, WT1, nestin, laminin, fibr.	None
159	2014	IgA nephropathy	1	16	100%	Biopsy	Unkn.	Unkn.	Igs, C3	Not applicable
130	2015	IgA nephropathy with TMA	1	32	100%	Biopsy	A239	Indir.fix.perox.	C1q, C4d, MBL	Not applicable
165	2017	Idiopathic IgA nephropathy	96	4-66[c]	65%	Biopsy	aE11	Indir.fix.perox.	Igs, C1q, C3, C4d, MBL, P, fibr.	None
124	2017	IgA nephropathy	25	0-63[c]	80%	Biopsy	aE11	Indir.fix.perox., liquid chromatography and mass spectrometry	C1q, C3c	Blood pressure, eGFR, proteinuria, proteomics, histological lesions, disease progression
158	2018	IgA nephropathy	36	18-84[d]	66%[d]	Biopsy	Unkn.	Fix.perox.	C3b/C3c, C3d, C4d, FH, FHR1, FHR5	Disease progression

167	2019	IgA nephropathy or IgA vasculitis with nephritis	116	0-84	69%	Biopsy	A239	Indir.fix.perox.	C1q, C4d, MBL, FB	Microangiopathy
19	2020	IgA nephropathy or Henoch-Schönlein purpura nephritis	67	3-17	61%	Biopsy	Unkn.	Indir.fix.perox.	Igs, C1q, C3	Age, sex, hyper-tension, creat., proteinuria, hematuria, nephrotic syndr., histological lesions, treatment, disease progression
166	2020	IgA nephropathy	132	17-82	54%	Biopsy	Unkn.	Dir.fr.fluor.	Igs, C1q, C3, C4, C4d, MBL, MASPs, FB, P	Crescents
**Lupus nephritis**										
95	1981	SLE nephritis II, III, IV, V	22	Unkn.	Unkn.	Autopsy or biopsy	Kolb 1975	Dir.fr.fluor., imm.electr.	Igs, C1q, C3, C9	Interstitial inflam-mation, IFTA
44	1983	SLE nephritis	3	Unkn.	Unkn.	Biopsy	PolyC9-MA	Indir.fr.fix.fluor.	Igs, C1q, C3, C4, C5, C6, C7, C8, C9	None
46	1985	SLE with glomerulo-nephritis	1	18	0%	Unkn.	aE11	Indir.fluor.	Igs, C3	Not applicable
96	1986	SLE nephropathy II, IV	9	Adults	Unkn.	Biopsy	Anti-C5b-9(m)	Indir.fr.fluor., imm.electr.	Igs, C1q, C3b/C3c, C3d, C3g, C5, C8, C9, FH	IFTA
67	1987	Proliferative systemic lupus nephritis	8	Unkn.	Unkn.	Biopsy	PolyC9-MA	Indir.fr.fluor.	Igs, C3, C5, Fn, Vn	None
72	1989	SLE nephritis II, III, IV	7	Adults	Unkn.	Unkn.	Anti-C5b-9(m)	Indir.fr.fluor.	Igs, C3, Vn	None
70	1989	SLE nephritis	10	Unkn.	Unkn.	Biopsy	C6 and C9	Indir.fr.fluor.	Igs, C1q, C3, Cn, Vn	None
77	1989	Lupus nephritis V	8	Unkn.	Unkn.	Biopsy	Unkn.	Indir.fluor.	Igs, C1q, C3, C4, Vn	Creat., proteinuria, serum C3 and C4, caps. adhesion
152	1989	SLE-associated mem-branous nephropathy	1	18	0%	Biopsy	PolyC9-MA	Indir.fix.perox., imm.electr.	IgG, C3	Not applicable

57	1989	SLE	20	Unkn.	Unkn.	Biopsy	Anti-MAC	Indir.fix.perox., dir.fr.fluor.	Igs, C1q, C3, C5, C9, fibr.	Serum C4
81	1991	Diffuse lupus nephritis	9	Unkn.	Unkn.	Biopsy	PolyC9-MA	Indir.fr.fix.fluor.	Igs, C1q, C3, C4, CD59, CD45, fibr.	None
75	1993	Lupus nephritis IV	4	Unkn.	Unkn.	Biopsy	PolyC9-MA	Indir.fr.fix.fluor.	Igs, C3c, C3d, C5, C9, Vn, Vn receptor	None
58	1995	Lupus nephritis IV	2	Unkn.	Unkn.	Biopsy	aE11, anti-C5b-9(m), B7	Indir.fr.fix.perox.	C1q, C1r, C1s, C1 inh., C3c, C3d, C4c, C5, C7, C9, Cn, Vn, CD59, protease nexin	None
111	1996	Lupus nephritis II, III, IV	15	Unkn.	Unkn.	Biopsy	WU-7,2	Indir.fr.fix.perox.	CR1	Histological lesions, histological activity index
139	1997	Lupus nephritis III, V	2	19-30	50%	Biopsy	Unkn.	Indir.fr.fix.perox.	Igs, C3, MCP	Age, sex, nephrotic or nephritic syndr.
142	1999	Lupus nephritis IV	5	Unkn.	Unkn.	Unkn.	Unkn.	Indir.fix.fluor.	Igs, C1q, C3	Tubular basement membrane morphometry
143	2002	Lupus nephritis	7	Unkn.	Unkn.	Biopsy	Unkn.	Indir.fr.fix.fluor.	Igs, C1q, C3, FHR5	None
172	2008	Lupus nephritis V	1	27	0%	Biopsy	aE11	Indir.fr.fluor., indir.fix.perox., imm.electr.	Igs, C1q, C3, C4, vimentin	Not applicable
170	2008	Lupus nephritis II	1	30	0%	Biopsy	Unkn.	Indir.fluor.	Igs, C1q, C3c, C4	Treatment effect
151	2011	SLE	Unkn.	Unkn.	Unkn.	Biopsy	Unkn.	Indir.fluor.	Igs, C3c	None
175	2012	TMA due to SLE with antiphospholipid syndrome	1	27	0%	Biopsy	Unkn.	Fluor.	C3, C4d	Not applicable
119	2013	Lupus nephritis II, III, IV, V	11	23-41	27%	Biopsy	WU-13,15	Indir.fr.fluor.	Igs, C1q, C3, MBL, ficolin-2	None
130	2015	SLE with TMA	8	17-49	0%	Autopsy or biopsy	A239	Indir.fix.perox.	C1q, C4d, MBL	None
173	2017	Lupus nephritis II, III, IV, V, VI	222	10-56[c]	16%	Biopsy	Unkn.	Indir.fr.fix.fluor.	Igs, C1q, C3b, FBb, fibr.	None
174	2018	Lupus nephritis II, III, IV, V	38	Unkn.	Unkn.	Biopsy	Unkn.	Unkn.	C5a	Chronicity index
133	2018	Active lupus nephritis	5	5-18[d]	13%	Biopsy	Unkn.	Fluor.	None	None

171	2018	Lupus nephritis II, III, IV, V	30	8-59[c]	20%	Biopsy	X197	Indir.fix.perox.	Igs, C1q, C3, C4, fibr.	Age, race, SLEDAI, blood pressure, medication, Hb, albumin, creat., proteinuria, hematuria, serum C3, C4, and anti-dsDNA, activity and chronicity indices, glomerulosclerosis, crescents, IFTA, treatment effect
18	2019	Lupus nephritis III, IV, V	51	18-71	18%	Biopsy	aE11	Indir.fix.perox.	Igs, C3b/C3c, C3d, C9, CD68	Serum C3, C4, and anti-dsDNA, activity and chronicity indices, treatment effect
**Membranoproliferative glomerulonephritis, C3 glomerulopathy, and postinfectious glomerulonephritis**										
44	1983	MPGN I	3	Unkn.	Unkn.	Biopsy	PolyC9-MA	Indir.fr.fix.fluor.	Igs, C1q, C3, C4, C5, C6, C7, C8, C9	None
		MPGN II	2	Unkn.	Unkn.	Nephrect.	Idem	Idem	Idem	Idem
183	1984	Acute poststreptococcal glomerulonephritis	11	5-14	45%	Biopsy	PolyC9-MA	Indir.fr.fluor.	C3, C5, immune cells	Disease duration
96	1986	MPGN I	9	Adults	Unkn.	Biopsy	Anti-C5b-9(m)	Indir.fr.fluor., imm.electr.	Igs, C1q, C3b/C3c, C3d, C3g, C5, C8, C9, FH	None
141	1986	MPGN	2	2-14[d]	Unkn.	Biopsy	Anti-C5b-9(m)	Indir.fr.fluor., indir.fix. fr.perox.	Igs, C1q, C3c, C4, fibr.	Treatment effect
98	1987	MPGN	2	2-14[d]	Unkn.	Biopsy	Anti-C5b-9(m)	Indir.fr.fluor., indir.fix. fr.perox.	Igs, C1q, C3c, C4, fibr.	None
67	1987	MPGN I	3	Unkn.	Unkn.	Biopsy	PolyC9-MA	Indir.fr.fluor.	Igs, C3, C5, Fn, Vn	None
		Postinfectious glomerulonephritis	4	Idem	Idem	Idem	Idem	Idem	Idem	Idem
126	1987	MPGN II	3	Unkn.	Unkn.	Biopsy	PolyC9-MA	Indir.fr.fix.fluor., imm.electr.	Igs, C3, C5	None
72	1989	MPGN I	5	Adults	Unkn.	Unkn.	Anti-C5b-9(m)	Indir.fr.fluor.	Igs, C3, Vn	None

57	1989	MPGN I	11	Unkn.	Unkn.	Biopsy	Anti-MAC	Indir.fix.perox., dir.fr.fluor.	Igs, C1q, C3, C5, C9, fibr.	Not applicable
		MPGN II	1	Idem	Idem	Idem	Idem	Idem	Idem	Not applicable
75	1993	MPGN I	1	Unkn.	Unkn.	Biopsy	PolyC9-MA	Indir.fr.fix.fluor.	Igs, C3c, C3d, C5, C9, Vn, Vn receptor	Not applicable
		MPGN II	2	Idem	Idem	Idem	Idem	Idem	Idem	None
		MPGN III	1	Idem	Idem	Idem	Idem	Idem	Idem	Not applicable
184	1994	Acute poststreptococcal glomerulonephritis	5	3-17[d]	80%[d]	Biopsy	Unkn.	Indir.fr.fix.fluor.	Igs, C1q, C3, P, Vn	None
139	1997	MPGN I	4	20-49	100%	Biopsy	Unkn.	Indir.fr. fix.perox.	Igs, C3, MCP	Age, sex, nephrotic or nephritic syndr.
143	2002	MPGN I	1	Unkn.	Unkn.	Biopsy	Unkn.	Indir.fr.fix.fluor.	Igs, C1q, C3, FHR5	Not applicable
		Postinfectious glomerulonephritis	2	Unkn.	Unkn.	Biopsy	Unkn.	Indir.fr.fix.fluor.	Igs, C1q, C3, FHR5	None
114	2006	MPGN I	18	Children	56%[d]	Biopsy	1B4	Dir.fr.fix.perox.	None	Serum C5b-9
86	2007	Acute poststreptococcal glomerulonephritis	18	4-23	Unkn.	Biopsy	Unkn.	Indir.fr.fluor.	Igs, C1q, C3c, C4, MBL, MASP1, FB CD59, C4bp, fibr.	Age, disease duration, blood pressure, creat., proteinuria, hema-turia, histological lesions, disease progression
179	2009	C3 glomerulonephritis	2	7-12	0%	Biopsy	Unkn.	Fix.perox.	C3	None
90	2009	Dense deposit disease	8	11-49	63%	Biopsy	Not applicable	Liquid chromato-graphy and mass spectrometry	Not applicable	Age, sex, creat., proteinuria, hema-turia, serum C3 and C4, histological lesions, crescents
		Immune complex-mediated MPGN	9	Unkn.	Unkn.	Idem	Idem	Idem	Idem	None
180	2011	C3 glomerulonephritis	2	38-73	100%	Biopsy	Not applicable	Idem	Not applicable	Age, sex, edema, hypertension, creat., proteinuria, hematuria, serum C3 and C4, histological lesions

91	2012	C3 glomerulonephritis	8	8-73[d]	50%[d]	Biopsy	Not applicable	Idem	Not applicable	Age, sex, creat., proteinuria, hema-turia, serum C3 and C4, histological lesions, disease progression
		Dense deposit dis.	1	Unkn.	Unkn.	Idem	Idem	Idem	Idem	Not applicable
105	2012	Dense deposit dis.	1	17	Unkn.	Biopsy	Unkn.	Fluor.	C3	Not applicable
103	2012	C3 glomerulonephritis	2	20-42[d]	100%	Biopsy	aE11	Indir.fluor.	Igs, C1q, C3	Histological lesions, disease progression
		Dense deposit dis.	2	20-42[d]	100%	Idem	Idem	Idem	Idem	Idem
181	2014	Dense deposit dis.	3	8-28	33%	Biopsy	Unkn.	Fluor.	Igs, C1q, C3	Age, sex, eGFR, histological lesions, disease progression
106	2015	Rapid progressive C3 glomerulopathy	3	27-63	33%	Biopsy	Unkn.	Perox.	Igs, C3	Treatment effect
107	2015	C3 glomerulonephritis	1	5	100%	Biopsy	Unkn.	Unkn.	C3	Not applicable
182	2016	C3 glomerulopathy due to CFHR5 mutation	1	28	100%	Nephrect.	Unkn.	Indir.fr.fix.	C3c, P, FHR5	Not applicable
92	2017	Dense deposit dis.	6	11-25	50%	Biopsy	Not applicable	Liquid chromato-graphy and mass spectrometry	Not applicable	None
		C3 glomerulonephritis	6	16-61	50%	Idem	Idem	Idem	Idem	None
101	2018	Dense deposit dis.	3	7-12	0%	Biopsy	Unkn.	Indir.fix.fluor.	C3c	Disease duration, drusen, creat., proteinuria, hematuria, serum renin, C3, C3a, C5, C5a, and C5b-9, GBM thickness
102	2019	C3 glomerulopathy	24	9-74[d]	52%[d]	Biopsy	aE11	Indir.fix.perox.	Igs C1q, C3b/ C3c, C3d, C4d, P, FH, FHR1, FHR5, CD68	eGFR, proteinuria, histological lesions, disease progression
		Recurrent C3 glomerulopathy in kidney transplant	8	Idem	Idem	Biopsy	Idem	Idem	Idem	Idem
108	2020	Immune complex-mediated MPGN	2	14-15	50%	Biopsy	Unkn.	Indir.fr.fix.fluor.	Igs, C1q, C3c	Treatment effect

**Thrombotic microangiopathy**									
75	1993	Hemolytic uremic syndr.	1	Unkn.	Unkn.	Biopsy	PolyC9-MA	Indir.fr.fix.fluor.	Igs, C3c, C3d, C5, C9, Vn, Vn receptor	Not applicable
103	2012	aHUS	1	Unkn.	Unkn.	Biopsy	aE11	Indir.fluor.	Igs, C1q, C3	Not applicable
190	2013	STEC-HUS	1	26	0%	Biopsy	WU-13,15	Indir.fix.perox.	C3	Not applicable
120	2013	Congenital TTP	2	2-4	0%	Biopsy or unkn.	Unkn.	Indir.fix.perox.	C3	ADAMTS13 mutations
153	2014	STEC-HUS	10	22-44[d]	18%[d]	Biopsy	aE11	Indir.fix. imm.hist.	Igs, C1q, C3, C4d, CD3, CD44, CD61, CD68, caspase 3, Ki67, aquaporin, β-catenin, fibr.	None
130	2015	aHUS	11	22-77	18%	Autopsy or biopsy	A239	Indir.fix.perox.	C1q, C4d, MBL	Idem
		STEC-HUS	1	14	100%	Biopsy	Idem	Idem	Idem	Idem
		TMA after hematopoietic stem cell transplantation	6	18-54	50%	Autopsy or biopsy	Idem	Idem	Idem	Idem
		Drug toxicity in kidney transplant	2	40-47	0%	Biopsy	Idem	Idem	Idem	Idem
		Recurrent aHUS in kidney transplant	3	6-37	67%	Biopsy or nephrect.	Idem	Idem	Idem	Idem
188	2015	aHUS	1	16	100%	Biopsy	aE11	Perox.	None	Not applicable
122	2016	STEC-HUS	1	2	100%	Biopsy	Unkn.	Fluor.	None	Not applicable
192	2017	Hypertension-associated TMA	7	29-65	38%	Biopsy	Unkn.	Indir.fr.fluor.	Igs, C3c, C4d	Age, sex, blood pressure, Hb, thrombocytes, creat., proteinuria, complement activity, serum C5b-9, complement mutations, end-stage kidney disease, treatment effect, recurrence after transplantation
		Idem after transplantation	2	38	50%	Idem	Idem	Idem	Idem	Idem

132	2018	Congenital TTP	5	9-40	60%	Biopsy	aE11	Indir.fix.perox.	Igs, C3, C4d, ADAMTS13	Age, sex, creat., proteinuria, serum C3 and C4, ADAMTS13 mutations, glomerulosclerosis, arteriosclerosis, IFTA
		Acquired TTP	8	22-66	Unkn.	Autopsy or biopsy	Idem	Idem	Idem	None
144	2018	Hypertension-associated TMA with deposition of C5b-9 on cultured endothelial cells	10	23-72	57%	Biopsy	Unkn.	Indir.fr.fluor.	Igs, C3c	None
100	2019	TMA treated with eculizumab	6	22-65	50%	Biopsy	aE11	Indir.fr.fluor.	Igs	Medication, creat., serum C3 and C4, CH50, complement mutations, active or chronic TMA, treatment effect
		TMA treated with eculizumab in kidney transplant	7	32-72	29%	Idem	Idem	Idem	Idem	Idem
193	2020	TMA and hypertensive emergency	15	17-61[cd]	58%[d]	Biopsy	Unkn.	Indir.fr.fluor.	IgM, C3c	None
**ANCA-associated vasculitis**										
139	1997	Wegener's granulomatosis	1	44	0%	Biopsy	Unkn.	Indir.fr.fix.perox.	Igs, C3, MCP	Not applicable
143	2002	ANCA-associated glomerulonephritis	6	Unkn.	Unkn.	Biopsy	Unkn.	Indir.fr.fix.fluor.	Igs, C1q, C3, FHR5	None
195	2006	Idiopathic focal pauci-immune crescentic necrotizing glomerulonephritis	34	25-80	50%	Biopsy	aE11	Indir.fr.perox.	HLA-DR, TGFβ1, PCNA, αSMA, ICAM1, monocytes/ macrophages	Creat.
196	2008	Idem	34	>15	50%	Idem	Idem	Idem	Idem	Idem

116	2009	ANCA-associated vasculitis with pauci-immune crescentic necrotizing glomerulonephritis	7	51-75	71%	Biopsy	Unkn.	Indir.fr.fix.perox.	Igs, C1q, C3c, C3d, C4d, MBL, FB, P	Creat., histological lesions, crescents
117	2010	ANCA-negative pauci-immune crescentic glomerulonephritis	12	21-70	50%	Biopsy	Unkn.	Indir.fr.fix.fluor.	Igs, C1q, C3c, C3d, C4d, MBL	Age, sex, Hb, creat., proteinuria, serum C3, crescents, disease progression
22	2013	Active ANCA-associated glomerulonephritis	29	28-89[c]	41%	Biopsy	Unkn.	Indir.fix.perox.	Igs, C3d, FBb	Proteinuria, crescents, IFTA
53	2015	Renal ANCA-associated vasculitis	25	35-95[cd]	63%[d]	Biopsy	Ab55811	Indir.fix.perox.	Igs, C3c, C4d, FBb, fibr.	Birmingham vasculitis activity score, eGFR, proteinuria, serum C5b-9, disease progression
130	2015	Granulomatosis with polyangiitis with TMA	1	35	100%	Biopsy	A239	Indir.fix.perox.	C1q, C4d, MBL	Not applicable
194	2017	ANCA-associated crescentic and/or necrotizing glomerulonephritis	49	32-90[cd]	70%[d]	Biopsy	Unkn.	Indir.fr.fluor.	Igs, C1q, C3c, C3d, C4d, MBL	Creat., proteinuria, histological class, crescents, IFTA
**Interstitial nephritis**										
96	1986	Medication-induced acute tubulointerstitial nephritis	1	Adult	Unkn.	Biopsy	Anti-C5b-9(m)	Indir.fr.fluor.	Igs, C1q, C3b/C3c, C3d, C3g, C5, C8, C9, FH	Not applicable
70	1989	Interstitial nephritis	2	Unkn.	Unkn.	Biopsy	C6 and C9	Indir.fr.fluor.	Igs, C1q, C3, Cn, Vn	None
139	1997	Minimal change, membranous, IgA, or diabetic nephropathy, mesangio- or membranoproliferative glomerulonephritis, lupus or interstitial nephritis, granulomato-	46	3-70	61%	Biopsy	Unkn.	Indir.fr.fix.perox.	Igs, C3, MCP	Interstitial mononuclear infiltration, relative interstitial volume

		sis with polyangiitis, nail-patella syndrome, thin membrane disease, FSGS							
143	2002	Interstitial nephritis	3	Unkn.	Unkn.	Biopsy	Unkn.	Indir.fr.fix.fluor.	Igs, C1q, C3, FHR5	None
118	2011	Juvenile nephrophthisis	16	Unkn.	Unkn.	Biopsy	Unkn.	Unkn.	Igs, C3, TINag, collagen, nephro-cystin 1, Fas antigen	None
199	2011	IgG4-related tubulointerstitial nephritis	1	59	100%	Biopsy	Unkn.	Fluor.	Igs, C1q, C3c, C4d, MBL, ficolin	Not applicable
31	2019	Acute tubulointerstitial nephritis	54	18-76[c]	43%	Biopsy	aE11	Indir.fluor.	KIM1	None
**Acute tubular necrosis**									
203	1993	Hemorrhagic cystitis due to adenovirus type 11 after bone marrow transplantation	1	21	100%	Biopsy	PolyC9-MA	Indir.fr.fluor.	Igs, C1q, C3, C4, fibr., adenovirus	Not applicable
135	1993	Minimal change, membranous, IgA, or diabetic nephropathy, lupus nephritis, diffuse mesangial proliferative glomerulonephritis, FSGS	31	Unkn.	Unkn.	Biopsy	Unkn.	Indir.fr.fix.fluor.	Lysozyme, α1-antitrypsin, α1-antichymotrypsin	Tubular atrophy or necrosis
94	1995	Idem	45	Unkn.	Unkn.	Unkn.	Unkn.	Indir.fr.fix.fluor.	Igs, C1q, C3c, C3d, C4, C5, C6, C7, C8, C9	Tubular atrophy or necrosis
142	1999	Idem	21	Unkn.	Unkn.	Unkn.	Unkn.	Indir.fix.fluor.	Igs, C1q, C3	Tubular basement membrane morphometry
200	2005	Acute tubular necrosis	4	20-80[d]	Unkn.	Biopsy	C8	Indir.fr.fix.fluor.	C3d, C4d	None
136	2018	Acute tubular necrosis due to sepsis, ischemia-reperfusion, delayed graft function, or medication	21	35-93[cd]	62%[d]	Autopsy	Unkn.	Indir.fix.perox.	FH	None

31	2019	Nephrotoxic acute tubular necrosis	5	25-65	60%	Biopsy	aE11	Indir.fluor.	KIM1	None
125	2020	COVID19 with eGFR <90 ml/min or decline in eGFR by ≥30%	6	51-86	67%	Autopsy	aE11	Indir.fix.perox.	CD8, CD56, CD68, SARS-CoV-2 nucleocaspid protein	Histological lesions, IFTA
**Reflux nephropathy**										
44	1983	Obstructive uropathy	3	Unkn.	Unkn.	Biopsy or nephrect.	PolyC9-MA	Indir.fr.fix.fluor.	Igs, C1q, C3, C4, C5, C6, C7, C8, C9	None
127	1987	Vesicoureteral reflux	8	8-51	13%	Biopsy or nephrect.	PolyC9-MA	Indir.fr.fluor.	Igs, C1q, C3, C4, C5, C9, P, Fn, vimentin, collagen, laminin, cytokeratin, uromodulin	None
75	1993	Reflux nephropathy	1	Unkn.	Unkn.	Biopsy	PolyC9-MA	Indir.fr.fix.fluor.	Igs, C3c, C3d, C5, C9, Vn, Vn receptor	Not applicable
**Kidney tumors**										
210	1996	Renal cell carcinoma	22	39-82	59%	Biopsy	Unkn.	Indir.fr.fix.perox.	Igs, C1q, C3, CD59, DAF, MCP, MHC I, MHC II, immune cells, vimentin, cytokeratin	None
138	1996	Renal cell carcinoma	10	Unkn.	Unkn.	Unkn.	PolyC9-MA	Indir.fr.fix.perox.	C3, CD59, DAF, MCP	None
209	2000	Renal cell carcinoma	31	Unkn.	Unkn.	Nephrect.	aE11	Indir.fr.fix.fluor., indir.fr.fix.perox.	C3d, CD59, DAF, MCP, CD45, G250, asparagus	None
207	2020	Clear cell renal cell carcinoma	20	37-81[c]	57%	Nephrect.	aE11	Indir.fr.fluor.	C1q, MBL, CD59, C3aR, C5R1, PTX3	None
**Kidney transplantation**										
96	1986	Acute rejection	2	Adults	Unkn.	Biopsy	Anti-C5b-9(m)	Indir.fr.fluor.	Igs, C1q, C3b/C3c, C3d, C3g, C5, C8, C9, FH	None

214	1986	*De novo* membranous nephropathy	13	R: 10-43	R: 38%	Biopsy	Anti-C5b-9(m)	Indir.fr.fluor.	Igs, C1q, C3b/C3c, C3d, FH	None
67	1987	Acute rejection	4	Unkn.	Unkn.	Biopsy	PolyC9-MA	Indir.fr.fluor.	Igs, C3, C5, Fn, Vn	None
70	1989	Rejection	14	Unkn.	Unkn.	Biopsy	C6 and C9	Indir.fr.fluor.	Igs, C1q, C3, Cn, Vn	None
143	2002	Interstitial rejection	4	Unkn.	Unkn.	Biopsy	Unkn.	Indir.fr.fix.fluor.	Igs, C1q, C3, FHR5	None
129	2003	Protocol biopsy one week after transplantation	37	D: 21-72<br>R: 20-77	R: 57%	Biopsy	aE11	Indir.fr.fix.fluor.	C1q, C3c, C4d, C6, MBL, MASP1, FB	None
83	2004	Acute rejection in three months after transplantation, living donors	10	D: 37-79[c]<br>R: 21-44[c]	D: 50%<br>R: 40%	Biopsy	Unkn.	Indir.fr.fluor.	Igs, C1q, C3, C4, C4d, CD59	Creat., proteinuria, Banff classification
104	2009	Acute antibody-mediated rejection, living donor	1	D: unkn.<br>R: 20	D: 0%<br>R: 100%	Biopsy	aE11	Indir.fr.fluor.	C4d	Not applicable
212	2012	Acute antibody-mediated rejection with TMA	1	R: 13	R: 0%	Biopsy	Unkn.	Unkn.	C4d	Not applicable
211	2013	After cold storage and 45 min after reperfusion, living or deceased donor	33	D: 17-84[c]<br>R: 10-83[c]	D: 58%<br>R: 61%	Biopsy	aE11	Indir.fix.perox.	None	None
		Acute rejection	1	Unkn.	Unkn.	Idem	Idem	Idem	Idem	Not applicable
130	2015	Rejection with TMA	3	R: 32-72	33%	Biopsy	A239	Indir.fix.perox.	C1q, C4d, MBL	None
54	2018	Delayed transplant function	3	R: 47-58	R: 67%	Biopsy	ab55811	Indir.fix.perox.	C3d	Age, sex, HLA mismatch
		Acute rejection	7	R: 28-47	R: 57%	Idem	Idem	Idem	Idem	Idem
		Chronic rejection	2	R: 24-27	R: 100%	Idem	Idem	Idem	Idem	Idem
134	2019	Acute or chronic antibody-mediated rejection, mostly from deceased donor	54	R: 21-69[c]	R: 63%	Biopsy	B7	Indir.fix. perox.	Igs, C1q, C3, C4d	Creat., proteinuria, donor-specific antibodies, histological lesions, transplant survival
213	2019	Protocol or indication biopsy after ABO-incompatible transplantation	30	D: 29-78[c]<br>R: 27-76[c]	D: 63%<br>R: 63%	Biopsy	Unkn.	Unkn.fr. fluor.	C1q, C3c, C4d	Anti-ABO antibodies before transplantation, rejection

**Other kidney diseases studied only incidentally not discussed in the text**									
44	1983	Amyloidosis	2	Unkn.	Unkn.	Biopsy or nephrect.	PolyC9-MA	Indir.fr.fix.fluor.	Igs, C1q, C3, C4, C5, C6, C7, C8, C9	None
96	1986	Scleroderma with TMA	1	Adult	Unkn.	Biopsy	Anti-C5b-9(m)	Indir.fr.fluor.	Igs, C1q, C3b/C3c, C3d, C3g, C5, C8, C9, FH	Not applicable
96	1986	Amyloidosis	2	Adults	Unkn.	Biopsy	Anti-C5b-9(m)	Indir.fr.fluor.	Igs, C1q, C3b/C3c, C3d, C3g, C5, C8, C9, FH	None
96	1986	Myeloma or light chain disease	4	Adults	Unkn.	Biopsy	Anti-C5b-9(m)	Indir.fr.fluor.	Igs, C1q, C3b/C3c, C3d, C3g, C5, C8, C9, FH	None
72	1989	Amyloidosis	1	Adult	Unkn.	Biopsy	Anti-C5b-9(m)	Indir.fr.fluor.	Igs, C3, Vn	Not applicable
75	1993	Oligomeganephronia	1	Unkn.	Unkn.	Biopsy	PolyC9-MA	Indir.fr.fix.fluor.	Igs, C3c, C3d, C5, C9, Vn, Vn receptor	Not applicable
219	1995	Anti-glomerular basement membrane disease	1	70	0%	Biopsy	PolyC9-MA	Indir.fr.fix.fluor.	Igs, C1q, C3, C4, IL6, immune cells, fibr.	Not applicable
220	2000	Primary FSGS	13	14-77[d]	60%[d]	Biopsy	aE11	Indir.fr.perox.	Igs, C3, ICAM1, αSMA, α3β1-integrin, TGFβ1	Creat., treatment effect
221	2005	Renal sarcoidosis	1	57	0%	Biopsy	Unkn.	Perox.	Igs, C1q, C3, C4d, MBL, FBb	Not applicable
222	2012	Mixed type II cryoglobulinemia	1	55	0%	Biopsy	Unkn.	Unkn.	Igs, C3	Not applicable
130	2015	Medication-induced TMA	2	40-47	0%	Biopsy	A239	Indir.fix.perox.	C1q, C4d, MBL	Idem
121	2014	Anti-GBM disease	10	20-66	70%	Biopsy	Unkn.	Indir.fix.perox.	Igs, C1q, C3c, C3d, C4d, FB, P, fibr.	Crescents
223	2016	Scleroderma renal crisis	1	28	0%	Biopsy	Unkn.	Unkn.	C1q, C3, C4d	Not applicable
224	2018	Sickle cell disease nephropathy	3	Unkn.	Unkn.	Biopsy	C9	Unkn.	Igs, C3c	None
225	2018	aHUS, pauci-immune crescentic glomerulo-nephritis, Alport syndr.	1	26	0%	Biopsy	aE11	Unkn.	Igs, C1q, C3, C4d, collagen	Not applicable
226	2020	Acute kidney injury due to rhabdomyolysis	6	5-88[d]	Unkn.	Biopsy	B7	Indir.fix.perox.	C1q, C3c, C3d, C4d, FH, lamp-1, collectin-11, CD31	None

[a] Ages are given as ranges in years.

[b] The method of staining of C5b-9 is specified according to the antibody and immunohistochemical technique. The antibodies are indicated according to their clone names as reported in the original studies or indicated as unknown if a clone name was not given. The antibodies' names correspond with those specified in Table 2. The immunohistochemical techniques are described as direct (dir.) or indirect (indir.), as fixed (fix.), frozen (fr.), or both, and as immunofluorescence (fluor.) or immunoperoxidase (perox.) and indicate the additional use of immunoelectron microscopy (imm.electr.).

[c] An estimated range is given based on a mean with standard deviation or median with interquartile range reported in the original study, calculated as the mean minus and plus two standard deviations or as the median minus two times the lower interquartile range and plus two times the higher interquartile range.

[d] This characteristic was reported in the original study for a larger population, in an unspecified part of which deposition of C5b-9 was studied.

Cn: clusterin; CR1: complement receptor 1; creat.: creatinine; D: donors; DAF: decay-accelerating factor; Fn: fibronectin; FSGS: focal segmental glomerulosclerosis; IFTA: interstitial fibrosis and tubular atrophy; Igs: immunoglobulins; immunofl.: immunofluorescence; MCP: membrane cofactor protein; micr.: microscopy; MPGN: membranoproliferative glomerulonephritis; nephrect.: nephrectomy; P: properdin; R: recipients; ref: reference; syndr.: syndrome; TMA: thrombotic microangiopathy; unkn.: unknown; Vn: vitronectin. Other abbreviations are explained in the article.

SUPPLEMENTARY TABLE 2 | Deposits of C5b-9 in healthy and diseased human kidneys

Ref.	Year	*N*	Ages[a]	Males	Tissue	Antibody	Type, class	Staining of C5b-9[b]				
								Glom.	Mes.	Cap.	Tub.	Vas.
**Healthy kidneys**												
95	1981	3	Unkn.	Unkn.	Unkn.	Kolb 1975					○	
44	1983	Unkn.	Fetus	Unkn.	Unkn.	PolyC9-MA			○	○	○	○
		Unkn.	Adults	Unkn.	Unkn.	PolyC9-MA			●	○	○	●
46	1985	Unkn.	Unkn.	Unkn.	Autopsy	aE11		○			●	
96	1986	6	Adults	Unkn.	Biopsy	αC5b-9(m)		●			●	●
		2	Adults	Unkn.	Nephrect.	αC5b-9(m)		●			●	●
67	1987	6	Unkn.	Unkn.	Biopsy	PolyC9-MA			● ±	○	● +	● +/++
126	1987	1	0	Unkn.	Autopsy or unkn.	PolyC9-MA			●	○	○	●
		2	55-65	Unkn.	Autopsy or unkn.	PolyC9-MA			●	●	●	●
127	1987	4	Unkn.	Unkn.	Unkn.	PolyC9-MA			●	○	●	●
76	1987	3	Unkn.	Unkn.	Autopsy	C5 and C9		○				
137	1987	2	Unkn.	Unkn.	Nephrect.	PolyC9-MA		○ 0%				● 100% +++
52	1987	4	5-37	75%	Biopsy	αMAC-neo			○ 0%	○ 0%		
97	1988	4	43-66	Unkn.	Unkn.	PolyC9-MA			●	○	●	●
70	1989	28	Unkn.	Unkn.	Biopsy	C6 and C9			○	○	● +	● +
72	1989	3	Adults	Unkn.	Biopsy	αC5b-9(m)			●	○	●	●
77	1989	8	Unkn.	Unkn.	Autopsy	Unkn.			●			
75	1993	Unkn.	Unkn.	Unkn.	Nephrect.	PolyC9-MA			●	○	●	●
135	1993	8	Unkn.	Unkn.	Unkn.	Unkn.					●	
110	1995	4	Unkn.	Unkn.	Nephrect.	aE11		○			○	
94	1995	8	Unkn.	Unkn.	Unkn.	Unkn.					●	●
111	1996	7	Unkn.	Unkn.	Nephrect.	WU-7,2			● 43% +	○ 0%	○	●
138	1996	10	Unkn.	Unkn.	Unkn.	PolyC9-MA						●
139	1997	6	47-75[c]	Unkn.	Nephrect.	Unkn.					○	●
128	2001	5	Unkn.	Unkn.	Nephrect.	aE11		●				
112	2002	5	Unkn.	Unkn.	Biopsy	aE11			○ 0%	○ 0%	○ 0%	○ 0%
129	2003	15	21-72[d]	Unkn.	Biopsy	aE11			●	○	●	
84	2004	12	50-88	50%	Autopsy	Unkn.						●
83	2004	10	Unkn.	Unkn.	Biopsy	Unkn.		○ 70%			● 100%	○
113	2004	Unkn.	Unkn.	Unkn.	Biopsy	Unkn.		○				○

114	2006	7	Children	Unkn.	Unkn.	1B4				○	0%		○	0%							
115	2008	Unkn.	Unkn.	Unkn.	Nephrect.	aE11				○			○			○			○		
116,117	2009, 2010	2	Unkn.	Unkn.	Nephrect. or unkn.	Unkn.	○	0%													
118	2011	1	Unkn.	Unkn.	Unkn.	Unkn.										●					
103	2012	6	Unkn.	Unkn.	Biopsy	aE11				●		+/++	●		+/++	●		++	●		+++
119	2013	5	Unkn.	Unkn.	Autopsy	WU-13,15	○									○			○		
120	2013	1	Adult	100%	Nephrect.	Unkn.	○									○			○		
121	2014	1	Unkn.	Unkn.	Nephrect.	Unkn.	○														
131	2015	25	Unkn.	0%	Autopsy	Unkn.	●	4%													
130	2015	9	Unkn.	Unkn.	Unkn.	A239													●	78%	
122	2016	1	Adult	100%	Nephrect.	Unkn.	○												○		
123	2017	14	7-62[c]	57%	Biopsy	Unkn.				○			○								
124	2017	15	8-56[c]	47%	Biopsy	aE11	○	0%													
133	2018	3	Unkn.	Unkn.	Unkn.	Unkn.	●	33%	±												
136	2018	74	29-95[c]	69%	Autopsy	Unkn.										●	34%				
27	2018	41	30-97[c]	61%	Autopsy	Unkn.				●	31%		●	29%					●	98%	
		10	Unkn.	Unkn.	Biopsy	Unkn.	○	0%													
132	2018	12	23-83	Unkn.	Autopsy or biopsy	aE11				●			○								
28	2018	11	37-73[c]	45%	Nephrect.	aE11				●						●			●		
31	2019	Unkn.	Unkn.	Unkn.	Nephrect.	aE11										○					
18	2019	6	Unkn.	Unkn.	Unkn.	aE11				●	17%	±	○	0%		●	17%	+/++	●		
134	2019	1	Unkn.	Unkn.	Biopsy	B7				○			○						●		
102	2019	1	Unkn.	Unkn.	Biopsy	aE11	●														
		1	Unkn.	Unkn.	Biopsy	aE11	○														
125	2020	Unkn.	Unkn.	Unkn.	Unkn.	aE11	○									○			○		

**Minimal change nephropathy**																					
96	1986	5	Adults	Unkn.	Unkn.	αC5b-9(m)				●	100%	+	○	0%		●			●		
141	1986	3	2-14[d]	Unkn.	Biopsy	αC5b-9(m)				○			○			○			○		
67	1987	7	Unkn.	Unkn.	Biopsy	PolyC9-MA				●		±	○			●		+	●		+/++
98	1987	3	2-14[d]	Unkn.	Biopsy	αC5b-9(m)				○			○			○			○		
72	1989	3	Adults	Unkn.	Unkn.	αC5b-9(m)				●			○			●			●		
70	1989	3	Unkn.	Unkn.	Biopsy	C6 and C9				○			○			●		++	●		+/++
57	1989	6	Unkn.	Unkn.	Biopsy	αMAC				●			○			●			●		
61	1990	3	Unkn.	Unkn.	Biopsy	Xia 1988	○	0%													
81	1991	9	Unkn.	Unkn.	Biopsy	PolyC9-MA				●			○								
75	1993	10	Unkn.	Unkn.	Biopsy	PolyC9-MA				●			○			●			●		
79	1994	2	18-23	50%	Biopsy	A239				●		+	○			●		+++			
139	1997	1	15	100%	Biopsy	Unkn.	○														
142	1999	5	Unkn.	Unkn.	Unkn.	Unkn.										●	80%				
128	2001	5	15-34	60%	Biopsy	aE11	●	100%	+												
143	2002	4	Unkn.	Unkn.	Biopsy	Unkn.	○	0%								●			●		
113	2004	1	Unkn.	Unkn.	Biopsy	Unkn.	○												○		
85	2005	10	Unkn.	Unkn.	Unkn.	Unkn.	○														
86	2007	10	Unkn.	Unkn.	Unkn.	Unkn.	○														
87	2010	10	Unkn.	Unkn.	Unkn.	Unkn.	○														
116, 117	2009, 2010	8	Unkn.	Unkn.	Biopsy	Unkn.	●	25%	++												
121	2014	5	Unkn.	Unkn.	Biopsy	Unkn.	○														
18	2019	4	Unkn.	Unkn.	Unkn.	aE11				●	100%	+	○	0%		●	100%	++	●		
**Glomerular basement membrane diseases**																					
96	1986	1	Adult	Unkn.	Unkn.	αC5b-9(m)				●			○			●			●		
139	1997	1	42	0%	Biopsy	Unkn.	●		+												
143	2002	12	Unkn.	Unkn.	Biopsy	Unkn.	○	0%								●			●		
85	2005	10	Unkn.	Unkn.	Biopsy	Unkn.	○														
86	2007	10	Unkn.	Unkn.	Unkn.	Unkn.	○														
87	2010	10	Unkn.	Unkn.	Biopsy	Unkn.	○	0%													
130	2015	5	Unkn.	Unkn.	Biopsy	A239	○	0%											○	0%	
18	2019	4	Unkn.	Unkn.	Unkn.	aE11				●	100%	±	●	25%	−	●	75%	±	●		

**Hypertensive nephropathy**																						
95	1981	2	Unkn.	Unkn.	Unkn.	Kolb 1975											○					
44	1983	3	Unkn.	Unkn.	Biopsy or nephrect.	PolyC9-MA					●			○			●			●		
67	1987	6	Unkn.	Unkn.	Biopsy	PolyC9-MA					●		+	○			●		+/++	●		+++
143	2002	2	Unkn.	Unkn.	Biopsy	Unkn.		●	50%								●			●		
84	2004	7	54-84	71%	Autopsy	Unkn.														●		
		9	65-88	33%	Autopsy	Unkn.														●[e]		
131	2015	11	26-40[c]	0%	Autopsy	Unkn.		●[e]	9%[e]													
		14	Unkn.	0%	Autopsy	Unkn.		●[e]	7%[e]													
**Diabetic nephropathy**																						
44	1983	7	Unkn.	Unkn.	Biopsy or nephrect.	PolyC9-MA					●			○			●			●		
96	1986	2	Adults	Unkn.	Unkn.	αC5b-9(m)					●	100%	++	○	0%		●			●		
67	1987	9	Unkn.	Unkn.	Biopsy	PolyC9-MA					●		+	○			●		++	●		++
126	1987	12	Unkn.	Unkn.	Biopsy or nephrect.	PolyC9-MA					●			●	25%		●			●		
70	1989	3	Unkn.	Unkn.	Biopsy	C6 and C9					○			○			●			●		
72	1989	3	Adults	Unkn.	Unkn.	αC5b-9(m)		●									●			●		
139	1997	2	41-59	50%	Biopsy	Unkn.		●	100%	+/++												
142	1999	3	Unkn.	Unkn.	Unkn.	Unkn.											●	67%				
143	2002	2	Unkn.	Unkn.	Biopsy	Unkn.		●	100%								●			●		
113	2004	13	45-77	42%	Biopsy	Unkn.		●												●		
		1	Unkn.	Unkn.	Biopsy	Unkn.		●												●		
84	2004	27	40-86	81%	Autopsy	Unkn.	2													●		
28	2018	62	27-79[c]	66%	Biopsy	aE11	2	●									●			●		
27	2018	101	43-95[c]	53%	Autopsy	Unkn.	1, 2				●	73%		●	71%					●	100%	
		12	Unkn.	Unkn.	Biopsy	Unkn.	1, 2	●														
		58	44-94[c]	59%	Autopsy	Unkn.	1, 2				●[e]	62%[e]		●[e]	59%[e]					●[e]	100%[e]	

**Membranous nephropathy**																						
44	1983	3	Unkn.	Unkn.	Biopsy	PolyC9-MA					○			●			●					
96	1986	6	Adults	Unkn.	Unkn.	αC5b-9(m)	Idio.				○	0%		●	100%	++	●			●		
		9	Adults	Unkn.	Unkn.	αC5b-9(m)	Sec.				●[f]	78%[f]	+[f]	●[f]	22%[f]	+/++[f]	●[f]			●[f]		
141	1986	2	2-14[d]	Unkn.	Biopsy	αC5b-9(m)								●	100%	++						
98	1987	2	2-14[d]	Unkn.	Biopsy	αC5b-9(m)		●	100%													
67	1987	11	Unkn.	Unkn.	Biopsy	PolyC9-MA								●		++	●		+/++	●		+++
72	1989	7	Adults	Unkn.	Unkn.	αC5b-9(m)	Idio.	●	100%								●			●		
70	1989	9	Unkn.	Unkn.	Biopsy	C6 and C9					○			●		++	●		+/++	●		+++
57	1989	22	Unkn.	Unkn.	Biopsy	αMAC		●		++							●			●		
77	1989	12	Unkn.	Unkn.	Biopsy	Unkn.	Idio.				○			●	50%	+						
		8	Unkn.	Unkn.	Biopsy	Unkn.	Sec.				○[f]			●[f]	13%[f]	+[f]						
152	1989	2	5-15	100%	Biopsy	PolyC9-MA	Idio.				●			●	100%							
		6	3-13	33%	Biopsy	PolyC9-MA	Sec.				●[f]			●[f]	83%[f]							
81	1991	18	Unkn.	Unkn.	Biopsy	PolyC9-MA	Idio.	●														
75	1993	2	Unkn.	Unkn.	Biopsy	PolyC9-MA	Idio.							●			●					
79	1994	6	47-65	33%	Biopsy	A239					●		+	●		+++	●		+			
82	1995	5	Unkn.	Unkn.	Biopsy	C9	Idio.	●														
139	1997	7	23-70	29%	Biopsy	Unkn.		●	100%	+/++												
142	1999	3	Unkn.	Unkn.	Unkn.	Unkn.											●	100%				
112	2002	35	23-71	66%	Biopsy	aE11	Idio.				●	23%		●	100%		●	100%		●	80%	
143	2002	10	Unkn.	Unkn.	Biopsy	Unkn.		●	100%													
148	2004	20	44-57[c]	70%	Biopsy	aE11	Idio.	●	100%													
149	2004	1	Unkn.	100%	Biopsy	Unkn.					●			●								
80	2006	60	31-86[c]	63%	Biopsy	Unkn.	Idio.	●														
156	2010	24	28-75	67%	Biopsy	aE11	Idio.							●								
87	2010	16	2-23	Unkn.	Biopsy	Unkn.	Idio.							●	100%	++						
150	2011	8	39-77	38%	Biopsy	aE11	Prim.							●								
151	2011	Unkn.	Unkn.	Unkn.	Biopsy	Unkn.											●					
147	2012	1	56	100%	Biopsy	Unkn.	Prim.							●								
157	2014	1	6	100%	Biopsy	Unkn.	Sec.							●[f]								
153	2014	Unkn.	Unkn.	Unkn.	Unkn.	aE11		●														
154	2015	1	0	0%	Biopsy	Unkn.	Prim.				○			●								
155	2016	1	25	100%	Biopsy	Unkn.	Prim.							●								
123	2017	17	1-82[c]	65%	Biopsy	Unkn.	Idio.				○			●								
18	2019	5	Unkn.	Unkn.	Unkn.	aE11	Idio.				○	0%		●	100%	+++	●	50%	±	●		

**IgA nephropathy and IgA vasculitis with nephritis**																						
44	1983	3	Unkn.	Unkn.	Biopsy	PolyC9-MA	Neph.				●			○			●					
96	1986	7	Adults	Unkn.	Unkn.	αC5b-9(m)	Neph.				●	100%	+/++	●	14%	+	●			●		
		2	Adults	Unkn.	Unkn.	αC5b-9(m)	Vasc.				●[f]	100%[f]	+[f]	○[f]	0%		●[f]			●[f]		
137	1987	12	Unkn.	Unkn.	Biopsy	PolyC9-MA	Neph.	●	100%	++										●	100%	++
67	1987	11	Unkn.	Unkn.	Biopsy	PolyC9-MA	Neph.				●		+++	○			●		+/++	●		+++
52	1987	23	6-56	78%	Biopsy	αMAC-neo	Neph.				●	100%	++	●	56%	++	●	76%		●	76%	
		2	9-10	50%	Biopsy	αMAC-neo	Vasc.				●[f]	100%[f]	+/++[f]	●[f]	100%[f]	+/++[f]						
76	1987	15	Unkn.	Unkn.	Biopsy	C5 and C9	Neph.	●	73%	+/++												
97	1988	30	4-17	70%	Biopsy	PolyC9-MA	Neph.		73%		●			●			●	93%				
70	1989	16	Unkn.	Unkn.	Biopsy	C6 and C9	Neph.				●		++	○			●		++	●		+/++
72	1989	10	Adults	Unkn.	Unkn.	αC5b-9(m)	Neph.	●									●			●		
57	1989	75	Unkn.	Unkn.	Biopsy	αMAC	Neph.	●		+/++							●			●		
61	1990	4	Unkn.	Unkn.	Biopsy	Xia 1988	Vasc.	●[f]	100%[f]	+/++[f]												
81	1991	31	Unkn.	Unkn.	Biopsy	PolyC9-MA	Neph.	●														
162	1991	14	Unkn.	Unkn.	Unkn.	Unkn.	Neph.				●	100%								●	100%	
93	1991	1	23	100%	Biopsy	PolyC9-MA	Neph.	●		+												
75	1993	22	Unkn.	Unkn.	Biopsy	PolyC9-MA	Neph.				●						●					
		7	Unkn.	Unkn.	Biopsy	PolyC9-MA	Vasc.				●[f]						●[f]					
110	1995	20	19-45	60%	Biopsy	aE11	Neph.	●	95%								●	90%		●	75%	
163	1995	54	Children	61%	Biopsy	Unkn.	Neph.			++	●	100%		●	37%		●	69%				
58	1995	2	Unkn.	Unkn.	Biopsy	aE11, B7, αC5b-9(m)	Neph.	●														
139	1997	11	15-45	91%	Biopsy	Unkn.	Neph.	●	100%	+/++												
		2	6-9	50%	Biopsy	Unkn.	Vasc.	●[f]	100%[f]	+/++[f]												
161	1997	120	7-53	42%	Biopsy	Unkn.	Neph.	●														
160	1998	45	15-48	47%	Biopsy	aE11	Neph.	●	100%													
142	1999	8	Unkn.	Unkn.	Unkn.	Unkn.	Neph.										●	38%				
99	2000	10	9-23	40%	Biopsy	aE11	Vasc.	●[f]	100%[f]													
128	2001	14	20-51	43%	Biopsy	aE11	Neph.	●	100%	++												
143	2002	20	Unkn.	Unkn.	Biopsy	Unkn.	Neph.	●	90%													
85	2005	31	4-18	Unkn.	Biopsy	Unkn.	Vasc.				●[f]	87%[f]	+[f]									
115	2008	30	17-67[d]	75%[d]	Biopsy	aE11	Neph.			+/++	●	100%		●	100%		●	100%		●	81%	
164	2010	35	27-39[c]	60%	Biopsy	Unkn.	Neph.				●			●								
159	2014	1	16	100%	Biopsy	Unkn.	Neph.							●								
130	2015	1	32	100%	Biopsy	A239	Neph.	○												○		
165	2017	96	4-66[c]	65%	Biopsy	aE11	Neph.				●	100%										

124	2017	25	0-63[c]	80%	Biopsy	aE11	Neph.	●	100%	+												
158	2018	36	18-84[d]	66%[d]	Biopsy	Unkn.	Neph.	●	67%	+/++												
167	2019	116	0-84	69%	Biopsy	A239	Both	●	15%											●	50%	
19	2020	67	3-17	61%	Biopsy	Unkn.	Both				●	81%					●	82%		●		
166	2020	132	17-82	54%	Biopsy	Unkn.	Neph.				●		+/++									
**Lupus nephritis**																						
95	1981	2	Unkn.	Unkn.	Autopsy or biopsy	Kolb 1975	II				●	100%		●	100%		●	100%	+	●		
		7	Unkn.	Unkn.	Autopsy or biopsy	Kolb 1975	III				●	86%	+	●	86%	+	●	100%	+/++	●		
		10	Unkn.	Unkn.	Autopsy or biopsy	Kolb 1975	IV				●	100%	++	●	100%	++	●	100%	++	●		
		3	Unkn.	Unkn.	Autopsy or biopsy	Kolb 1975	V				○	0%		●	67%	+/++	●	100%	++	●		
44	1983	3	Unkn.	Unkn.	Biopsy	PolyC9-MA					●			●			●					
46	1985	1	18	0%	Unkn.	aE11		●														
96	1986	1	Adult	Unkn.	Biopsy	αC5b-9(m)	II				●		+	○			●			●		
		8	Adults	Unkn.	Biopsy	αC5b-9(m)	IV				●	100%	++	●	88%	++	●			●		
67	1987	8	Unkn.	Unkn.	Biopsy	PolyC9-MA	III, IV				●		++	●		++	●		+/++	●		+/++
72	1989	7	Adults	Unkn.	Unkn.	αC5b-9(m)	II-IV	●	100%								●			●		
70	1989	10	Unkn.	Unkn.	Biopsy	C6 and C9					●		+	●		++	●		+/++	●		+
77	1989	8	Unkn.	Unkn.	Biopsy	Unkn.	V				○	0%		●	75%	+/++						
152	1989	1	18	0%	Biopsy	PolyC9-MA	V							●								
57	1989	20	Unkn.	Unkn.	Biopsy	αMAC		●	79%								●			●		
81	1991	9	Unkn.	Unkn.	Biopsy	PolyC9-MA	IV	●														
75	1993	4	Unkn.	Unkn.	Biopsy	PolyC9-MA	IV				●			●			●					
58	1995	2	Unkn.	Unkn.	Biopsy	aE11, B7, αC5b-9(m)	IV	●														
111	1996	5	Unkn.	Unkn.	Biopsy	WU-7,2	II			+/++	●	100%		○	0%							
		3	Unkn.	Unkn.	Biopsy	WU-7,2	III			+++	●	100%		○	0%							
		7	Unkn.	Unkn.	Biopsy	WU-7,2	IV			+++	●	100%		●	100%							
139	1997	2	19-30	50%	Biopsy	Unkn.	III, V	●	100%	+/++												
142	1999	5	Unkn.	Unkn.	Unkn.	Unkn.	IV										●	100%				
143	2002	7	Unkn.	Unkn.	Biopsy	Unkn.		●	86%													
172	2008	1	27	0%	Biopsy	aE11	V							●								

170	2008	1	30	0%	Biopsy	Unkn.	II				●		+									
151	2011	Unkn.	Unkn.	Unkn.	Biopsy	Unkn.		●														
175	2012	1	27	0%	Biopsy	Unkn.	TMA													●		
119	2013	11	23-41	27%	Biopsy	WU-13,15	II-V										●			●		
		2	23-41[d]	27%[d]	Biopsy	WU-13,15	II	●	100%	+++												
		3	23-41[d]	27%[d]	Biopsy	WU-13,15	III	●	100%	+/++												
		3	23-41[d]	27%[d]	Biopsy	WU-13,15	IV	●	100%	+++												
		3	23-41[d]	27%[d]	Biopsy	WU-13,15	V	●	100%	+/++												
130	2015	8	17-49	0%	Autopsy or biopsy	A239	TMA	●	60%											●	60%	
173	2017	222	10-56[c]	16%	Biopsy	Unkn.	II-VI				●			●								
174	2018	38	Unkn.	Unkn.	Biopsy	Unkn.	II-V				●			●								
133	2018	5	5-18[d]	13%	Biopsy	Unkn.		●	100%	+++												
171	2018	30	8-59[c]	20%	Biopsy	X197	II-V										●			●		
		2	8-59[cd]	20%[d]	Biopsy	X197	II	○	0%													
		5	8-59[cd]	20%[d]	Biopsy	X197	III	●	20%													
		8	8-59[cd]	20%[d]	Biopsy	X197	IV	●	50%													
		5	8-59[cd]	20%[d]	Biopsy	X197	V	●	60%													
		8	8-59[cd]	20%[d]	Biopsy	X197	III+V	●	50%													
		2	8-59[cd]	20%[d]	Biopsy	X197	IV+V	●	50%													
18	2019	20	20-64	20%	Biopsy	aE11	III				●	85%	+	●	65%	+	●	70%	+	●		
		22	21-61	13%	Biopsy	aE11	IV				●	87%	+/++	●	59%	+/++	●	82%	+	●		
		13	18-71	23%	Biopsy	aE11	V				●	46%	−	●	100%	+++	●	69%	−	●		
**Membranoproliferative glomerulonephritis, C3 glomerulopathy, and postinfectious glomerulonephritis**																						
44	1983	3	Unkn.	Unkn.	Biopsy	PolyC9-MA	I										●					
		2	Unkn.	Unkn.	Nephrect.	PolyC9-MA	II				●			●			●					
183	1984	11	5-14	45%	Biopsy	PolyC9-MA	Pinf.				●[f]	86%[f]		●[f]	86%[f]							
96	1986	9	Adults	Unkn.	Biopsy	αC5b-9(m)	I				●	89%	++	●	100%	++	●			●		
141	1986	2	2-14[d]	Unkn.	Biopsy	αC5b-9(m)					○	0%		●	50%	+	●	50%	+			
98	1987	2	2-14[d]	Unkn.	Biopsy	αC5b-9(m)		●	50%													
67	1987	3	Unkn.	Unkn.	Biopsy	PolyC9-MA	I				●		+++	●		++	●		+/++	●		++
		4	Unkn.	Unkn.	Biopsy	PolyC9-MA	Pinf.				●[f]		++[f]	●[f]		++[f]	●[f]		++[f]	●[f]		++[f]
126	1987	3	Unkn.	Unkn.	Biopsy	PolyC9-MA	II				●			●			●					
72	1989	5	Adults	Unkn.	Unkn.	αC5b-9(m)	I	●	100%								●			●		
57	1989	11	Unkn.	Unkn.	Biopsy	αMAC	I	●									●			●		
		1	Unkn.	Unkn.	Biopsy	αMAC	II	●									●			●		

75	1993	1	Unkn.	Unkn.	Biopsy	PolyC9-MA	I				●			●			●					
		2	Unkn.	Unkn.	Biopsy	PolyC9-MA	II				●			●			●					
		1	Unkn.	Unkn.	Biopsy	PolyC9-MA	III				●			●			●					
184	1994	5	3-17[d]	80%[d]	Biopsy	Unkn.	Pinf.				●[f]	80%[f]		●[f]	100%[f]							
139	1997	4	20-49	100%	Biopsy	Unkn.	I	●	100%	+/++												
143	2002	1	Unkn.	Unkn.	Biopsy	Unkn.	I	●														
		2	Unkn.	Unkn.	Biopsy	Unkn.	Pinf.	●	100%[f]													
114	2006	18	Children	56%[d]	Biopsy	1B4	I		94%	+/++	●			●								
86	2007	18	4-23	Unkn.	Biopsy	Unkn.	Pinf.		100%[f]	+++[f]	●[f]			●[f]								
179	2009	2	7-12	0%	Biopsy	Unkn.	C3GN				●	100%		●	100%							
105	2012	1	17	Unkn.	Biopsy	Unkn.	DDD	●														
103	2012	2	20-42[d]	100%	Biopsy	aE11	C3GN				●	100%	+++	●	100%	+++	●	100%	+++	●	100%	+++
		2	20-42[d]	100%	Biopsy	aE11	DDD				●	100%	+++	●	100%	+++	●	100%	+++	●	100%	+++
181	2014	3	8-28	33%	Biopsy	Unkn.	DDD	●	67%													
106	2015	3	27-63	33%	Biopsy	Unkn.	C3G	●	100%	+++												
107	2015	1	5	100%	Biopsy	Unkn.	C3GN				●			●								
182	2016	1	28	100%	Nephrect.	Unkn.	C3G				●											
101	2018	3	7-12	0%	Biopsy	Unkn.	DDD	●		++												
102	2019	24	9-74[d]	52%[d]	Biopsy	aE11	C3G	●	100%	+/++												
		8	9-74[d]	52%[d]	Biopsy	aE11	C3G	●	100%	++												
108	2020	2	14-15	50%	Biopsy	Unkn.	IC							●	100%							
**Thrombotic microangiopathy**																						
103	2012	1	Unkn.	Unkn.	Biopsy	aE11	aHUS				●		+++	●		+++	●		+++	●		
190	2013	1	26	0%	Biopsy	WU-13,15	STEC	●														
120	2013	2	2-4	0%	Biopsy or unkn.	Unkn.	TTP	●									●			●		
153	2014	10	22-44[d]	18%[d]	Biopsy	aE11	STEC	○														
130	2015	11	22-77	18%	Autopsy or biopsy	A239	aHUS	●												●		
		1	14	100%	Biopsy	A239	STEC	○												○		
		6	18-54	50%	Autopsy or biopsy	A239		●												●		
		3	6-37	67%	Biopsy or nephrect.	A239	aHUS	●												●		
188	2015	1	16	100%	Biopsy	aE11	aHUS				●			●						●		

122	2016	1	2	100%	Biopsy	Unkn.	STEC							●						●		
192	2017	7	29-65	38%	Biopsy	Unkn.	Hypt.							●	100%	++				●	100%	+++
		2	38	50%	Biopsy	Unkn.	Hypt.							●	100%	++				●	100%	+
132	2018	5	9-40	60%	Biopsy	aE11	TTP				○	0%		●	40%					●	60%	
		8	22-66	Unkn.	Autopsy<br>or biopsy	aE11	TTP				○	0%		●	20%					●	40%	
144	2018	10	23-72	57%	Biopsy	Unkn.	Hypt.							●						●		
100	2019	6	22-65	50%	Biopsy	aE11					●	100%	++	●	33%	+/++	●			●	100%	+++
		7	32-72	29%	Biopsy	aE11					●	100%	+/++	●	14%	++	●			●	100%	+++
193	2020	15	17-61[cd]	58%[d]	Biopsy	Unkn.	Hypt.							●	47%							
**ANCA-associated vasculitis**																						
139	1997	1	44	0%	Biopsy	Unkn.		●														
143	2002	6	Unkn.	Unkn.	Biopsy	Unkn.		●	17%													
195	2006	34	25-80	50%	Biopsy	aE11		●		++							●			●		+
196	2008	34	>15	50%	Biopsy	aE11		●									●					
116	2009	7	51-75	71%	Biopsy	Unkn.			100%	++	●			●						●		
117	2010	12	21-70	50%	Biopsy	Unkn.				+++	●			●	100%					●	100%	
22	2013	29	28-89[c]	41%	Biopsy	Unkn.					●			●						●		
53	2015	25	35-95[cd]	63%[d]	Biopsy	Ab55811		●	60%	+/++												
130	2015	1	35	100%	Biopsy	A239		●									●			●		
194	2017	49	32-90[cd]	70%[d]	Biopsy	Unkn.		●	78%	+												
**Interstitial nephritis**																						
96	1986	1	Adult	Unkn.	Biopsy	αC5b-9(m)					●	100%	+	○	0%		●			●		
70	1989	2	Unkn.	Unkn.	Biopsy	C6 and C9											●		+++	●		+++
139	1997	46	3-70	61%	Biopsy	Unkn.											●			●		
143	2002	3	Unkn.	Unkn.	Biopsy	Unkn.		●	33%								●			●		
118	2011	16	Unkn.	Unkn.	Biopsy	Unkn.											●					
199	2011	1	59	100%	Biopsy	Unkn.											●		+			
31	2019	54	18-76[c]	43%	Biopsy	aE11											●					

**Acute tubular necrosis**																					
203	1993	1	21	100%	Biopsy	PolyC9-MA	○									●			●		
135	1993	31	Unkn.	Unkn.	Biopsy	Unkn.										●					
94	1995	45	Unkn.	Unkn.	Unkn.	Unkn.										●			●		
142	1999	21	Unkn.	Unkn.	Unkn.	Unkn.										●					
200	2005	4	20-80[d]	Unkn.	Biopsy	C8										●					
136	2018	21	35-93[cd]	62%[d]	Autopsy	Unkn.										●					
31	2019	5	25-65	60%	Biopsy	aE11										●					
125	2020	6	51-86	67%	Autopsy	aE11	●	33%								●	100%	+/++	●	33%	
**Reflux nephropathy**																					
44	1983	3	Unkn.	Unkn.	Biopsy or nephrect.	PolyC9-MA				●			○			●			●		
127	1987	8	8-51	13%	Biopsy or nephrect.	PolyC9-MA	●									●			●		
75	1993	1	Unkn.	Unkn.	Biopsy	PolyC9-MA	●														
**Kidney tumors**																					
210	1996	22	39-82	59%	Biopsy	Unkn.	●[h]	82%[h]	+/++[h]												
138	1996	10	Unkn.	Unkn.	Unkn.	PolyC9-MA	●[h]	10%[h]	+[h]										●	60%	++
209	2000	31	Unkn.	Unkn.	Nephrect.	aE11	●[h]	16%[h]	+[h]												
207	2020	20	37-81[c]	57%	Nephrect.	aE11	○[h]														
**Kidney transplantation[g]**																					
96	1986	2	Adults	Unkn.	Biopsy	αC5b-9(m)				●	100%	+	○	0%		●			●		
214	1986	13	10-43	38%	Biopsy	αC5b-9(m)				●[f]	40%[f]		●[f]	60%[f]							
67	1987	4	Unkn.	Unkn.	Biopsy	PolyC9-MA				●		+/++	○			●		+/++	●		+/++
70	1989	14	Unkn.	Unkn.	Biopsy	C6 and C9				●			●			●			●		
143	2002	4	Unkn.	Unkn.	Biopsy	Unkn.	●	25%								●			●		
129	2003	37	20-77	57%	Biopsy	aE11				●[e]			○[e]			●[e]					
83	2004	10	21-44[c]	40%	Biopsy	Unkn.	●	50%								●	50%				
104	2009	1	20	100%	Biopsy	aE11										●			●		
212	2012	1	13	0%	Biopsy	Unkn.				●			○						●		
211	2013	33	10-83[c]	61%	Biopsy	aE11										○[e]			○[e]		
		1	Unkn.	Unkn.	Biopsy	aE11										●			●		

54	2018	3	47-58	67%	Biopsy	ab55811	●[f]	100%[f]								●[f]					
		7	28-47	57%	Biopsy	ab55811	●	86%								●					
		2	24-27	100%	Biopsy	ab55811	●	100%								●					
134	2019	54	21-69[c]	63%	Biopsy	B7				○	0%		●	24%	+	●			●	2%	++
213	2019	15	27-76[c]	63%[d]	Indication biopsy	Unkn.	●	55%	±							●	75%	+	●	25%	−
		15	27-76[c]	63%[d]	Protocol biopsy	Unkn.	●[e]	22%[e]	−[e]							●[e]	55%[e]	±[e]	●[e]	15%[e]	−[e]

[a] Ages are given as ranges in years.

[b] The staining of C5b-9 is described as absent (○) or present (●), as the proportion of patients exhibiting staining (%), and as the mean or median staining intensity on a scale from – to +++ in the glomerulus as a whole (glom.), the mesangium (mes.), the glomerular capillary wall (cap.), the tubules (tub.), and the extraglomerular vascular wall (vas.). The proportion of patients exhibiting staining is not given for studies including only one patient. The staining intensity is not given for studies including only patients exhibiting no staining. Nothing is indicated if the data were not reported.

[c] An estimated range is given based on a mean with standard deviation or median with interquartile range reported in the original study, calculated as the mean minus and plus two standard deviations or as the median minus two times the lower interquartile range and plus two times the higher interquartile range.

[d] This characteristic was reported in the original study for a larger population, in an unspecified part of which deposition of C5b-9 was studied.

[e] This finding concerns cases with the disease of interest, like hypertension or diabetes, but without related kidney disease, as detailed in the case descriptions in Supplementary Table 1. These cases were omitted from the calculations underlying Figure 1.

[f] This finding concerns cases with secondary membranous nephropathy, IgA vasculitis with nephritis, postinfectious glomerulonephritis, or *de novo* membranous nephropathy after kidney transplantation and was omitted from the calculations underlying Figure 1, as indicated in the figure's legend.

[g] Age ranges and proportions of males are given for the transplant recipients.

[h] This finding concerns staining localized in kidney tumor tissue, which is not classifiable as localized in the glomerulus, tubules, or vascular wall.

α: anti; C3G: C3 glomerulopathy; C3GN: C3 glomerulonephritis; DDD: dense deposit disease; hypt.: hypertension; IC: immune complex-mediated; idio.: idiopathic; neph.: nephropathy; nephrect.: nephrectomy; pinf.: postinfectious; prim.: primary; ref: reference; sec.: secondary; STEC: Shiga toxin-producing enterohemorrhagic *Escherichia coli*; unkn.: unknown; vasc.: vasculitis. Other abbreviations are explained in the article. Antibodies' names correspond with those specified in Table 2.

SUPPLEMENTARY TABLE 3 | Deposits of C5b-9 in healthy and diseased human kidneys as detected with different antibody

Ref.	Year	*N*	Ages[a]	Males	Tissue	Kidney disease	Staining of C5b-9[b]: Glom.	Mes.	Cap.	Tub.	Vas.
**Ab55811**											
53	2015	25	35-95[cd]	63%[d]	Biopsy	ANCA-vasculitis	● 60% +/++				
54	2018	3	47-58	67%	Biopsy	Delayed graft function	● 100%			●	
		7	28-47	57%	Biopsy	Acute rejection	● 86%			●	
		2	24-27	100%	Biopsy	Chronic rejection	● 100%			●	
**aE11 or M0777**											
46	1985	Unkn.	Unkn.	Unkn.	Autopsy	Healthy	○			●	
110	1995	4	Unkn.	Unkn.	Nephrect.	Healthy	○			○	
128	2001	5	Unkn.	Unkn.	Nephrect.	Healthy	●				
112	2002	5	Unkn.	Unkn.	Biopsy	Healthy		○ 0%	○ 0%	○ 0%	○ 0%
129	2003	15	21-72[d]	Unkn.	Biopsy	Healthy		●	○	●	
115	2008	Unkn.	Unkn.	Unkn.	Nephrect.	Healthy		○	○	○	○
103	2012	6	Unkn.	Unkn.	Biopsy	Healthy		● +/++	● +/++	● ++	● +++
124	2017	15	8-56[c]	47%	Biopsy	Healthy	○ 0%				
132	2018	12	23-83	Unkn.	Autopsy or biopsy	Healthy		●	○		
28	2018	11	37-73[c]	45%	Nephrect.	Healthy		●		●	●
31	2019	Unkn.	Unkn.	Unkn.	Nephrect.	Healthy				○	
18	2019	6	Unkn.	Unkn.	Unkn.	Healthy		● 17% ±	○ 0%	● 17% +/++	●
102	2019	1	Unkn.	Unkn.	Biopsy	Healthy	●				
		1	Unkn.	Unkn.	Biopsy	Healthy	○				
125	2020	Unkn.	Unkn.	Unkn.	Unkn.	Healthy	○			○	○
128	2001	5	15-34	60%	Biopsy	Minimal change neph.	● 100% +				
18	2019	4	Unkn.	Unkn.	Unkn.	Minimal change neph.		● 100% +	○ 0%	● 100% ++	●
18	2019	4	Unkn.	Unkn.	Unkn.	Thin basement dis.		● 100% ±	● 25% −	● 75% ±	●
28	2018	62	27-79[c]	66%	Biopsy	Diabetic neph.	●			●	●
112	2002	35	23-71	66%	Biopsy	Membranous neph.		● 23%	● 100%	● 100%	● 80%
148	2004	20	44-57[c]	70%	Biopsy	Membranous neph.	● 100%				
156	2010	24	28-75	67%	Biopsy	Membranous neph.			●		
150	2011	8	39-77	38%	Biopsy	Membranous neph.			●		
153	2014	Unkn.	Unkn.	Unkn.	Unkn.	Membranous neph.	●				
18	2019	5	Unkn.	Unkn.	Unkn.	Membranous neph.		○ 0%	● 100% +++	● 50% ±	●

110	1995	20	19-45	60%	Biopsy	IgA neph.	●	95%								●	90%		●	75%	
58	1995	2	Unkn.	Unkn.	Biopsy	IgA neph.	●														
160	1998	45	15-48	47%	Biopsy	IgA neph.	●	100%													
99	2000	10	9-23	40%	Biopsy	IgA vasc.	●	100%													
128	2001	14	20-51	43%	Biopsy	IgA neph.	●	100%	++												
115	2008	30	17-67[d]	75%[d]	Biopsy	IgA neph.			+/++	●	100%		●	100%		●	100%		●	81%	
165	2017	96	4-66[c]	65%	Biopsy	IgA neph.				●	100%										
124	2017	25	0-63[c]	80%	Biopsy	IgA neph.	●	100%	+												
46	1985	1	18	0%	Unkn.	Lupus nephritis	●														
58	1995	2	Unkn.	Unkn.	Biopsy	Lupus nephritis IV	●														
172	2008	1	27	0%	Biopsy	Lupus nephritis V							●								
18	2019	20	20-64	20%	Biopsy	Lupus nephritis III				●	85%	+	●	65%	+	●	70%	+	●		
		22	21-61	13%	Biopsy	Lupus nephritis IV				●	87%	+/++	●	59%	+/++	●	82%	+	●		
		13	18-71	23%	Biopsy	Lupus nephritis V				●	46%	−	●	100%	+++	●	69%	−	●		
103	2012	2	20-42[d]	100%	Biopsy	C3GN				●	100%	+++	●	100%	+++	●	100%	+++	●	100%	+++
		2	20-42[d]	100%	Biopsy	DDD				●	100%	+++	●	100%	+++	●	100%	+++	●	100%	+++
102	2019	24	9-74[d]	52%[d]	Biopsy	C3G	●	100%	+/++												
		8	9-74[d]	52%[d]	Biopsy	C3G	●	100%	++												
103	2012	1	Unkn.	Unkn.	Biopsy	aHUS				●		+++	●		+++	●		+++	●		
153	2014	10	22-44[d]	18%[d]	Biopsy	STEC	○														
188	2015	1	16	100%	Biopsy	aHUS				●			●						●		
132	2018	5	9-40	60%	Biopsy	TTP				○	0%		●	40%					●	60%	
		8	22-66	Unkn.	Autopsy or biopsy	TTP				○	0%		●	20%					●	40%	
100	2019	6	22-65	50%	Biopsy	TMA				●	100%	++	●	33%	+/++	●			●	100%	+++
		7	32-72	29%	Biopsy	TMA				●	100%	+/++	●	14%	++	●			●	100%	+++
195	2006	34	25-80	50%	Biopsy	ANCA-vasculitis	●		++							●			●		+
196	2008	34	>15	50%	Biopsy	ANCA-vasculitis	●									●					
31	2019	54	18-76[c]	43%	Biopsy	Interstitial nephritis										●					
31	2019	5	25-65	60%	Biopsy	Acute tubular necrosis										●					
125	2020	6	51-86	67%	Autopsy	Acute tubular necrosis	●	33%								●	100%	+/++	●	33%	
209	2000	31	Unkn.	Unkn.	Nephrect.	Renal cell carcinoma	●[h]	16%[h]	+[h]												
207	2020	20	37-81[c]	57%	Nephrect.	Renal cell carcinoma	○[h]														
129	2003	37	20-77	57%	Biopsy	After transplantation				●			○			●					
104	2009	1	20	100%	Biopsy	Transplant rejection										●			●		
211	2013	33	10-83[c]	61%	Biopsy	Reperfusion										○			○		
		1	Unkn.	Unkn.	Biopsy	Transplant rejection										●			●		

**Anti-C5b-9(m)**																		
96	1986	6	Adults	Unkn.	Biopsy	Healthy	●								●			●
		2	Adults	Unkn.	Nephrect.	Healthy	●								●			●
72	1989	3	Adults	Unkn.	Biopsy	Healthy			●			○			●			●
96	1986	5	Adults	Unkn.	Unkn.	Minimal change neph.			●	100%	+	○	0%		●			●
141	1986	3	2-14[d]	Unkn.	Biopsy	Minimal change neph.			○			○			○			○
98	1987	3	2-14[d]	Unkn.	Biopsy	Minimal change neph.			○			○			○			○
72	1989	3	Adults	Unkn.	Unkn.	Minimal change neph.			●			○			●			●
96	1986	1	Adult	Unkn.	Unkn.	Alport's syndr.			●			○			●			●
96	1986	2	Adults	Unkn.	Unkn.	Diabetic neph.			●	100%	++	○	0%		●			●
72	1989	3	Adults	Unkn.	Unkn.	Diabetic neph.	●								●			●
96	1986	6	Adults	Unkn.	Unkn.	Membranous neph.			○	0%		●	100%	++	●			●
		9	Adults	Unkn.	Unkn.	Membranous neph.			●	78%	+	●	22%	+/++	●			●
141	1986	2	2-14[d]	Unkn.	Biopsy	Membranous neph.						●	100%	++				
98	1987	2	2-14[d]	Unkn.	Biopsy	Membranous neph.	●	100%										
72	1989	7	Adults	Unkn.	Unkn.	Membranous neph.	●	100%							●			●
96	1986	7	Adults	Unkn.	Unkn.	IgA neph.			●	100%	+/++	●	14%	+	●			●
		2	Adults	Unkn.	Unkn.	IgA vasc.			●	100%	+	○	0%		●			●
72	1989	10	Adults	Unkn.	Unkn.	IgA neph.	●								●			●
58	1995	2	Unkn.	Unkn.	Biopsy	IgA neph.	●											
96	1986	1	Adult	Unkn.	Biopsy	Lupus nephritis II			●		+	○			●			●
		8	Adults	Unkn.	Biopsy	Lupus nephritis IV			●	100%	++	●	88%	++	●			●
72	1989	7	Adults	Unkn.	Unkn.	Lupus npehritis II-IV	●	100%							●			●
58	1995	2	Unkn.	Unkn.	Biopsy	Lupus nephritis IV	●											
96	1986	9	Adults	Unkn.	Biopsy	MPGN I			●	89%	++	●	100%	++	●			●
141	1986	2	2-14[d]	Unkn.	Biopsy	MPGN			○	0%		●	50%	+	●	50%	+	
98	1987	2	2-14[d]	Unkn.	Biopsy	MPGN	●	50%										
72	1989	5	Adults	Unkn.	Unkn.	MPGN I	●	100%							●			●
96	1986	1	Adult	Unkn.	Biopsy	Interstitial nephritis			●	100%	+	○	0%		●			●
96	1986	2	Adults	Unkn.	Biopsy	Transplant rejection			●	100%	+	○	0%		●			●
214	1986	13	10-43	38%	Biopsy	*De novo* membranous neph. in transplant			●	40%		●	60%					

**Anti-MAC**																					
57	1989	6	Unkn.	Unkn.	Biopsy	Minimal change neph.				●			○			●			●		
57	1989	22	Unkn.	Unkn.	Biopsy	Membranous neph.	●		++							●			●		
57	1989	75	Unkn.	Unkn.	Biopsy	IgA neph.	●		+/++							●			●		
57	1989	20	Unkn.	Unkn.	Biopsy	Lupus nephritis	●	79%								●			●		
57	1989	11	Unkn.	Unkn.	Biopsy	MPGN I	●									●			●		
		1	Unkn.	Unkn.	Biopsy	MPGN II	●									●			●		
**Anti-MAC-neo**																					
52	1987	4	5-37	75%	Biopsy	Healthy				○	0%		○	0%							
52	1987	23	6-56	78%	Biopsy	IgA neph.				●	100%	++	●	56%	++	●	76%		●	76%	
		2	9-10	50%	Biopsy	IgA vasc.				●	100%	+/++	●	100%	+/++						
**bC5 or A239**																					
130	2015	9	Unkn.	Unkn.	Unkn.	Healthy													●	78%	
79	1994	2	18-23	50%	Biopsy	Minimal change neph.				●		+	○			●		+++			
130	2015	5	Unkn.	Unkn.	Biopsy	Alport's syndr.	○	0%											○	0%	
79	1994	6	47-65	33%	Biopsy	Membranous neph.				●		+	●		+++	●		+			
130	2015	1	32	100%	Biopsy	IgA neph.	○												○		
167	2019	116	0-84	69%	Biopsy	IgA neph. or vasc.	●	15%											●	50%	
130	2015	8	17-49	0%	Autopsy or biopsy	TMA	●	60%											●	60%	
130	2015	11	22-77	18%	Autopsy or biopsy	aHUS	●												●		
		1	14	100%	Biopsy	STEC	○												○		
		6	18-54	50%	Autopsy or biopsy	TMA	●												●		
		3	6-37	67%	Biopsy or nephrect.	aHUS	●												●		
130	2015	1	35	100%	Biopsy	ANCA-vasculitis	●									●			●		
**B7**																					
134	2019	1	Unkn.	Unkn.	Biopsy	Healthy				○			○						●		
58	1995	2	Unkn.	Unkn.	Biopsy	IgA neph.	●														
58	1995	2	Unkn.	Unkn.	Biopsy	Lupus nephritis IV	●														
134	2019	54	21-69[c]	63%	Biopsy	Transplant rejection				○	0%		●	24%	+	●			●	2%	++

**Kolb 1975**																					
95	1981	3	Unkn.	Unkn.	Unkn.	Healthy										○					
95	1981	2	Unkn.	Unkn.	Unkn.	Hypertensive neph.										○					
95	1981	2	Unkn.	Unkn.	Autopsy or biopsy	Lupus nephritis II				●	100%		●	100%		●	100%	+	●		
		7	Unkn.	Unkn.	Autopsy or biopsy	Lupus nephritis III				●	86%	+	●	86%	+	●	100%	+/++	●		
		10	Unkn.	Unkn.	Autopsy or biopsy	Lupus nephritis IV				●	100%	++	●	100%	++	●	100%	++	●		
		3	Unkn.	Unkn.	Autopsy or biopsy	Lupus nephritis V				○	0%		●	67%	+/++	●	100%	++	●		
**PolyC9-MA**																					
44	1983	Unkn.	Fetus	Unkn.	Unkn.	Healthy				○			○			○			○		
		Unkn.	Adults	Unkn.	Unkn.	Healthy				●			○			○			●		
67	1987	6	Unkn.	Unkn.	Biopsy	Healthy				●		±	○			●		+	●		+/++
126	1987	1	0	Unkn.	Autopsy or unkn.	Healthy				●			○			○			●		
		2	55-65	Unkn.	Autopsy or unkn.	Healthy				●			●			●			●		
127	1987	4	Unkn.	Unkn.	Unkn.	Healthy				●			○			●			●		
137	1987	2	Unkn.	Unkn.	Nephrect.	Healthy	○	0%											●	100%	+++
97	1988	4	43-66	Unkn.	Unkn.	Healthy				●			○			●			●		
75	1993	Unkn.	Unkn.	Unkn.	Nephrect.	Healthy				●			○			●			●		
138	1996	10	Unkn.	Unkn.	Unkn.	Healthy													●		
67	1987	7	Unkn.	Unkn.	Biopsy	Minimal change neph.				●		±	○			●		+	●		+/++
81	1991	9	Unkn.	Unkn.	Biopsy	Minimal change neph.				●			○								
75	1993	10	Unkn.	Unkn.	Biopsy	Minimal change neph.				●			○			●			●		
44	1983	3	Unkn.	Unkn.	Biopsy or nephrect.	Hypertensive neph.				●			○			●			●		
67	1987	6	Unkn.	Unkn.	Biopsy	Hypertensive neph.				●		+	○			●		+/++	●		+++
44	1983	7	Unkn.	Unkn.	Biopsy or nephrect.	Diabetic neph.				●			○			●			●		
67	1987	9	Unkn.	Unkn.	Biopsy	Diabetic neph.				●		+	○			●		++	●		++
126	1987	12	Unkn.	Unkn.	Biopsy or nephrect.	Diabetic neph.				●			●	25%		●			●		
44	1983	3	Unkn.	Unkn.	Biopsy	Membranous neph.				○			●			●					

67	1987	11	Unkn.	Unkn.	Biopsy	Membranous neph.							●		++	●		+/++	●		+++
152	1989	2	5-15	100%	Biopsy	Membranous neph.				●			●	100%							
		6	3-13	33%	Biopsy	Membranous neph.				●			●	83%							
81	1991	18	Unkn.	Unkn.	Biopsy	Membranous neph.	●														
75	1993	2	Unkn.	Unkn.	Biopsy	Membranous neph.							●			●					
44	1983	3	Unkn.	Unkn.	Biopsy	IgA neph.				●			○			●					
137	1987	12	Unkn.	Unkn.	Biopsy	IgA neph.	●	100%	++										●	100%	++
67	1987	11	Unkn.	Unkn.	Biopsy	IgA neph.				●		+++	○			●		+/++	●		+++
97	1988	30	4-17	70%	Biopsy	IgA neph.		73%		●			●			●	93%				
81	1991	31	Unkn.	Unkn.	Biopsy	IgA neph.	●														
93	1991	1	23	100%	Biopsy	IgA neph.	●		+												
75	1993	22	Unkn.	Unkn.	Biopsy	IgA neph.				●						●					
		7	Unkn.	Unkn.	Biopsy	IgA vasc.				●						●					
44	1983	3	Unkn.	Unkn.	Biopsy	Lupus nephritis				●			●			●					
67	1987	8	Unkn.	Unkn.	Biopsy	Lupus nephritis III, IV				●		++	●		++	●		+/++	●		+/++
152	1989	1	18	0%	Biopsy	Lupus nephritis V							●								
81	1991	9	Unkn.	Unkn.	Biopsy	Lupus nephritis IV	●														
75	1993	4	Unkn.	Unkn.	Biopsy	Lupus nephritis IV				●			●			●					
44	1983	3	Unkn.	Unkn.	Biopsy	MPGN I										●					
		2	Unkn.	Unkn.	Nephrect.	MPGN II				●			●			●					
183	1984	11	5-14	45%	Biopsy	Postinfectious				●	86%		●	86%							
67	1987	3	Unkn.	Unkn.	Biopsy	MPGN I				●		+++	●		++	●		+/++	●		++
		4	Unkn.	Unkn.	Biopsy	Postinfectious				●		++	●		++	●		++	●		++
126	1987	3	Unkn.	Unkn.	Biopsy	MPGN II				●			●			●					
75	1993	1	Unkn.	Unkn.	Biopsy	MPGN I				●			●			●					
		2	Unkn.	Unkn.	Biopsy	MPGN II				●			●			●					
		1	Unkn.	Unkn.	Biopsy	MPGN III				●			●			●					
203	1993	1	21	100%	Biopsy	Acute tubular necrosis	○									●			●		
44	1983	3	Unkn.	Unkn.	Biopsy or nephrect.	Reflux neph.				●			○			●			●		
127	1987	8	8-51	13%	Biopsy or nephrect.	Reflux neph.	●									●			●		
75	1993	1	Unkn.	Unkn.	Biopsy	Reflux neph.	●														
138	1996	10	Unkn.	Unkn.	Unkn.	Renal cell carcinoma	●[h]	10%[h]	+[h]										●	60%	++
67	1987	4	Unkn.	Unkn.	Biopsy	Transplant rejection				●		+/++	○			●		+/++	●		+/++

**WU-7,2**																
111	1996	7	Unkn.	Unkn.	Nephrect.	Healthy				●	43%	+	○	0%	○	●
111	1996	5	Unkn.	Unkn.	Biopsy	Lupus nephritis II			+/++	●	100%		○	0%		
		3	Unkn.	Unkn.	Biopsy	Lupus nephritis II			+++	●	100%		○	0%		
		7	Unkn.	Unkn.	Biopsy	Lupus nephritis IV			+++	●	100%		●	100%		
**WU-13,15**																
119	2013	5	Unkn.	Unkn.	Autopsy	Healthy	○								○	○
119	2013	11	23-41	27%	Biopsy	Lupus nephritis II-V									●	●
		2	23-41[d]	27%[d]	Biopsy	Lupus nephritis II	●	100%	+++							
		3	23-41[d]	27%[d]	Biopsy	Lupus nephritis III	●	100%	+/++							
		3	23-41[d]	27%[d]	Biopsy	Lupus nephritis IV	●	100%	+++							
		3	23-41[d]	27%[d]	Biopsy	Lupus nephritis V	●	100%	+/++							
190	2013	1	26	0%	Biopsy	STEC	●									
**X197**																
171	2018	30	8-59[c]	20%	Biopsy	Lupus nephritis II-V									●	●
		2	8-59[cd]	20%[d]	Biopsy	Lupus nephritis II	○	0%								
		5	8-59[cd]	20%[d]	Biopsy	Lupus nephritis III	●	20%								
		8	8-59[cd]	20%[d]	Biopsy	Lupus nephritis IV	●	50%								
		5	8-59[cd]	20%[d]	Biopsy	Lupus nephritis V	●	60%								
		8	8-59[cd]	20%[d]	Biopsy	Lupus nephritis III+V	●	50%								
		2	8-59[cd]	20%[d]	Biopsy	Lupus nephritis IV+V	●	50%								
**Xia 1988**																
61	1990	3	Unkn.	Unkn.	Biopsy	Minimal change neph.	○	0%								
61	1990	4	Unkn.	Unkn.	Biopsy	IgA vasc.	●	100%	+/++							
**1B4**																
114	2006	7	Children	Unkn.	Unkn.	Healthy				○	0%		○	0%		
114	2006	18	Children	56%[d]	Biopsy	MPGN I		94%	+/++	●			●			

For a legend, see the legend of Supplementary Table 2.

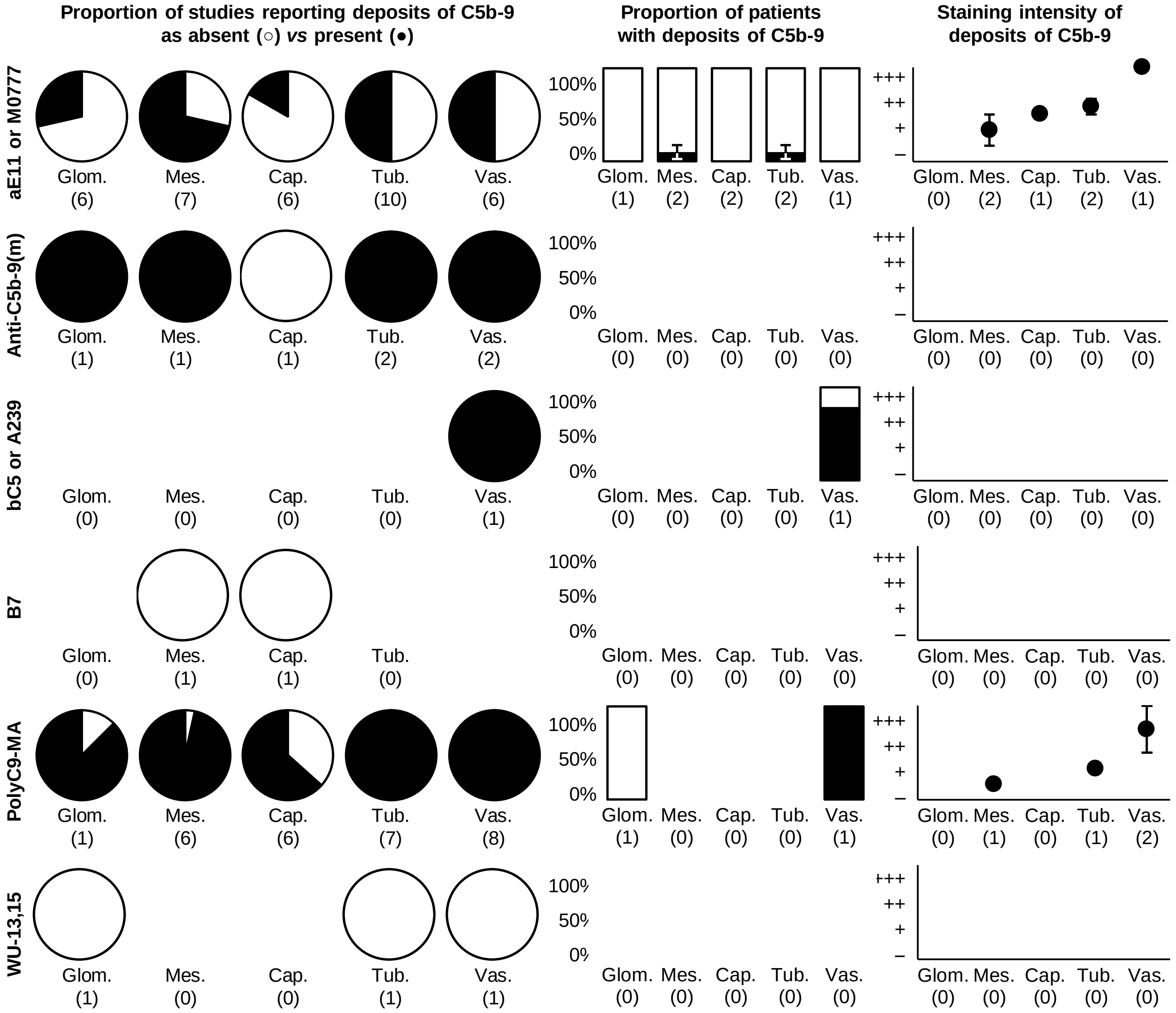

SUPPLEMENTARY FIGURE 1 | Deposits of C5b-9 in healthy human kidneys as detected with different antibodies. Pie charts show the proportion of studies that reported staining of C5b-9 as absent (light) or present (dark). Bar charts show the medians of the proportions of patients reported to exhibit staining. Scatter charts show the median staining intensities in these patients. All charts show data separately for staining in the glomerulus as a whole (glom.), in the mesangium (mes.), along the glomerular capillary wall (cap.), along the tubular basement membrane (tub.), or in the extraglomerular vascular wall (vas.). Error bars show the lowest and highest reported values. Numbers of studies are indicated between brackets. Some studies reported only part of the data shown, explaining differences in the numbers of studies between pie, bar, and scatter charts. Nothing is indicated if the data were never reported. Detailed data per study are listed in Supplementary Table 3. Data on antibodies used in only one original study are only listed in Supplementary Table 2. Antibodies' names correspond with those specified in Table 2.

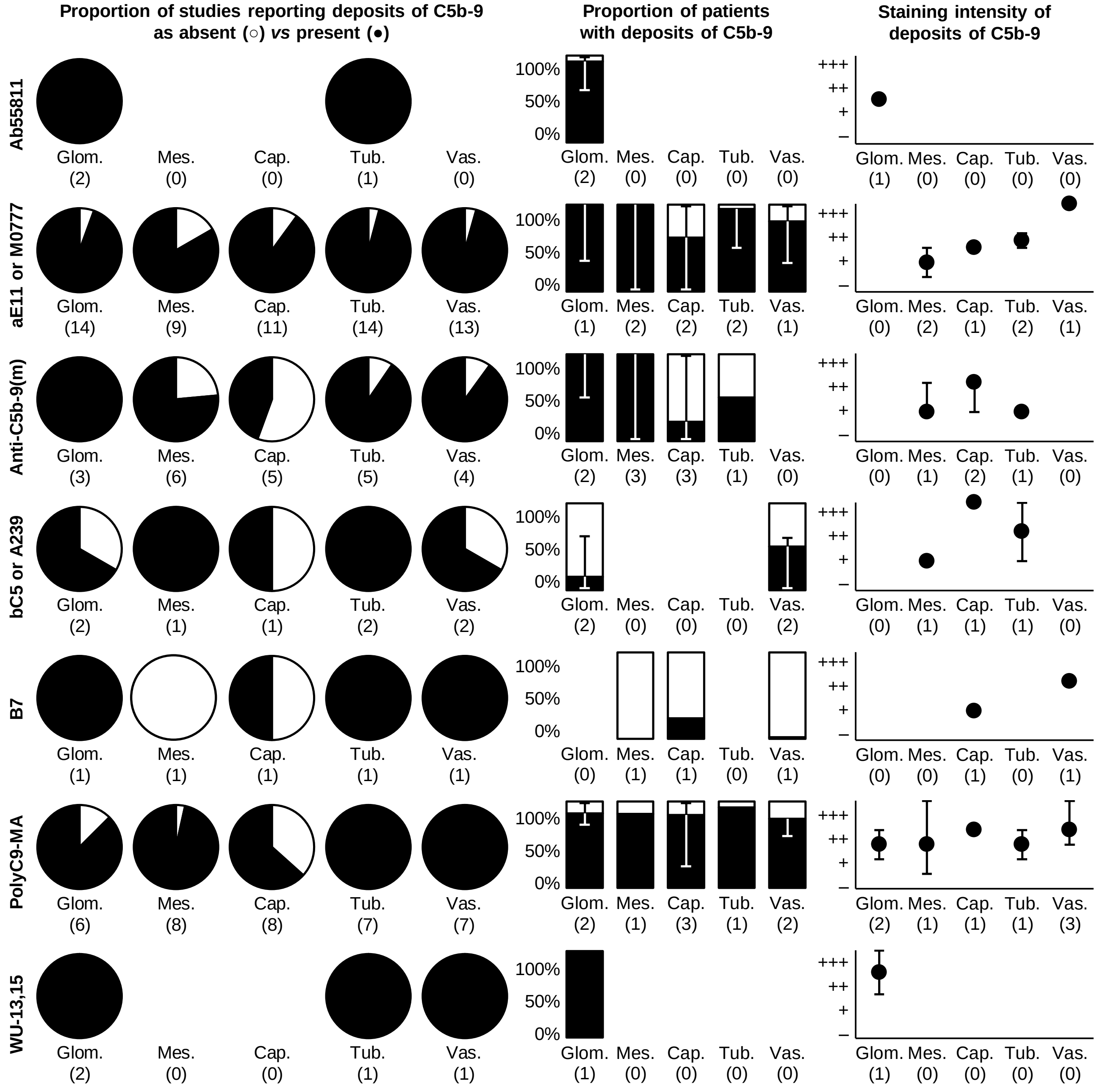

SUPPLEMENTARY FIGURE 2 | Deposits of C5b-9 in diseased human kidneys as detected with differrent antibodies. Pie charts show the proportion of studies that reported staining of C5b-9 as absent (light) or present (dark). Bar charts show the medians of the proportions of patients reported to exhibit staining. Scatter charts show the median staining intensities in these patients. All charts show data separately for staining in the glomerulus as a whole (glom.), in the mesangium (mes.), along the glomerular capillary wall (cap.), along the tubular basement membrane (tub.), or in the extraglomerular vascular wall (vas.). Error bars show the lowest and highest reported values. Numbers of studies are indicated between brackets. Some studies reported only part of the data shown, explaining differences in the numbers of studies between pie, bar, and scatter charts. Nothing is indicated if the data were never reported. Detailed data per study are listed in Supplementary Table 3. Data on antibodies used in only one original study are only listed in Supplementary Table 2. Antibodies' names correspond with those specified in Table 2. Note that the data reflect various kidney diseases.

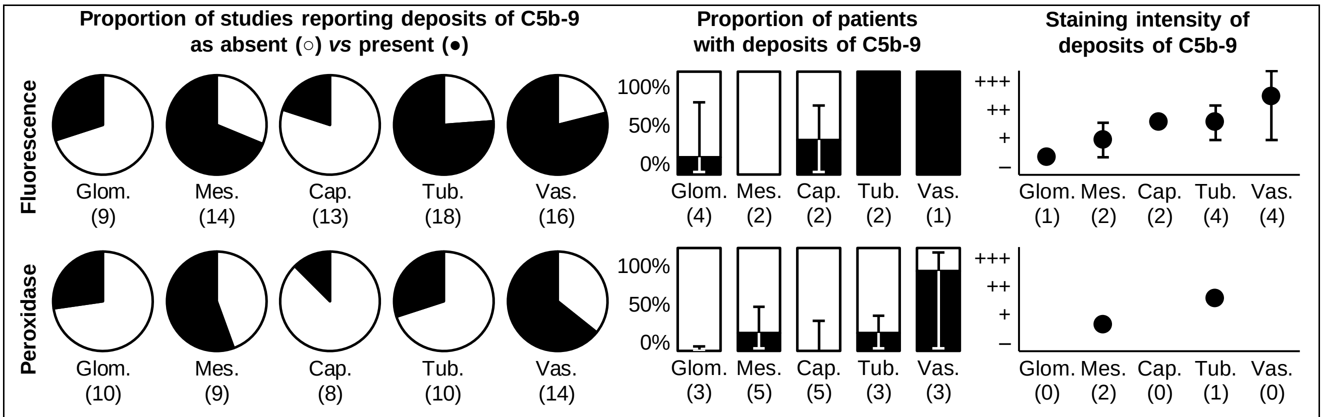

SUPPLEMENTARY FIGURE 3 | Deposits of C5b-9 in healthy human kidneys as detected with different staining techniques. Pie charts show the proportion of studies that reported staining of C5b-9 as absent (light) or present (dark). Bar charts show the medians of the proportions of patients reported to exhibit staining. Scatter charts show the median staining intensities in these patients. All charts show data separately for staining in the glomerulus as a whole (glom.), in the mesangium (mes.), along the glomerular capillary wall (cap.), along the tubular basement membrane (tub.), or in the extraglomerular vascular wall (vas.). Error bars show the lowest and highest reported values. Numbers of studies are indicated between brackets. Some studies reported only part of the data shown, explaining differences in the numbers of studies between pie, bar, and scatter charts. Nothing is indicated if the data were never reported. Detailed data per study are listed in Supplementary Table 2.

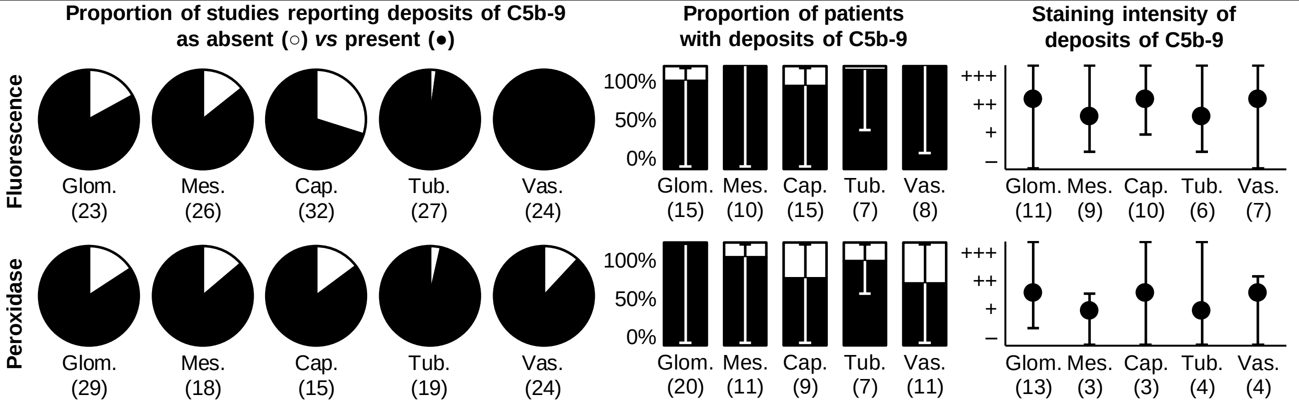

SUPPLEMENTARY FIGURE 4 | Deposits of C5b-9 in diseased human kidneys as detected with different staining techniques. Pie charts show the proportion of studies that reported staining of C5b-9 as absent (light) or present (dark). Bar charts show the medians of the proportions of patients reported to exhibit staining. Scatter charts show the median staining intensities in these patients. All charts show data separately for staining in the glomerulus as a whole (glom.), in the mesangium (mes.), along the glomerular capillary wall (cap.), along the tubular basement membrane (tub.), or in the extraglomerular vascular wall (vas.). Error bars show the lowest and highest reported values. Numbers of studies are indicated between brackets. Some studies reported only part of the data shown, explaining differences in the numbers of studies between pie, bar, and scatter charts. Nothing is indicated if the data were never reported. Detailed data per study are listed in Supplementary Table 2. Note that the data reflect various kidney diseases.

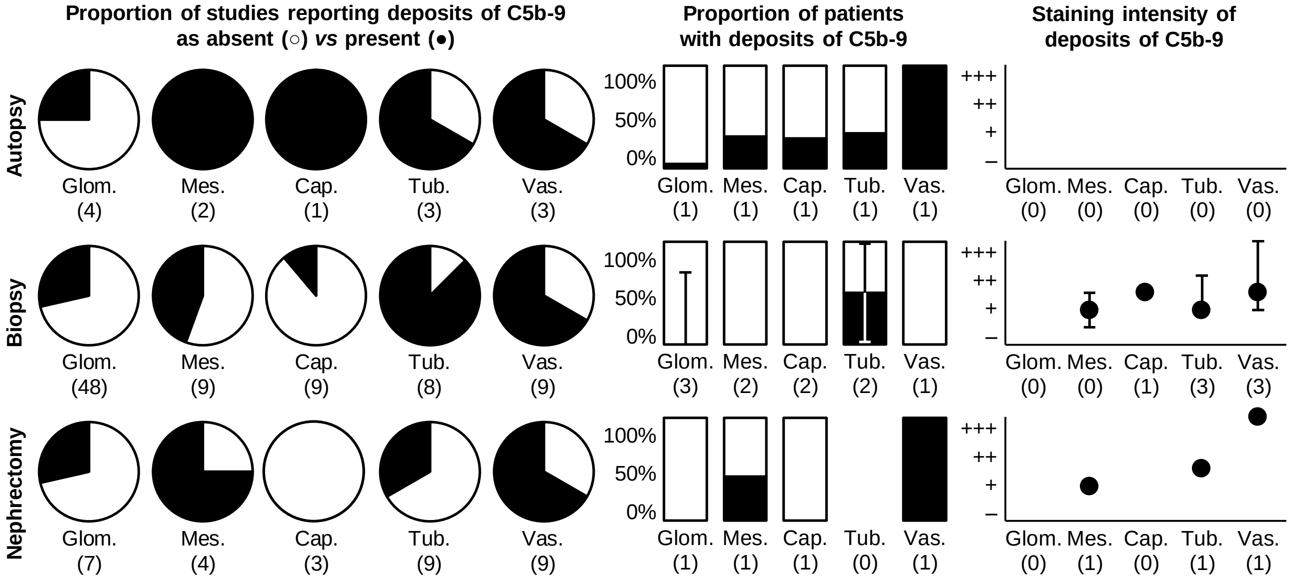

SUPPLEMENTARY FIGURE 5 | Deposits of C5b-9 in healthy human kidneys as detected in different tissue sources. Pie charts show the proportion of studies that reported staining of C5b-9 as absent (light) or present (dark). Bar charts show the medians of the proportions of patients reported to exhibit staining. Scatter charts show the median staining intensities in these patients. All charts show data separately for staining in the glomerulus as a whole (glom.), in the mesangium (mes.), along the glomerular capillary wall (cap.), along the tubular basement membrane (tub.), or in the extraglomerular vascular wall (vas.). Error bars show the lowest and highest reported values. Numbers of studies are indicated between brackets. Some studies reported only part of the data shown, explaining differences in the numbers of studies between pie, bar, and scatter charts. Nothing is indicated if the data were never reported. Detailed data per study are listed in Supplementary Tables 2 and 3.

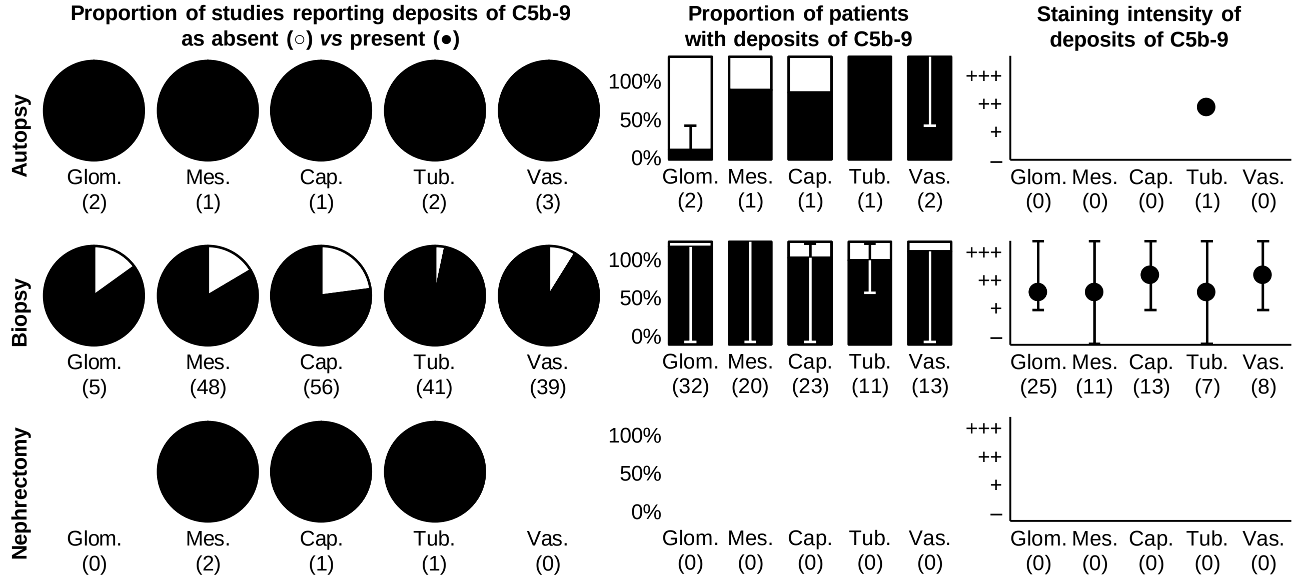

SUPPLEMENTARY FIGURE 6 | Deposits of C5b-9 in diseased human kidneys as detected in different tissue sources. Pie charts show the proportion of studies that reported staining of C5b-9 as absent (light) or present (dark). Bar charts show the medians of the proportions of patients reported to exhibit stai-ning. Scatter charts show the median staining intensities in these patients. All charts show data separately for staining in the glomerulus as a whole (glom.), in the mesangium (mes.), along the glomerular capillary wall (cap.), along the tubular basement membrane (tub.), or in the extraglomerular vascular wall (vas.). Error bars show the lowest and highest reported values. Numbers of studies are indicated between brackets. Some studies reported only part of the data shown, explaining differences in the numbers of studies between pie, bar, and scatter charts. Nothing is indicated if the data were never reported. Detailed data per study are listed in Supplementary Tables 2 and 3. Note that the data reflect various kidney diseases.
